# Switching on Supramolecular DNA Junction Binding Using a Human Enzyme

**DOI:** 10.1002/anie.202503683

**Published:** 2025-03-23

**Authors:** Subhendu Karmakar, Samuel J. Dettmer, Catherine A. J. Hooper, Nikolas J. Hodges, Michael J. Hannon

**Affiliations:** ^1^ School of Chemistry University of Birmingham Edgbaston Birmingham B15 2TT UK; ^2^ School of Biosciences University of Birmingham Edgbaston Birmingham B15 2TT UK

**Keywords:** Bio‐inorganic, DNA‐recognition, DNA three‐way junctions, Enzyme‐mediated activation, Metallo‐supramolecular chemistry

## Abstract

Non‐canonical DNA junction structures are important in human disease and in nucleic acid nanoscience and there is a growing interest in how to bind and modulate them. A key next step is to exert “on command” control over such binding. Herein we develop a new metallo‐supramolecular triple‐helicate cylinder agent that is inert to DNA junction binding until activated by human enzyme NAD(P)H:quinone oxidoreductase 1 (NQO1) and its cofactor nicotinamide adenine dinucleotide phosphate (NADPH). This inactive cylinder bears six flexible arms each with a quinone group at the termini. Reduction by the enzyme leads to all six arms being removed, transforming the inert cylinder into a new and active metallo‐supramolecular agent that binds junctions. This gives the ability to “switch‐on” DNA junction formation and binding in response to the presence of two external stimuli – a human enzyme overexpressed in many disease states, and NADPH – and absence of inhibitor, giving NAND logic control. Modelling indicates the binding activation originates not in steric unblocking but changes in conformational flexibility. The work provides the foundation for and a route map toward future designs of sophisticated, inert, and supramolecular structures which are transformed by enzymes into new, active, and supramolecular structures for a variety of potential applications.

## Graphical abstract

Stimulus responsive control of DNA junction formation: Enzyme action cleaves flexible arms from a metallo‐supramolecular cylinder, transforming an inert metallo‐supramolecular structure into an active metallo‐supramolecular agent, and switching on its ability to assemble, bind and stabilize DNA three‐way junction structures.

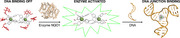

Small molecule duplex DNA binders remain the most widely used clinical compounds against cancers. DNA alkylators crosslink DNA by covalent (nitrogen mustards and nitrosoureas)^[^ ^1^, ^2^ ^]^ or coordination (platins)^[^ ^2^, ^3^, ^4^, ^5^, ^6^ ^]^ bond formation with DNA bases, while intercalators (anthracyclines)^[^ ^7^, ^8^ ^]^ and groove binders (polyamides)^[^ ^9^, ^10^ ^]^ bind non‐covalently. However, the prevalence of the duplex DNA structure in most cells, means that the action of these agents is not sufficiently targeted and side‐effects are common. A promising alternative is to bind other, less common, noncanonical DNA structures^[^ ^11^, ^12^, ^13^, ^14^, ^15^, ^16^, ^17^, ^18^, ^19^, ^20^, ^21^, ^22^, ^23^, ^24^, ^25^ ^]^ such as triplexes,^[^ ^16^, ^18^, ^19^ ^]^ quadruplexes,^[^ ^14^, ^15^, ^16^, ^17^, ^22^ ^]^ junctions,^[^ ^11^, ^12^, ^13^, ^16^ ^]^ and i‐motifs.^[^ ^23^, ^24^ ^]^ Such structures play key roles in DNA transactions and in viral and genetic diseases.^[^ ^20^, ^26^, ^27^, ^28^ ^]^

During replication, transcription, recombination, and repair, DNA adopts diverse junction structures. The simplest such junction is a three‐way junction (3WJ) where three duplex arms converge at a branch point, with a range of other Y‐shaped DNA structures (including replication and transcription forks) also known.^[^ ^12^, ^13^ ^]^ Synthetic molecules that stabilize DNA Y‐shaped structures, have the potential to interfere with polymerase activity and replication.^[^ ^29^, ^30^, ^31^, ^32^, ^33^, ^34^, ^35^ ^]^ We have shown (X‐ray crystallography^[^ ^36^, ^37^, ^38^, ^39^ ^]^ and NMR studies^[^ ^40^ ^]^) that nanosized tetracationic metallo‐supramolecular cylinders (Figure 1) are a perfect fit for the cavity at the heart of a 3WJ with π‐surfaces that stack with the three DNA base pairs at the junction point. Organic cryptands that present the same π‐surfaces also bind the junction,^[^ ^30^, ^41^, ^42^, ^43^ ^]^ as do some other metallo‐supramolecular helicates and mesocates.^[^ ^31^, ^44^, ^45^, ^46^, ^47^, ^48^, ^49^, ^50^ ^]^

**Figure 1:**
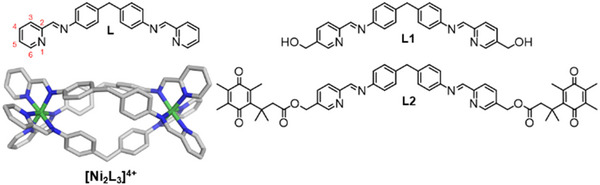
Ligands used in this work and X‐ray crystal structure of Ni parent cylinder, [Ni_2_L_3_]^4+^ (CCDC: NITBIB),^[^ ^39^ ^]^ hydrogens omitted for clarity.

Recently, we demonstrated that by wrapping a curcubituril macrocycle around the π‐surfaces of the metallo‐cylinders and then locking the macrocycle in place by rotaxanation, we could prevent the cylinders from binding into the 3WJ.^[^ ^51^ ^]^ Kinetic dethreading (in systems that were not fully stoppered) allowed the reactivation of DNA binding over time. A key next goal is to achieve better control over the binding so as to be able to “switch‐on” an inert supramolecular agent in response to a stimulus: herein we develop cylinders whose DNA junction binding is activated in responsive to an enzyme. We exploit NAD(P)H:quinone oxidoreductase 1 (NQO1) to activate a projunction binder (Scheme 1). NQO1 is a cytosolic enzyme upregulated in response to stress and present in elevated levels in many human cancer cells (e.g., lung,^[^ ^52^, ^53^, ^54^ ^]^ breast,^[^ ^55^, ^56^, ^57^, ^58^ ^]^ pancreas,^[^ ^59^, ^60^, ^61^ ^]^ colon,^[^ ^62^, ^63^, ^64^ ^]^ stomach,^[^ ^65^ ^]^ ovary,^[^ ^66^ ^]^ prostate,^[^ ^67^ ^]^ and cervix^[^ ^68^ ^]^). The result is, for the first time, an enzyme‐responsive DNA junction binder.

**Scheme 1:**
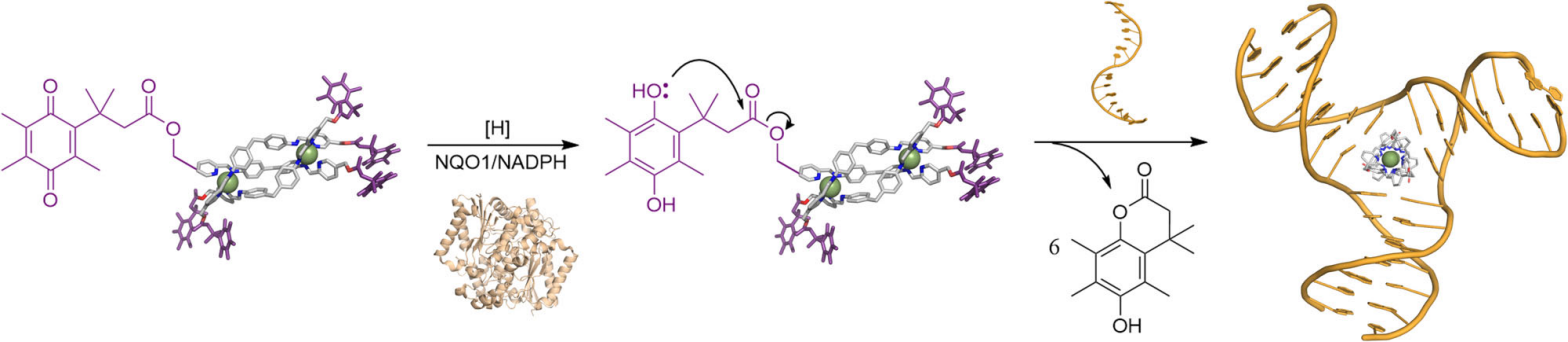
NQO1 mediated activation of supramolecular DNA junction binder.

We identified a trimethyl‐substituted quinone linked to β‐dimethyl substituted propionic acid (QPA)^[^ ^69^ ^]^ as an appropriate NQO1 responsive unit that could be added to our metallo‐cylinder. This QPA derivative has been used in NQO1‐activated anticancer prodrugs,^[^ ^70^, ^71^, ^72^, ^73^ ^]^ nanodrug delivery systems,^[^ ^74^, ^75^, ^76^, ^77^ ^]^ imaging,^[^ ^78^, ^79^ ^]^ and theranostic agents.^[^ ^70^, ^80^, ^81^, ^82^, ^83^, ^84^, ^85^ ^]^ Quinone reduction by the enzyme is followed by lactone ring closure, and release (Scheme 1).^[^ ^86^, ^87^ ^]^

The concept is that attached large QPA groups should interfere with the DNA binding, until removed by NQO1. Of the possible attachment positions on the bis‐pyridylimine ligand (ligand, L) we identified the 4‐ and 5‐pyridyl positions as suitable and started with the 5‐position, which is synthetically more accessible and where we have previously functionalized cylinders.^[^ ^88^ ^]^ Although the 5‐position will initially orient substituents out from the ends of the cylinder, using a methylene hydroxy (Figure 1) twists the substituent so it projects out from the cylinder or back across the cylinder structure.

The metallo‐cylinder was prepared (Supporting Information Scheme S1 and experimental details), by first attaching the QPA to 5‐hydroxymethyl‐2‐pyridinecarboxaldehyde, and then condensing with the 4,4′‑methylenedianiline (MDA) spacer to form the QPA substituted di‐imine helicate ligand (L2), which readily formed the triple stranded metallo‐cylinder [M_2_(L2)_3_]^4+^ (M = Fe, Ni) upon heating with an appropriate metal salt in methanol. As controls, the complexes [M_2_(L1)_3_]^4+^ (without the QPA attached) were also prepared. The ESI‐MS of all the complexes show the expected dominant [M_2_L_3_]^4+^ species, together with smaller peaks corresponding to the same cation with associated anions (Figures S1–S8). Proton NMR spectra of the diamagnetic iron(II) complexes (Figures S17–S24) show the expected shift patterns for metal coordination^[^ ^38^, ^39^, ^89^ ^]^ and also confirmed that a helicate structure had been formed through the observation of a singlet for the central methylene combined with a pair of diastereotopic protons on the other methylene where the QPA is attached. The characteristic shifts and broadenings of the aromatic protons of the diaryl methane spacer, which are face‐edge π‐stacked and constrained within the helicate structure, were observed. Temporal UV–vis studies indicated that the nickel complex had better solubility and better stability (Figures S26–S27) in aqueous buffer and so this cylinder was explored further.

We next explored the ability of the NQO1 enzyme to remove these six QPA groups from the cylinder. We reacted QPA capped [Ni_2_(L2)_3_]^4+^ cylinder with NQO1 (20 µg mL^−1^) and NADPH (8 equivs; cofactor) in water at 37 °C and monitored the reaction using ESI‐MS. A decline in the intensity of the molecular ion [Ni_2_(L2)_3_]^4+^ (*m*/*z =* 704.7) peak was observed and a new peak of increasing intensity corresponding to the uncapped [Ni_2_(L1)_3_]^4+^ (*m*/*z =* 356.6) indicated the removal of QPA cap catalyzed by NQO1 (Figure 2). The growing peaks at *m*/*z* 235.1 ([M + H]^+^) and *m*/*z* 257.1 ([M + Na]^+^) also confirmed the release of the lactone by‑product following the QPA reduction. Interestingly, besides a small molecular ion peak for a 5‐capped cylinder (*m*/z 646.7) no subcapped metallo‐cylinder species were identified (Figure 2). It appears that once the 6‐capped cylinder encounters the enzyme, the catalysis reactions occur more rapidly than the dissociation rate and the formation of intermediate subcapped metallo‐cylinders are too transient (or at too low concentration) to be detected. Control experiments in the absence of NQO1 and NADPH (Figure S27) showed no formation of [Ni_2_(L1)_3_]^4+^ or subcapped cylinders, except the small amounts of 5‐capped cylinder also seen with NQO1 which we ascribe to a limited amount of electrochemical quinone reduction in the ESI process giving rise to loss of one QPA, and also explaining why only this and not other subcapped species are observed in the NQO1 experiment.

**Figure 2:**
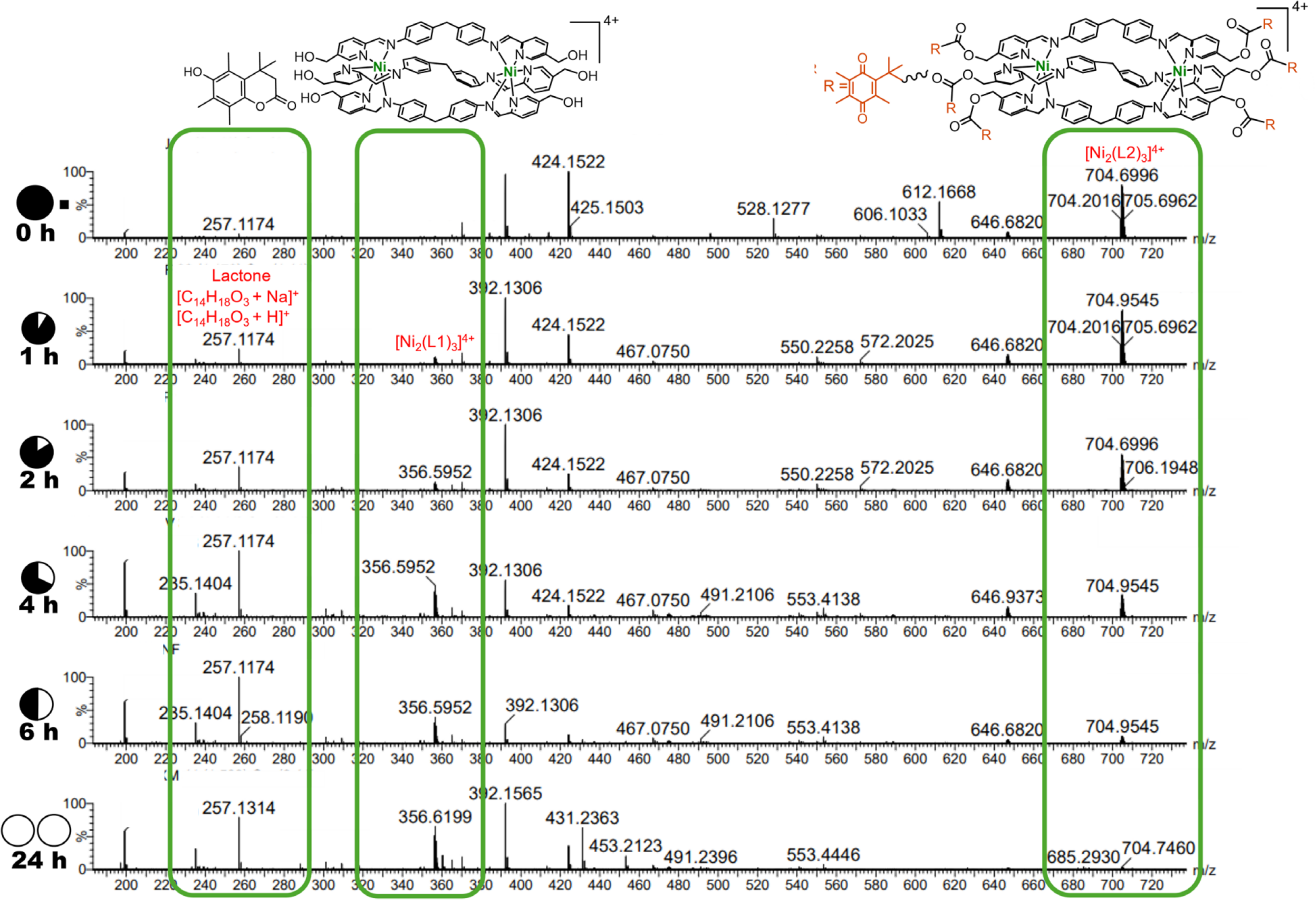
Decapping of [Ni_2_(L2)_3_]Cl_4_, by NQO1 (20 µg mL^−1^) and NADPH (8 equiv) in water over time monitored by ESI‐MS at 37 °C. *m*/*z*_704_ = [Ni_2_(L2)_3_]^4+^; *m*/*z*_356_ = [Ni_2_(L1)_3_]^4+^; *m*/*z*_257_ = [C_14_H_18_O_3_ + Na]^+^, lactone; and *m*/*z*_235_ = [C_14_H_18_O_3_ + H]^+^, lactone.

To explore the DNA junction binding of the metallo‐cylinders, we deployed a polyacrylamide gel electrophoresis assay using three complementary 14‐mer nucleotide strands (S1, S2, and S3 – See Supporting Information). At room temperature the 3WJ formation is entropically disfavored for these short oligomers and so no 3WJ band is observed (only the single strands; Figure 3 lane 1). In the presence of a 3WJ binder, such as the unfunctionalized cylinder, the 3WJ is stabilized and the 3WJ band is observed in the gel (Figure 3 lane 3; positive control).^[^ ^51^, ^90^, ^91^ ^]^

**Figure 3:**
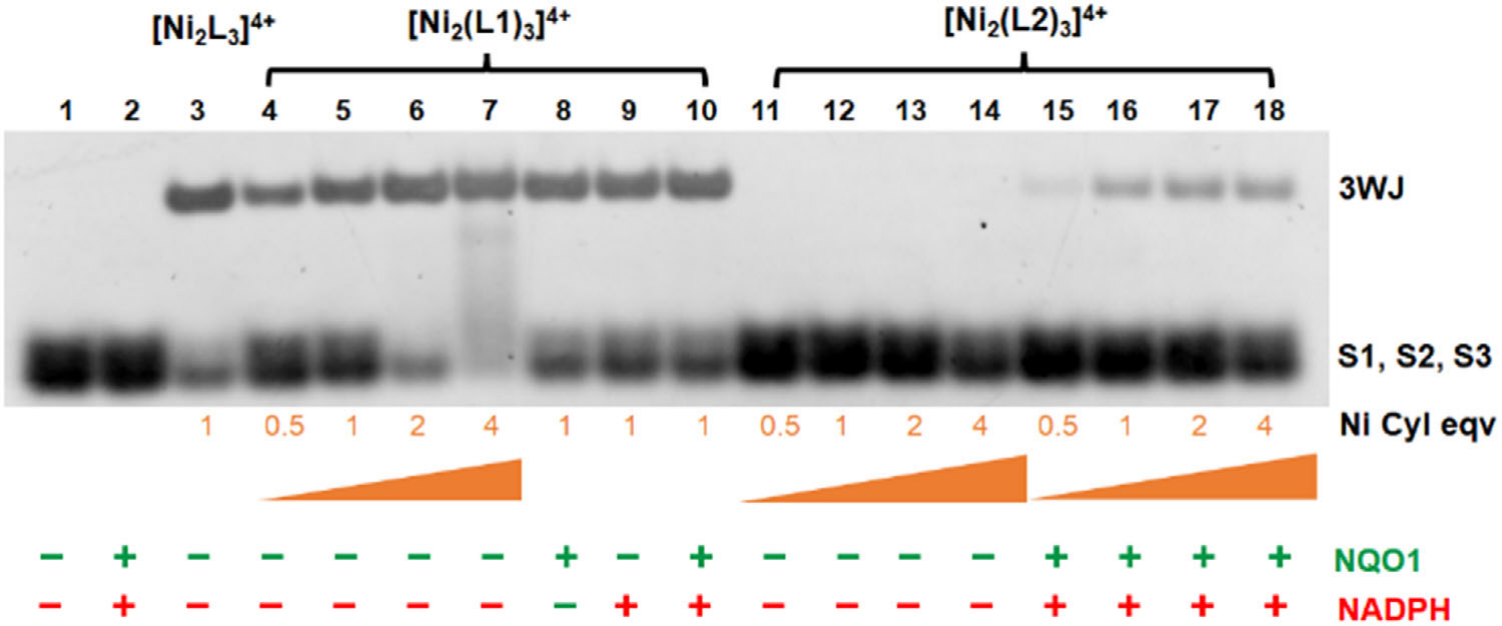
PAGE gel showing the 3WJ binding of uncapped Ni cylinder, [Ni_2_(L1)_3_]^4+^ (lanes 4–10). Capped cylinder, [Ni_2_(L2)_3_]^4+^, showing no 3WJ binding (lanes 11–14) in absence, and 3WJ binding in presence of NQO1/NADPH (lanes 15 – 18). Lanes 1–2 are negative control (untreated) and lane 3 is a positive control (Ni parent cylinder, [Ni_2_L_3_]Cl_4_). 14‐mer DNA oligonucleotide single strands used were S1: 5′‐CGGAACGGCACTCG‐3′, S2: 5′‐CGAGTGCAGCGTGG‐3′, and S3: 5′‐CCACGCTCGTTCCG‐3′ and the gel was stained by SYBR™ Gold Nucleic Acid Gel Stain. NQO1 concentration in the sample mixture was 50 µg mL^−1^ and NADPH was used at a 100‐fold molar equivalent with respect to the concentration of metallo‐cylinder. The intensity of the 3WJ band is lower than expected if all complex is decapped in this experiment, because the L2 complex shows some instability in presence of free NADPH (Figure S42; in cellulo all NADPH is believed to be protein‐bound) and there will be some complex degradation before binding enzyme and decapping.

Adding the uncapped [Ni_2_(L1)_3_]^4+^ cylinder, we observed stabilization of the 3WJ (Figure 3 lanes 4–7) in a concentration dependent manner as would be expected. By contrast, with the QPA capped cylinder [Ni_2_(L2)_3_]^4+^ no 3WJ band is observed even at high loading (Figure 3 lanes 11–14). To confirm that the quinone is not quenching the fluorescent stain and masking a 3WJ band, we repeated the experiment with a longer 3WJ that is stable at room temperature (Figure S28); again [Ni_2_(L1)_3_]^4+^ was observed to bind (evidenced by a gel shift) while [Ni_2_(L2)_3_]^4+^ did not (no gel shift and no effect on staining). To ensure the free lactone (Scheme 1) does not interfere with the gel, we reacted 6 equivs (with respect to cylinder) of ethyl ester of QPA (QPA Et, which can only release a lactone and an ethoxy group upon action of the NQO1) with DNA 3WJ and respective cylinders. No quenching of or interference with the 3WJ band was observed (Figure S29). Thus, the six QPA caps are sufficient to prevent 3WJ binding, but when removed will release an agent that can bind.

To confirm this, when [Ni_2_(L2)_3_]^4+^ is added in the presence of NQO1 (50 µg mL^−1^) and NADPH (100 equivs), a concentration‐dependent 3WJ band emerges in the PAGE experiment (Figure 3 lanes 15–18). The band also increases in intensity with increasing incubation times (up to 10 h; Figures S30–S32).

To further confirm that it is the enzyme action that activates the 3WJ binding, we used a known inhibitor of NQO1, dicoumarol that blocks the NADPH binding site of NQO1,^[^ ^92^ ^]^ and therefore inhibits the quinone reduction. In the presence of dicoumarol (50 µM), 3WJ recognition by the capped metallo‐cylinder remains switched off (Figure 4 lanes 15–18). Experiments using NADPH in the absence of NQO1 similarly did not lead to 3WJ binding (Figure S33 lanes 15–18,); together these confirm the role of the NQO1 enzyme in the activation of the DNA junction binding.

**Figure 4:**
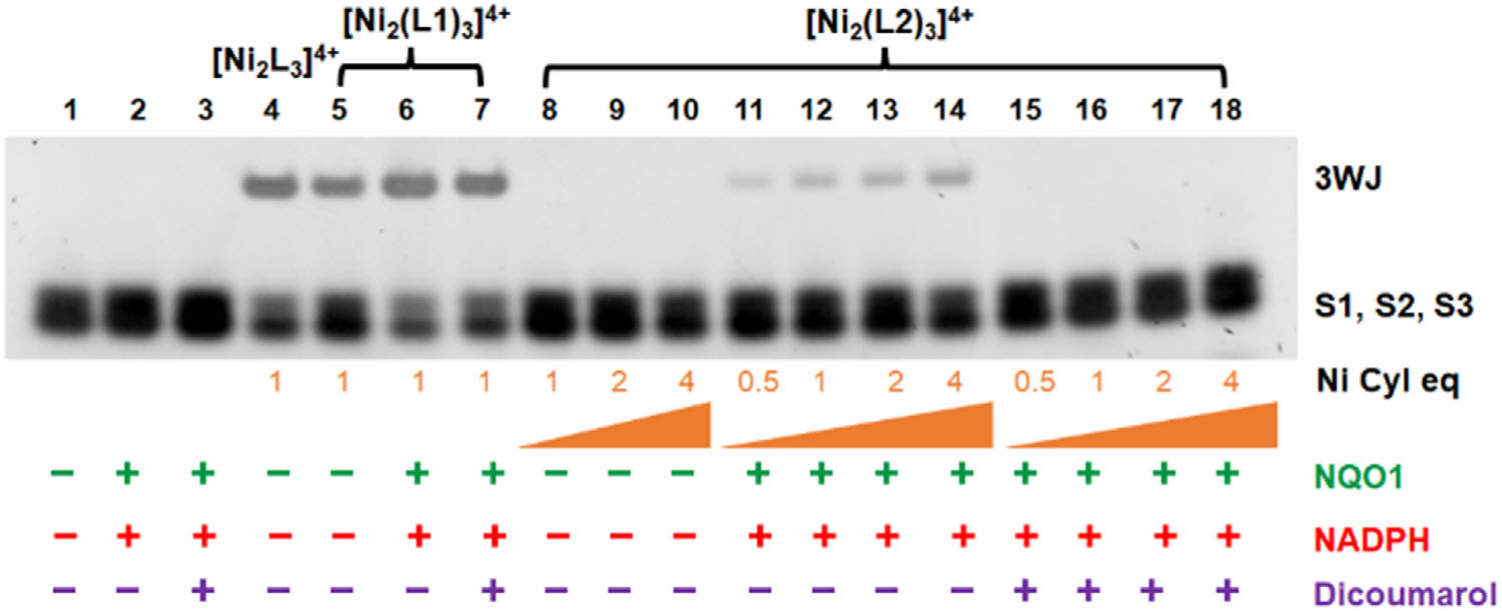
PAGE gel demonstrating the inhibition of NQO1 by dicoumarol preventing the QPA release and 3WJ binding of capped cylinder (lanes 15–18).

We also conducted DNA melting experiments to assess the 3WJ binding affinity of both the capped and uncapped cylinders. There is no significant change in melting temperature (*T*_m_) of DNA 3WJ (39.7 ± 0.5 °C) in presence of the capped cylinder [Ni_2_(L2)_3_]^4+^ (40.2 ± 0.3 °C) but a dramatic increase in presence of [Ni_2_(L1)_3_]^4+^ (58.1 ± 1.0 °C) confirming the binding of the L1 cylinder but not the capped L2 cylinder to the DNA 3WJ (Figure S34). Similarly gels and melting experiments show the L1 cylinder but not the capped L2 cylinder binds RNA 3WJs (Figures S35–S36).

Attaching QPA to a DNA‐binding molecule does not per se prevent it from DNA binding – the DNA intercalator doxorubicin retains its DNA‐binding when a single QPA is attached.^[^ ^93^ ^]^ To gain better molecular insight into how these six QPA groups on the cylinder might interfere with the binding, we explored simulations. We generated computational (DFT) models of the new [Ni_2_(L2)_3_]^4+^ cylinder using the crystal structure of the unsubstituted parent cylinder as a starting point and parameterizing using an adapted approach as previously described for unsubstituted cylinders.^[^ ^94^ ^]^ We then subjected the cylinder to molecular dynamics (MD) simulations, which showed that the methylene attachment allows the QPA groups to explore the whole space around the cylinder, not only stretched out away from the ends of the cylinders but also folded back across the cylinder and interacting with the spacer units (Figure 5). With six such arms exploring the 3D space, at least one arm is frequently folded back into the space around the centre of the cylinder (Figure S37A).

**Figure 5:**
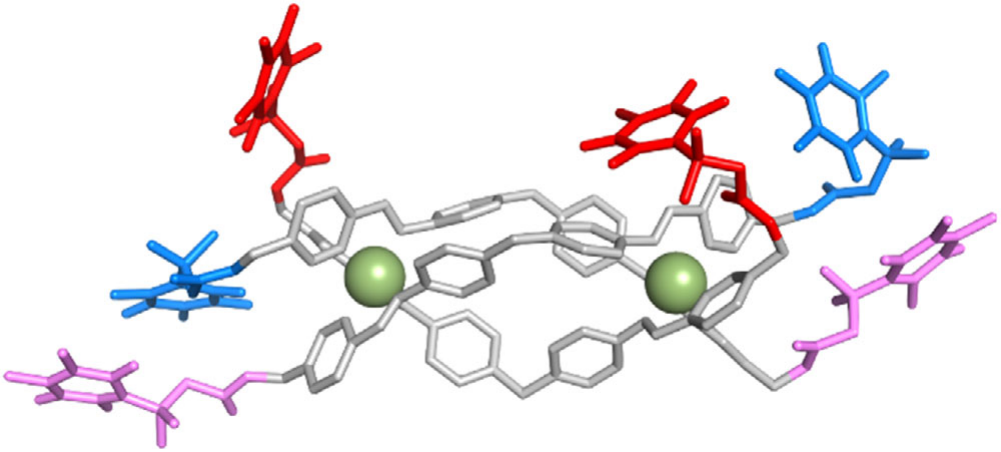
Molecular dynamics snapshot of the capped cylinder alone (in explicit water and 50 mM NaCl, omitted for clarity). The CH_2_ linker allows the QPA caps to explore all the space around the cylinder including extended out along the axis of the cylinder (violet), folding back and covering the rigid core of the cylinder (red) or other intermediate positions (blue). The distance of any QPA cap relative to any central CH_2_ spacer was calculated over the course of each simulation and can be seen in Figure S37.

We next conducted MD simulations where the cylinder was placed directly into the centre of a DNA 3WJ, starting with the QPA arms positioned outside the 3WJ cavity. The cylinder does remain in the 3WJ in the timescale of the simulations (3 simulations, 1 µs each), but the arms were considerably more conformationally restricted in their movement (Figures S37B, S38, and S39). Thus, it seems that the six QPA groups do not sterically prevent DNA junction binding (as a cucurbituril placed around the centre of the cylinder does^[^ ^51^ ^]^) but rather the effect is more subtle and may arise from the entropic loss that the conformational restriction brings. We considered that in a large DNA structure, the arms could potentially also interfere with entry into the cavity, though we discount such a kinetic effect here for the small and unstable 3WJs used in our PAGE experiments. Using and removing conformational entropy loss to disfavor and then favor binding would represent a new concept for switching nucleic acid binding activity. Although this concept of conformational entropy modifying behavior is a new approach in NA binding, related entropic effects are described in polypeptides where intrinsically disordered proteins may become better ordered under molecular crowding conditions inside the cell when the volume of space for their entopic disorder is restricted.^[^ ^95^ ^]^

The lack of 3WJ binding contrasts with the effective 3WJ binding behavior we previously observed when six arginine groups were attached to a cylinder at the 5‐pyridine positions.^[^ ^96^ ^]^ In that study the arginines were connected by an amide bond attached to the pyridine ring, and thus have only restricted conformational flexibility, whereas herein the methylene unit that connects the cylinder and the ester allows the QPA unit to fold back toward the cylinder increasing the conformational space it can access.

To explore NQO1 activity on the capped cylinder inside cells, we treated NQO1 overexpressing lung cancer cell line (A549) with [Ni_2_(L2)_3_]^4+^ in the presence and absence of NQO1 inhibitor dicoumarol and determined the antiproliferative activity using an MTT assay. [Ni_2_(L2)_3_]Cl_4_ entered cells (Figure S41) and displayed an IC_50_ of 3 µM when added alone (Figure 6 and Table S1). A ∼5‐fold decrease in activity (IC_50_ 14.9 µM) was observed when [Ni_2_(L2)_3_]Cl_4_ was coadministered with 25 µM of dicoumarol with a further loss of activity when the dicoumarol was increased to 50 µM (Figure 6 and Table S1). These results are consistent with NQO1 action being required to activate the capped cylinder inside the cell, as it is in the gel 3WJ‐binding assays.

**Figure 6:**
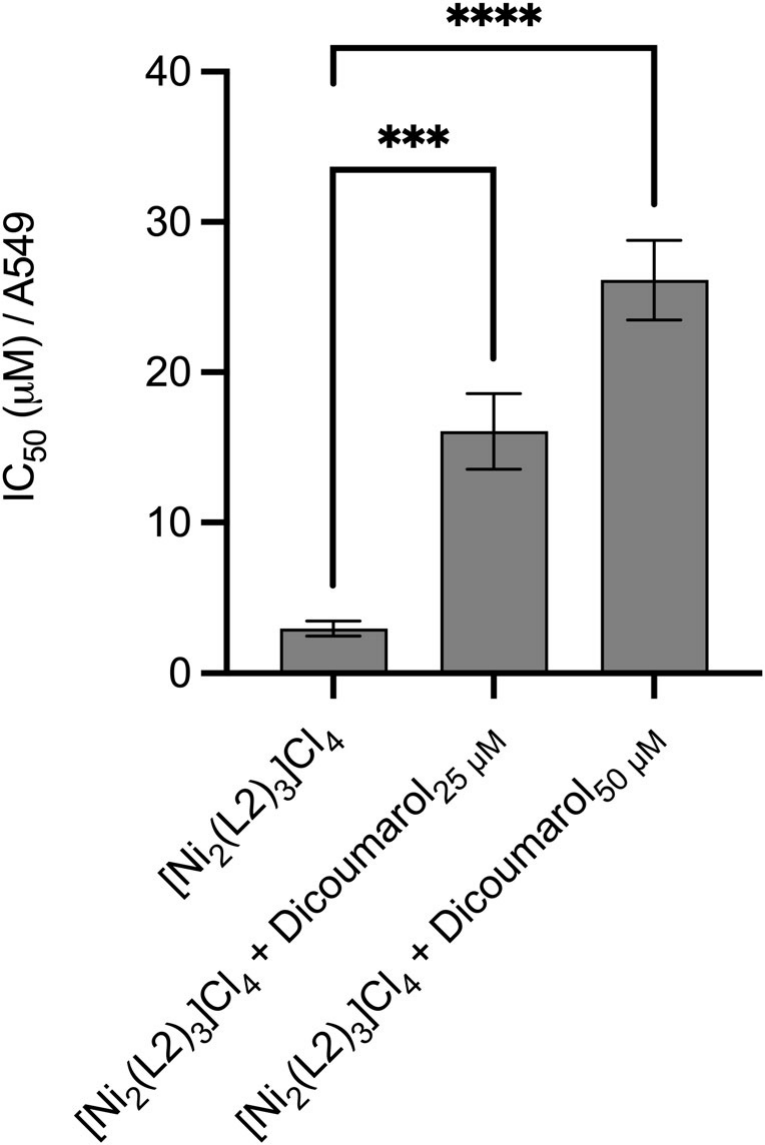
Graph shows the mean IC_50_ values for [Ni_2_(L2)_3_]Cl_4_ alone, and in presence of 25 µM of dicoumarol and 50 µM of dicoumarol ± SD (error bars are standard deviations) respectively; *** and **** statistically significantly different from [Ni_2_(L2)_3_]Cl_4_ as assessed by a one‐way ANOVA followed by a Dunnett's post hoc *t*‐test *p* < 0.001 and 0.0001 respectively (*n* = 3).

In summary, we have demonstrated how we can create a large, inert, metallo‐supramolecular structure which is then acted upon by an enzyme and transformed into an active, but still metallo‐supramolecular, structure whose surfaces then perform a remarkable (non‐covalent) assembly of DNA into a different DNA super‐structure. This concept of enzyme action on a supramolecular assembly to create function, is one with potential to be applied broadly to other systems. Leigh, Papot and coworkers have previously created a rotaxane that was modified by a glycosidase,^[^ ^97^ ^]^ but in that case the role of the enzyme was to break apart the supramolecular assembly into its components, one of which had an action. Here the approach is different – to transform one supramolecule into another supramolecule which has an activity. Although unusual DNA‐binding is the focus in this example, it has potential to be applied to other sorts of activity.

The particular activity herein is an exciting one though: DNA junction structures are key and active biological structures that are drug targets for cancer, viruses (where the same motif can also be seen in viral RNA^[^ ^98^, ^99^, ^100^ ^]^) and neuro‐diseases, as well as key construction motifs in DNA nanotechnology. To gain a level of “on command” control over the junction binding or junction assembly or modulation of junction behavior offers a key new capability for DNA recognition and nanoscience. Achieving this using a switch that involves a human enzyme overexpressed in many disease states, together with its biological cofactor means that this example represents an AND logic switch; the inhibition by dicoumarol extends that to a NAND (AND followed by NOT) switch. The versatility of enzymes portends the possibility to engineer other sophisticated logic switch combinations.

Both the switching concept we elaborate and the specific approach we pioneer to achieve this not only has potential for wide application across supramolecular chemistry, but also provides a new roadmap for how one might control and modulate a non‐canonical DNA junction structure within a biological system. We are currently refining and optimizing our design concept to meet that challenge.

## Supporting Information

The authors have cited additional references within the Supporting Information.^[^ ^101^, ^102^, ^103^, ^104^, ^105^, ^106^, ^107^, ^108^, ^109^, ^110^, ^111^, ^112^, ^113^, ^114^, ^115^, ^116^, ^117^ ^]^

## Author Contributions

M.J.H and S.K. conceived the project and designed experiments. M.J.H supervised and directed the project and N.J.H supervised cell studies. S.K. undertook synthesis, gel, spectroscopic experiments and cell studies. S.J.D undertook the MD simulations and UV melting experiments. C.A.J.H. undertook some of the synthesis. S.K. and M.J.H. drafted the manuscript which all authors commented on.

## Conflict of Interests

The authors declare no conflict of interest.

## Supporting information



Supporting Information

## Data Availability

The data that support the findings of this study are openly available in UBIRA at https://doi.org/10.25500/edata.bham.00001250, reference number 1250.

## References

[1] R. K. Singh , S. Kumar , D. N. Prasad , T. R. Bhardwaj , Eur. J. Med. Chem. 2018, 151, 401–433. 29649739 10.1016/j.ejmech.2018.04.001

[2] Y. Li , J. Med. Chem. 2024, 67, 5113–5143. 38552031 10.1021/acs.jmedchem.3c02476

[3] D. Gibson , Dalton Trans. 2009, 10681–10689. 20023895 10.1039/b918871c

[4] X. Wang , Z. Guo , Chem. Soc. Rev. 2013, 42, 202–224. 23042411 10.1039/c2cs35259a

[5] R. G. Kenny , C. J. Marmion , Chem. Rev. 2019, 119, 1058–1137. 30640441 10.1021/acs.chemrev.8b00271

[6] S. Alassadi , M. J. Pisani , N. J. Wheate , Dalton Trans. 2022, 51, 10835–10846. 35781551 10.1039/d2dt01875f

[7] M. B. Martins‐Teixeira , I. Carvalho , ChemMedChem 2020, 15, 933–948. 32314528 10.1002/cmdc.202000131

[8] S. Venugopal , V. Sharma , A. Mehra , I. Singh , G. Singh , Chem. Biol. Drug Des. 2022, 100, 580–598. 35822451 10.1111/cbdd.14116

[9] A. Rahman , P. O'Sullivan , I. Rozas , MedChemComm 2019, 10, 26–40. 30774852 10.1039/c8md00425k PMC6349057

[10] H. Y. Alniss , J. Med. Chem. 2019, 62, 385–402. 30059627 10.1021/acs.jmedchem.8b00233

[11] M. J. Hannon , Pure Appl. Chem. 2007, 79, 2243–2261.

[12] E. Ivens , M. M. D. Cominetti , M. Searcey , Bioorg. SMed. Chem. 2022, 69, 116897. 10.1016/j.bmc.2022.116897 35764032

[13] K. T. McQuaid , A. Pipier , C. J. Cardin , D. Monchaud , Nucleic Acids Res. 2022, 50, 12636–12656. 36382400 10.1093/nar/gkac1043 PMC9825177

[14] S. Neidle , Nat. Rev. Chem. 2017, 1, 0041.

[15] J. Spiegel , S. Adhikari , S. Balasubramanian , Trends Chem 2020, 2, 123–136. 32923997 10.1016/j.trechm.2019.07.002 PMC7472594

[16] M. J. Hannon , Chem. Soc. Rev. 2007, 36, 280–295. 17264930 10.1039/b606046n

[17] S. L. Brown , S. Kendrick , Pharmaceuticals 2021, 14, 96.33513764

[18] A. Jain , G. Wang , K. M. Vasquez , Biochimie 2008, 90, 1117–1130. 18331847 10.1016/j.biochi.2008.02.011 PMC2586808

[19] M. Dalla Pozza , A. Abdullrahman , C. J. Cardin , G. Gasser , J. P. Hall , Chem. Sci. 2022, 13, 10193–10215. 36277639 10.1039/d2sc01793h PMC9473520

[20] A. Bansal , S. Kaushik , S. Kukreti , F.Genet., 2022, 13, 959258. 10.3389/fgene.2022.959258 PMC9483843 36134025

[21] Q. Song , Y. Hu , A. Yin , H. Wang , Q. Yin , Int. J. Mol. Sci. 2022, 23, 9730. 36077130 10.3390/ijms23179730 PMC9456528

[22] V. Kocman , J. Plavec , Nat. Commun. 2017, 8, 15355. 28513602 10.1038/ncomms15355 PMC5442326

[23] M. Martella , F. Pichiorri , R. V. Chikhale , M. A. S. Abdelhamid , Z. A. E. Waller , S. S. Smith , Nucleic Acids Res. 2022, 50, 3445–3455. 35253884 10.1093/nar/gkac158 PMC8989526

[24] J. J. King , K. L. Irving , C. W. Evans , R. V. Chikhale , R. Becker , C. J. Morris , C. D. Peña Martinez , P. Schofield , D. Christ , L. H. Hurley , Z. A. E. Waller , K. S. Iyer , N. M. Smith , J. Am. Chem. Soc. 2020, 142, 20600–20604. 33253551 10.1021/jacs.0c11708

[25] L. Cardo , M. J. Hannon , in Metallo‐Drugs: Development and Action of Anticancer Agents, (Eds.: A. Sigel , H. Sigel , E. Freisinger , R. K. O. Sigel ), Metal Ions Life Sciences, Vol. 18, De Gruyter, Berlin, Germany 2018, pp. 303–324.

[26] H. Tateishi‐Karimata , N. Sugimoto , Chem. Commun. 2020, 56, 2379–2390. 10.1039/c9cc09771f 32022004

[27] H. Tateishi‐Karimata , N. Sugimoto , Nucleic Acids Res. 2021, 49, 7839–7855. 34244785 10.1093/nar/gkab580 PMC8373145

[28] K. D. Makova , M. H. Weissensteiner , Trends Genet. 2023, 39, 109–124. 36604282 10.1016/j.tig.2022.11.005 PMC9877202

[29] C. Ducani , A. Leczkowska , N. J. Hodges , M. J. Hannon , Angew. Chem. Int. Ed. 2010, 49, 8942–8945. 10.1002/anie.201004471 20936610

[30] K. Duskova , P. Lejault , É. Benchimol , R. Guillot , S. Britton , A. Granzhan , D. Monchaud , J. Am. Chem. Soc. 2020, 142, 424–435. 31833764 10.1021/jacs.9b11150

[31] J. Malina , H. Kostrhunova , P. Scott , V. Brabec , Nucleic Acids Res. 2023, 51, 7174–7183. 37351627 10.1093/nar/gkad536 PMC10415117

[32] J. Zell , F. Rota Sperti , S. Britton , D. Monchaud , RSC Chem. Biol. 2021, 2, 47–76. 35340894 10.1039/d0cb00151a PMC8885165

[33] A. C. G. Hotze , B. M. Kariuki , M. J. Hannon , Angew. Chem. Int. Ed. 2006, 45, 4839–4842. 10.1002/anie.200601351 16802394

[34] A. C. G. Hotze , N. J. Hodges , R. E. Hayden , C. Sanchez‐Cano , C. Paines , N. Male , M.‐K. Tse , C. M. Bunce , J. K. Chipman , M. J. Hannon , Chem. Biol. 2008, 15, 1258–1267. 19101470 10.1016/j.chembiol.2008.10.016

[35] A. J. Pope , C. Bruce , B. Kysela , M. J. Hannon , Dalton Trans. 2010, 39, 2772–2774. 20200702 10.1039/b927129p

[36] A. Oleksi , A. G. Blanco , R. Boer , I. Usón , J. Aymamí , A. Rodger , M. J. Hannon , M. Coll , Angew. Chem. Int. Ed. 2006, 45, 1227–1231. 10.1002/anie.200503822 16463312

[37] A. Oleksy , A. G. Blanco , R. Boer , I. Usón , J. Aymamí , A. Rodger , M. J. Hannon , M. Coll , Angew. Chem. Int. Ed. 2006, 45, 1834–1834. 10.1002/anie.200503822 16463312

[38] J. M. C. A. Kerckhoffs , J. C. Peberdy , I. Meistermann , L. J. Childs , C. J. Isaac , C. R. Pearmund , V. Reudegger , S. Khalid , N. W. Alcock , M. J. Hannon , A. Rodger , Dalton Trans. 2007, 734–742. 10.1039/b614093a 17279244

[39] M. J. Hannon , C. L. Painting , A. Jackson , J. Hamblin , W. Errington , Chem. Commun. 1997, 1807–1808.

[40] L. Cerasino , M. J. Hannon , E. Sletten , Inorg. Chem. 2007, 46, 6245–6251. 17407284 10.1021/ic062415c

[41] K. Duskova , J. Lamarche , S. Amor , C. Caron , N. Queyriaux , M. Gaschard , M.‐J. Penouilh , G. de Robillard , D. Delmas , C. H. Devillers , A. Granzhan , M.‐P. Teulade‐Fichou , M. Chavarot‐Kerlidou , B. Therrien , S. Britton , D. Monchaud , J. Med. Chem. 2019, 62, 4456–4466. 30942581 10.1021/acs.jmedchem.8b01978

[42] J. Zell , K. Duskova , L. Chouh , M. Bossaert , N. Chéron , A. Granzhan , S. Britton , D. Monchaud , Nucleic Acids Res. 2021, 49, 10275–10288. 34551430 10.1093/nar/gkab796 PMC8501980

[43] A. Pipier , T. Chetot , A. Kalamatianou , N. Martin , M. Caroff , S. Britton , N. Chéron , L. Trantírek , A. Granzhan , D. Monchaud , Angew. Chem. Int. Ed. 2024, 63, e202409780. 10.1002/anie.202409780 38873877

[44] J. Gómez‐González , Y. Pérez , G. Sciortino , L. Roldan‐Martín , J. Martínez‐Costas , J.‐D. Maréchal , I. Alfonso , M. Vázquez López , M. E. Vázquez , Angew. Chem. Int. Ed. 2021, 60, 8859–8866. 10.1002/anie.202013039 PMC8016737 33290612

[45] A. Alcalde‐Ordóñez , N. Barreiro‐Piñeiro , B. McGorman , J. Gómez‐González , D. Bouzada , F. Rivadulla , M. E. Vázquez , A. Kellett , J. Martínez‐Costas , M. V. López , Chem. Sci. 2023, 14, 14082–14091. 38098723 10.1039/d3sc03303a PMC10718067

[46] B. McGorman , S. Poole , M. V. López , A. Kellett , Methods 2023, 219, 30–38. 37690737 10.1016/j.ymeth.2023.09.002

[47] H. Song , N. J. Rogers , S. J. Allison , V. Brabec , H. Bridgewater , H. Kostrhunova , L. Markova , R. M. Phillips , E. C. Pinder , S. L. Shepherd , L. S. Young , J. Zajac , P. Scott , Chem. Sci. 2019, 10, 8547–8557. 31803429 10.1039/c9sc02651g PMC6839601

[48] D. H. Simpson , A. Hapeshi , N. J. Rogers , V. Brabec , G. J. Clarkson , D. J. Fox , O. Hrabina , G. L. Kay , A. K. King , J. Malina , A. D. Millard , J. Moat , D. I. Roper , H. Song , N. R. Waterfield , P. Scott , Chem. Sci. 2019, 10, 9708–9720. 32015803 10.1039/c9sc03532j PMC6977464

[49] A. C. Pearcy , J. D. Crowley , Chem. – Eur. J. 2023, 29, e202203752. 36683008 10.1002/chem.202203752

[50] J. Malina , J. D. Crowley , V. Brabec , Chem.‐Biol. Interact. 2024, 395, 111031. 38703805 10.1016/j.cbi.2024.111031

[51] C. A. J. Hooper , L. Cardo , J. S. Craig , L. Melidis , A. Garai , R. T. Egan , V. Sadovnikova , F. Burkert , L. Male , N. J. Hodges , D. F. Browning , R. Rosas , F. Liu , F. V. Rocha , M. A. Lima , S. Liu , D. Bardelang , M. J. Hannon , J. Am. Chem. Soc. 2020, 142, 20651–20660. 33215921 10.1021/jacs.0c07750

[52] E. A. Bey , M. S. Bentle , K. E. Reinicke , Y. Dong , C.‐R. Yang , L. Girard , J. D. Minna , W. G. Bornmann , J. Gao , D. A. Boothman , Proc. Natl. Acad. Sci. USA 2007, 104, 11832–11837. 17609380 10.1073/pnas.0702176104 PMC1913860

[53] X. Cui , T. Jin , X. Wang , G. Jin , Z. Li , L. Lin , Oncol. Rep. 2014, 32, 2589–2595. 25231218 10.3892/or.2014.3494

[54] Z. Li , Y. Zhang , T. Jin , J. Men , Z. Lin , P. Qi , Y. Piao , G. Yan , BMC Cancer 2015, 15, 207. 25880877 10.1186/s12885-015-1227-8 PMC4396547

[55] A. Marín , A. López de Cerain , E. Hamilton , A. D. Lewis , J. M. Martinez‐Peñuela , M. A. Idoate , J. Bello , Br. J. Cancer 1997, 76, 923–929. 9328153 10.1038/bjc.1997.485 PMC2228079

[56] M. S. Bentle , K. E. Reinicke , Y. Dong , E. A. Bey , D. A. Boothman , Cancer Res. 2007, 67, 6936–6945. 17638905 10.1158/0008-5472.CAN-07-0935

[57] Y. Yang , Y. Zhang , Q. Wu , X. Cui , Z. Lin , S. Liu , L. Chen , J. Exp. Clin. Cancer Res. 2014, 33, 14. 24499631 10.1186/1756-9966-33-14 PMC3944477

[58] C. Glorieux , J. M. Sandoval , N. Dejeans , G. Ameye , H. A. Poirel , J. Verrax , P. B. Calderon , Life Sci. 2016, 145, 57–65. 26687450 10.1016/j.lfs.2015.12.017

[59] M. Ough , A. Lewis , E. A. Bey , J. Gao , J. M. Ritchie , W. Bornmann , D. A. Boothman , L. W. Oberley , J. J. Cullen , Cancer Biol. Ther. 2005, 4, 102–109. 10.4161/cbt.4.1.1382 15662131

[60] A. M. Lewis , M. Ough , M. M. Hinkhouse , M.‐S. Tsao , L. W. Oberley , J. J. Cullen , Mol. Carcinog. 2005, 43, 215–224. 16003741 10.1002/mc.20107 PMC7262682

[61] N. S. Awadallah , D. Dehn , R. J. Shah , S. R. Nash , Y. K. Chen , D. Ross , J. S. Bentz , K. R. Shroyer , Appl. Immunohistochem. Mol. Morphol. 2008, 16, 24–31. 18091324 10.1097/PAI.0b013e31802e91d0

[62] D. Siegel , D. Ross , N. W. Gibson , P. C. Preusch , Cancer Res. 1990, 50, 7483–7489. 1701346

[63] K. Mikami , M. Naito , T. Ishiguro , H. Yano , A. Tomida , T. Yamada , N. Tanaka , T. Shirakusa , T. Tsuruo , Japan. J. Cancer Res. 1998, 89, 910–915. 9818026 10.1111/j.1349-7006.1998.tb00648.x PMC5921949

[64] D. Siegel , D. Ross , Free Radical Biol. Med. 2000, 29, 246–253. 11035253 10.1016/s0891-5849(00)00310-5

[65] L. Lin , Y. Qin , T. Jin , S. Liu , S. Zhang , X. Shen , Z. Lin , Exp. Mol. Pathol. 2014, 96, 200–205. 24384455 10.1016/j.yexmp.2013.12.008

[66] X. Cui , L. Li , G. Yan , K. Meng , Z. Lin , Y. Nan , G. Jin , C. Li , BMC Cancer 2015, 15, 244. 25885439 10.1186/s12885-015-1271-4 PMC4399114

[67] D. Thapa , P. Meng , R. G. Bedolla , R. L. Reddick , A. P. Kumar , R. Ghosh , Cancer Res. 2014, 74, 5644–5655. 25125658 10.1158/0008-5472.CAN-14-0562 PMC4184940

[68] Y. Ma , J. Kong , G. Yan , X. Ren , D. Jin , T. Jin , L. Lin , Z. Lin , BMC Cancer 2014, 14, 414. 24912939 10.1186/1471-2407-14-414 PMC4058702

[69] M. F. Mendoza , N. M. Hollabaugh , S. U. Hettiarachchi , R. L. McCarley , Biochemistry 2012, 51, 8014–8026. 22989153 10.1021/bi300760u PMC3525107

[70] K. Zhang , D. Chen , K. Ma , X. Wu , H. Hao , S. Jiang , J. Med. Chem. 2018, 61, 6983–7003. 29712428 10.1021/acs.jmedchem.8b00124

[71] J. Chang , W. Cai , C. Liang , Q. Tang , X. Chen , Y. Jiang , L. Mao , M. Wang , J. Am. Chem. Soc. 2019, 141, 18136–18141. 31589435 10.1021/jacs.9b08669

[72] X. Zhang , X. Li , Z. Li , X. Wu , Y. Wu , Q. You , X. Zhang , Org. Lett. 2018, 20, 3635–3638. 29847952 10.1021/acs.orglett.8b01409

[73] L. Fang , X. Qin , J. Zhao , S. Gou , Inorg. Chem. 2019, 58, 2191–2200. 30657321 10.1021/acs.inorgchem.8b03386

[74] M. Li , G. Zhao , W.‐K. Su , Q. Shuai , Front. Chem. 2020, 8, 647. 32850662 10.3389/fchem.2020.00647 PMC7406800

[75] X. Yang , J. Duan , L. Wu , Future Med. Chem. 2022, 14, 363–383. 35102756 10.4155/fmc-2021-0289

[76] S. R. Gayam , P. Venkatesan , Y.‐M. Sung , S.‐Y. Sung , S.‐H. Hu , H.‐Y. Hsu , S.‐P. Wu , Nanoscale 2016, 8, 12307–12317. 27271875 10.1039/c6nr03525f

[77] J. Xie , S. Tian , H. Zhang , C. Feng , Y. Han , H. Dai , L. Yan , Biomacromolecules 2023, 24, 2225–2236. 37040694 10.1021/acs.biomac.3c00134

[78] W. C. Silvers , B. Prasai , D. H. Burk , M. L. Brown , R. L. McCarley , J. Am. Chem. Soc. 2013, 135, 309–314. 23198810 10.1021/ja309346f PMC4703112

[79] S. U. Hettiarachchi , B. Prasai , R. L. McCarley , J. Am. Chem. Soc. 2014, 136, 7575–7578. 24813575 10.1021/ja5030707 PMC4046754

[80] W. S. Shin , M.‐G. Lee , P. Verwilst , J. H. Lee , S.‐G. Chi , J. S. Kim , Chem. Sci. 2016, 7, 6050–6059. 30034745 10.1039/c6sc02236g PMC6022148

[81] W. S. Shin , J. Han , P. Verwilst , R. Kumar , J.‐H. Kim , J. S. Kim , Bioconjugate Chem. 2016, 27, 1419–1426. 10.1021/acs.bioconjchem.6b00184 27135737

[82] N. Gao , W. Yang , H. Nie , Y. Gong , J. Jing , L. Gao , X. Zhang , Biosens. Bioelectron. 2017, 96, 300–307. 28511113 10.1016/j.bios.2017.05.019

[83] Q. Gong , F. Yang , J. Hu , T. Li , P. Wang , X. Li , X. Zhang , Eur. J. Med. Chem. 2021, 224, 113707. 34303080 10.1016/j.ejmech.2021.113707

[84] B. Peng , G. Chen , Y. Li , H. Zhang , J. Shen , J.‐T. Hou , Z. Li , Anal. Chem. 2022, 94, 11159–11167. 35916489 10.1021/acs.analchem.2c01189

[85] Q. Pei , S. Lu , J. Zhou , B. Jiang , C. Li , Z. Xie , X. Jing , ACS Appl. Mater. Interfaces 2021, 13, 59708–59719. 34879654 10.1021/acsami.1c19058

[86] S. Milstien , L. A. Cohen , J. Am. Chem. Soc. 1972, 94, 9158–9165. 4642365 10.1021/ja00781a029

[87] M. E. Jung , G. Piizzi , Chem. Rev. 2005, 105, 1735–1766. 15884788 10.1021/cr940337h

[88] A. Lavalette , J. Hamblin , A. Marsh , D. M. Haddleton , M. J. Hannon , Chem. Commun. 2002, 3040–3041. 10.1039/b210019c 12536806

[89] G. I. Pascu , A. C. G. Hotze , C. Sanchez‐Cano , B. M. Kariuki , M. J. Hannon , Angew. Chem. Int. Ed. 2007, 46, 4374–4378. 10.1002/anie.200700656 17477461

[90] J. Malina , M. J. Hannon , V. Brabec , Chem. – Eur. J. 2007, 13, 3871–3877. 17397023 10.1002/chem.200700159

[91] J. S. Craig , L. Melidis , H. D. Williams , S. J. Dettmer , A. A. Heidecker , P. J. Altmann , S. Guan , C. Campbell , D. F. Browning , R. K. O. Sigel , S. Johannsen , R. T. Egan , B. Aikman , A. Casini , A. Pöthig , M. J. Hannon , J. Am. Chem. Soc. 2023, 145, 13570–13580. 37318835 10.1021/jacs.3c00118 PMC10311459

[92] G. Asher , O. Dym , P. Tsvetkov , J. Adler , Y. Shaul , Biochemistry 2006, 45, 6372–6378. 16700548 10.1021/bi0600087

[93] Z. He , W. Xiang , Q. Fan , L. Wang , J. Chao , Chem. Commun. 2023, 59, 912–915. 10.1039/d2cc06367k 36594872

[94] L. Melidis , I. B. Styles , M. J. Hannon , Chem. Sci. 2021, 12, 7174–7184. 34123344 10.1039/d1sc00933h PMC8153246

[95] D. Rajendran , S. Mitra , H. Oikawa , K. Madhurima , A. Sekhar , S. Takahashi , A. N. Naganathan , J. Phys. Chem. Lett. 2022, 13, 3112–3120. 35357183 10.1021/acs.jpclett.2c00316 PMC7612738

[96] L. Cardo , V. Sadovnikova , S. Phongtongpasuk , N. J. Hodges , M. J. Hannon , Chem. Commun. 2011, 47, 6575–6577. 10.1039/c1cc11356a 21556402

[97] A. Fernandes , A. Viterisi , V. Aucagne , D. A. Leigh , S. Papot , Chem. Commun. 2012, 48, 2083–2085. 10.1039/c2cc17458h 22227715

[98] L. Melidis , H. J. Hill , N. J. Coltman , S. P. Davies , K. Winczura , T. Chauhan , J. S. Craig , A. Garai , C. A. J. Hooper , R. T. Egan , J. A. McKeating , N. J. Hodges , Z. Stamataki , P. Grzechnik , M. J. Hannon , Angew. Chem. Int. Ed. 2021, 60, 18144–18151. 10.1002/anie.202104179 PMC8222931 33915014

[99] S. Phongtongpasuk , S. Paulus , J. Schnabl , R. K. O. Sigel , B. Spingler , M. J. Hannon , E. Freisinger , Angew. Chem. Int. Ed. 2013, 52, 11513–11516. 10.1002/anie.201305079 24039102

[100] L. Cardo , I. Nawroth , P. J. Cail , J. A. McKeating , M. J. Hannon , Sci. Reps. 2018, 8, 13342. 10.1038/s41598-018-31513-3 PMC6127258 30190568

[101] B. Nordén , T. Kurucsev , J. Mol. Recognit. 1994, 7, 141–155. 7826674 10.1002/jmr.300070211

[102] A. Rodger , B. Norden , Circular Dichroism and Linear Dichroism, Oxford University Press, Oxford, UK 1997.

[103] M. Vorlíčková , I. Kejnovská , K. Bednářová , D. Renčiuk , J. Kypr , Chirality 2012, 24, 691–698. 22696273 10.1002/chir.22064

[104] M. J. Hannon , V. Moreno , M. J. Prieto , E. Moldrheim , E. Sletten , I. Meistermann , C. J. Isaac , K. J. Sanders , A. Rodger , Angew. Chem. Int. Ed. 2001, 40, 879–884. 10.1002/1521-3773(20010302)40:5<879::AID-ANIE879>3.0.CO;2-X 29712178

[105] I. Meistermann , V. Moreno , M. J. Prieto , E. Moldrheim , E. Sletten , S. Khalid , P. M. Rodger , J. C. Peberdy , C. J. Isaac , A. Rodger , M. J. Hannon , Proc. Natl. Acad. Sci. USA 2002, 99, 5069–5074. 11959957 10.1073/pnas.062634499 PMC122723

[106] G. I. Pascu , PhD thesis, University of Birmingham (UK) 2008.

[107] L. A. Carpino , S. A. Triolo , R. A. Berglund , J. Org. Chem. 1989, 54, 3303–3310.

[108] R. T. Borchardt , L. A. Cohen , J. Am. Chem. Soc. 1972, 94, 9175–9182. 4642367 10.1021/ja00781a031

[109] M. D. Hanwell , D. E. Curtis , D. C. Lonie , T. Vandermeersch , E. Zurek , G. R. Hutchison , J. Cheminf 2012, 4, 17. 10.1186/1758-2946-4-17 PMC3542060 22889332

[110] P. Li , K. M. Merz Jr. , J. Chem. Inf. Model. 2016, 56, 599–604. 26913476 10.1021/acs.jcim.5b00674

[111] G. W. T. M. J. Frisch , H. B. Schlegel , G. E. Scuseria , M. A. Robb , J. R. Cheeseman , G. Scalmani , V. Barone , G. A. Petersson , H. Nakatsuji , X. Li , M. Caricato , A. V. Marenich , J. Bloino , B. G. Janesko , R. Gomperts , B. Mennucci , H. P. Hratchian , J. V. Ortiz , A. F. Izmaylov , J. L. Sonnenberg , F. D. Williams , F. Lipparini , F. Egidi , J. Goings , B. Peng , A. Petrone , T. Henderson , D. Ranasinghe , V. G. Zakrzewski , J. Gao , et al., Gaussian, Inc, 2016.

[112] F. Neese , F. Wennmohs , U. Becker , C. Riplinger , J. Chem. Phys. 2020, 152, 224108. 32534543 10.1063/5.0004608

[113] D. A. Case , H. M. Aktulga , K. Belfon , D. S. Cerutti , G. A. Cisneros , V. W. D. Cruzeiro , N. Forouzesh , T. J. Giese , A. W. Götz , H. Gohlke , S. Izadi , K. Kasavajhala , M. C. Kaymak , E. King , T. Kurtzman , T.‐S. Lee , P. Li , J. Liu , T. Luchko , R. Luo , M. Manathunga , M. R. Machado , H. M. Nguyen , K. A. O'hearn , A. V. Onufriev , F. Pan , S. Pantano , R. Qi , A. Rahnamoun , A. Risheh , et al., J. Chem. Inf. Model. 2023, 63, 6183–6191. 37805934 10.1021/acs.jcim.3c01153 PMC10598796

[114] I. Ivani , P. D. Dans , A. Noy , A. Pérez , I. Faustino , A. Hospital , J. Walther , P. Andrio , R. Goñi , A. Balaceanu , G. Portella , F. Battistini , J. L. Gelpí , C. González , M. Vendruscolo , C. A. Laughton , S. A. Harris , D. A. Case , M. Orozco , Nat. Methods 2016, 13, 55–58. 26569599 10.1038/nmeth.3658 PMC4700514

[115] K. C. Woods , S. S. Martin , V. C. Chu , E. P. Baldwin , J. Mol. Biol. 2001, 313, 49–69. 11601846 10.1006/jmbi.2001.5012

[116] M. J. Abraham , T. Murtola , R. Schulz , S. Páll , J. C. Smith , B. Hess , E. Lindahl , SoftwareX 2015, 1‐2, 19–25.

[117] The PyMOL Molecular Graphics System, Version 2, Vol. 8, Schrödinger, LLC.

