# Supplementary material for: Switching on Supramolecular DNA Junction Binding Using a Human Enzyme
Source: Angew Chem Int Ed Engl. 2025 Mar 23;64(21):e202503683. doi: 10.1002/anie.202503683 (PMC12087844; doi:10.1002/anie.202503683)
Supplement: Supplementary file 1 — Supporting Information [file ANIE-64-e202503683-s001.pdf]

Supporting Information
©Wiley-VCH 2021
69451 Weinheim, Germany

Switching On Supramolecular DNA Junction Binding Using a Human Enzyme

Subhendu Karmakar[a], Samuel J. Dettmer[a], Catherine A. J. Hooper[a], Nikolas J. Hodges[b] and Michael J. Hannon[a]*

[a] School of Chemistry, University of Birmingham, Edgbaston, Birmingham B15 2TT, United Kingdom

[b] School of Biosciences, University of Birmingham, Edgbaston, Birmingham B15 2TT, United Kingdom

E-mail: m.j.hannon@bham.ac.uk

DOI: 10.1002/anie.2021XXXXX

Table of Contents

Scheme S1: Synthetic scheme to prepare the capped and uncapped metallo-cylinders. 5
Figure S1: ESI-MS (+ve) of $[Fe_2(L1)_3](PF_6)_4$.......... 6
Figure S2: ESI-MS (+ve) of $[Fe_2(L1)_3]Cl_4$. 6
Figure S3: ESI-MS (+ve) of $[Ni_2(L1)_3](PF_6)_4$.......... 7
Figure S4: ESI-MS (+ve) of $[Ni_2(L1)_3]Cl_4$. 7
Figure S5: ESI-MS (+ve) of $[Fe_2(L2)_3](PF_6)_4$.......... 8
Figure S6: ESI-MS (+ve) of $[Fe_2(L2)_3]Cl_4$. 8
Figure S7: ESI-MS (+ve) of $[Ni_2(L2)_3](PF_6)_4$.......... 9
Figure S8: ESI-MS (+ve) of $[Ni_2(L2)_3]Cl_4$. 9
Figure S9: ^{1}H NMR (400 MHz) of diethyl pyridine-2,5-dicarboxylate in $CDCl_3$. 10
Figure S10: ^{1}H NMR (400 MHz) of 2,5-dihydroxymethylpyridine in Methanol-d_4. 10
Figure S11: ^{1}H NMR (400 MHz) of 5-hydroxymethyl-2-pyridinecarboxaldehyde in DMSO-d_6. 11
Figure S12: ^{1}H NMR (400 MHz) of L1 in DMSO-d_6. 11
Figure S13: ^{1}H NMR (400 MHz) of 6-Hydroxy-4,4,5,7,8-pentamethylhydrocoumarin in $CDCl_3$. 12
Figure S14: ^{1}H NMR (400 MHz) of QPA in $CDCl_3$. 12
Figure S15: ^{1}H NMR (300 MHz) of 5-hydroxymethyl-2-pyridinecarboxaldehyde and QPA coupling product in $CDCl_3$. 13
Figure S16: ^{1}H NMR (300 MHz) of L2 in CD_2Cl_2. 13
Figure S17: ^{1}H NMR (400 MHz) of $[Fe_2(L1)_3](PF_6)_4$ in CD_3CN. 14
Figure S18: COSY (400 MHz) of $[Fe_2(L1)_3](PF_6)_4$ in CD_3CN. 14
Figure S19: NOESY (400 MHz) of $[Fe_2(L1)_3](PF_6)_4$ in CD_3CN. 15
Figure S20: ^{1}H NMR (400 MHz) of $[Fe_2(L1)_3]Cl_4$ in Methanol-d_4. 15
Figure S21: ^{1}H NMR (500 MHz) of $[Fe_2(L2)_3](PF_6)_4$ in CD_2Cl_2. 16
Figure S22: COSY (500 MHz) of $[Fe_2(L2)_3](PF_6)_4$ in CD_2Cl_2. 16
Figure S23: NOESY (500 MHz) of $[Fe_2(L2)_3](PF_6)_4$ in CD_2Cl_2. 17
Figure S24: ^{1}H NMR (400 MHz) of $[Fe_2(L2)_3]Cl_4$ in Methanol-d_4. 17
Figure S25: ^{1}H NMR (400 MHz) of QPA Et in $CDCl_3$. 18
Figure S26: Stability study of $[Ni_2(L2)_3]Cl_4$ (A) and $[Ni_2(L1)_3]Cl_4$ (B) in 1x TA buffer pH 7.4 monitored by UV-Vis spectroscopy at 37 °C. 18
Figure S27: Stability study of $[Ni_2(L2)_3]Cl_4$ in water monitored by ESI-MS at 37 °C. 19
Figure S28: PAGE gel depicting the preformed room temperature stable 18-mer DNA 3WJ binding behaviour of Ni-cylinders. 19
Figure S29: PAGE gel showing the DNA 3WJ binding of Ni-cylinders QPA Et. 20
Figure S30: PAGE gel depicting the DNA 3WJ binding behaviour of capped cylinder, $[Ni_2(L2)_3]Cl_4$, at various incubation time points (1 h, 2 h & 4 h). 20

Figure S31: PAGE gel depicting the DNA 3WJ binding behaviour of capped cylinder, $[Ni_2(L2)_3]Cl_4$, at various incubation time points (6 h, 8 h & 10 h)....................21

Figure S32: Variation in the intensity of DNA 3WJ bands with different incubation periods and increasing concentrations in capped cylinder, $[Ni_2(L2)_3]Cl_4$. 21

Figure S33: PAGE gel illustrating the effect of NADPH and GSH, on the DNA 3WJ binding of capped cylinder, $[Ni_2(L2)_3]^{4+}$....................22

Figure S34: UV melting curves of DNA 3WJ in presence and absence of capped and uncapped cylinders respectively....................22

Figure S35: UV melting curves of RNA 3WJ in presence (right) and absence (left) of uncapped cylinder. 23

Figure S36: PAGE gel showing the RNA 3WJ binding of capped and uncapped Ni-cylinders....................23

Figure S37: Distance plots showing the minimum distance between any central CH_2 (in the cylinder core) and any quinone (defined as any non-hydrogen atom in or attached directly to the quinone ring) in each simulation of the capped cylinder alone (A) and the capped cylinder with 3WJ DNA (B)....................24

Figure S38: RMSD plots of the QPA caps (calculated by selecting all non-hydrogen atoms in or directly attached to the quinone rings) of the capped cylinder in MD simulations with no DNA (top left) and simulations with 3WJ DNA (top right). A representative comparison plot is also shown (bottom).. 25

Figure S39: Molecular dynamics snapshots of A) the uncapped cylinder residing in the 3WJ and B) the capped cylinder residing in the 3WJ. 26

Table S1: Cytotoxicity data of capped & uncapped cylinders and cisplatin....................27

Figure S40: Comparison of cell viability (%) of dicoumarol (25 & 50 μM respectively) treated cells with respect to untreated cells.. 27

Table S2: Comparison of cell viability of dicoumarol (25 & 50 μM respectively) treated cells with respect to untreated cells....................28

Figure S41: Difference in cellular accumulation of capped ($[Ni_2(L2)_3]Cl_4$) and uncapped cylinder ($[Ni_2(L1)_3]Cl_4$) after treatment with same concentration of both the cylinders respectively for 24 h. 28

Figure S42: Stability study of a mixture of $[Ni_2(L2)_3]Cl_4$ and NADPH (8 eqv) in water monitored by ESI-MS at 37 °C.. 29

Figure S43: Stability study of a mixture of $[Ni_2(L2)_3]Cl_4$ and GSH (8 eqv) in water monitored by ESI-MS at 37 °C. 29

Figure S44: Stability study of $[Ni_2(L1)_3]Cl_4$ in water monitored by ESI-MS at 37 °C....................30

Figure S45: Stability study of a mixture of $[Ni_2(L1)_3]Cl_4$, NQO1 (20 μg/mL) and NADPH (8 eqv) in water monitored by ESI-MS at 37 °C.. 30

Figure S46: Stability study of a mixture of $[Ni_2(L1)_3]Cl_4$ and NADPH (8 eqv) in water monitored by ESI-MS at 37 °C.. 31

Figure S47: Stability study of a mixture of $[Ni_2(L1)_3]Cl_4$ and GSH (8 eqv) in water monitored by ESI-MS at 37 °C....................31

Figure S48: Stability study of a mixture of $[Ni_2(L2)_3]Cl_4$, NQO1 (20 μg/mL) and NADPH (8 eqv) in water monitored by ESI-MS at 37 °C. 32

CD & LD Study with DNA duplex....................33

Figure S49: CD (top) & LD (bottom) spectra of ctDNA displaying the interaction of B-DNA conformation upon interaction with uncapped $[Ni_2(L1)_3]^{4+}$ and capped $[Ni_2(L2)_3]^{4+}$ cylinders. 33

SUPPORTING INFORMATION

Experimental Procedures 34
Materials and Methods 34
Syntheses 34
Biophysical Experiments 37
Molecular Dynamics Simulations 39
Cell Line and Culture 39
Antiproliferative Activity 40
Cellular internalisation 40
Author Contributions 40
References 411

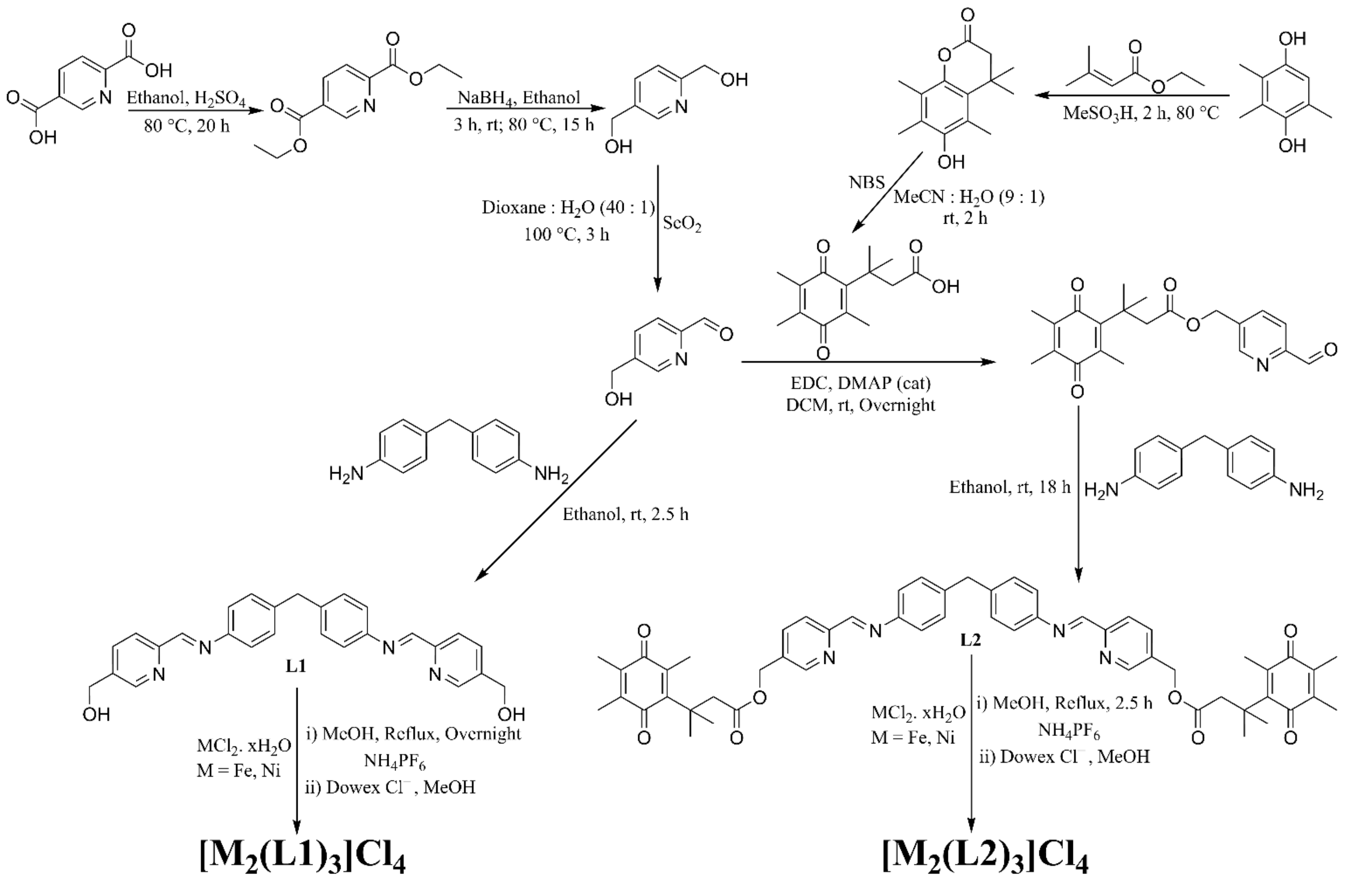

Scheme S1: Synthetic scheme to prepare the capped and uncapped metallo-cylinders.

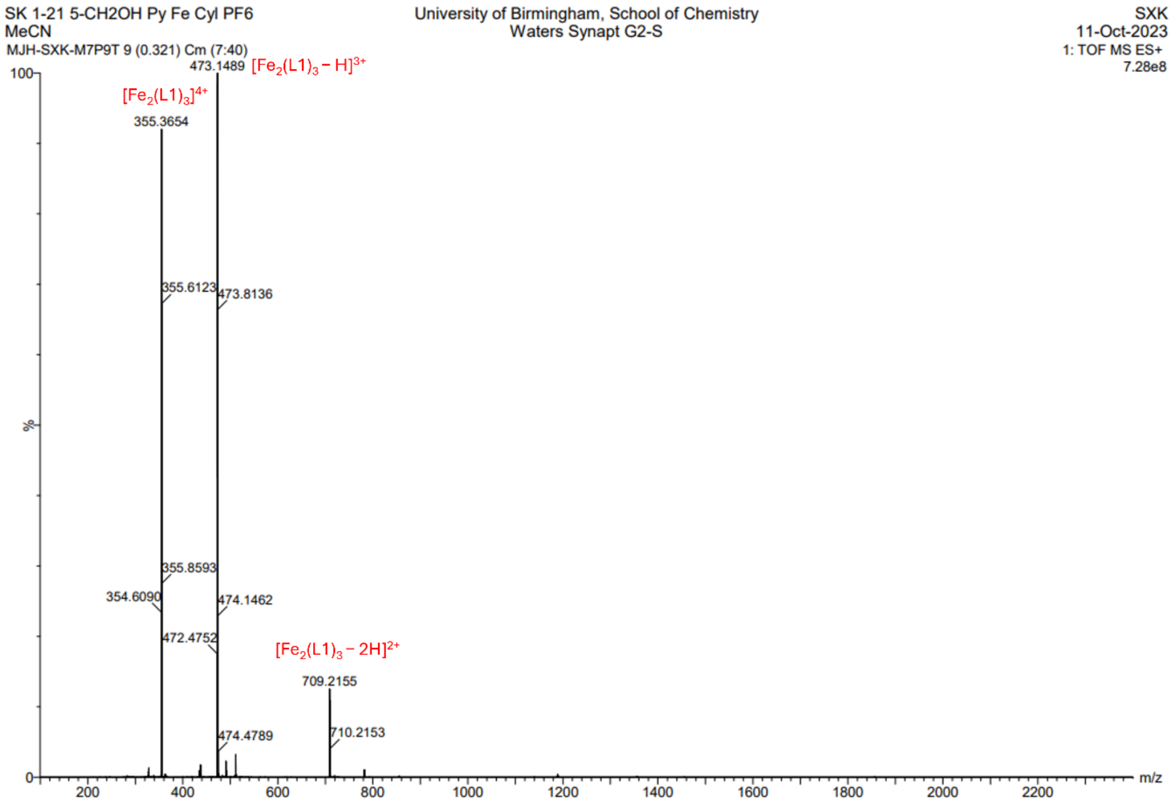

Figure S1: ESI-MS (+ve) of $[Fe_2(L1)_3](PF_6)_4$.

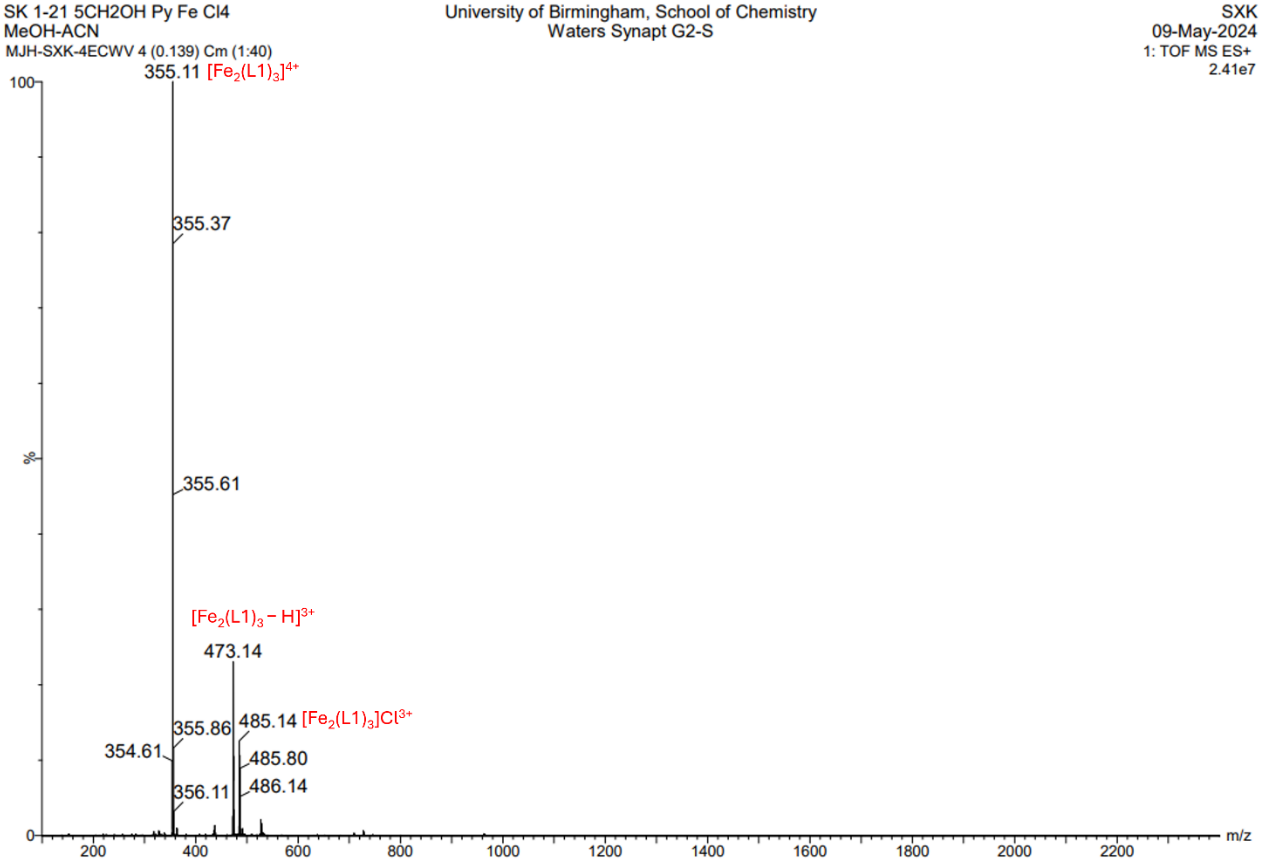

Figure S2: ESI-MS (+ve) of $[Fe_2(L1)_3]Cl_4$.

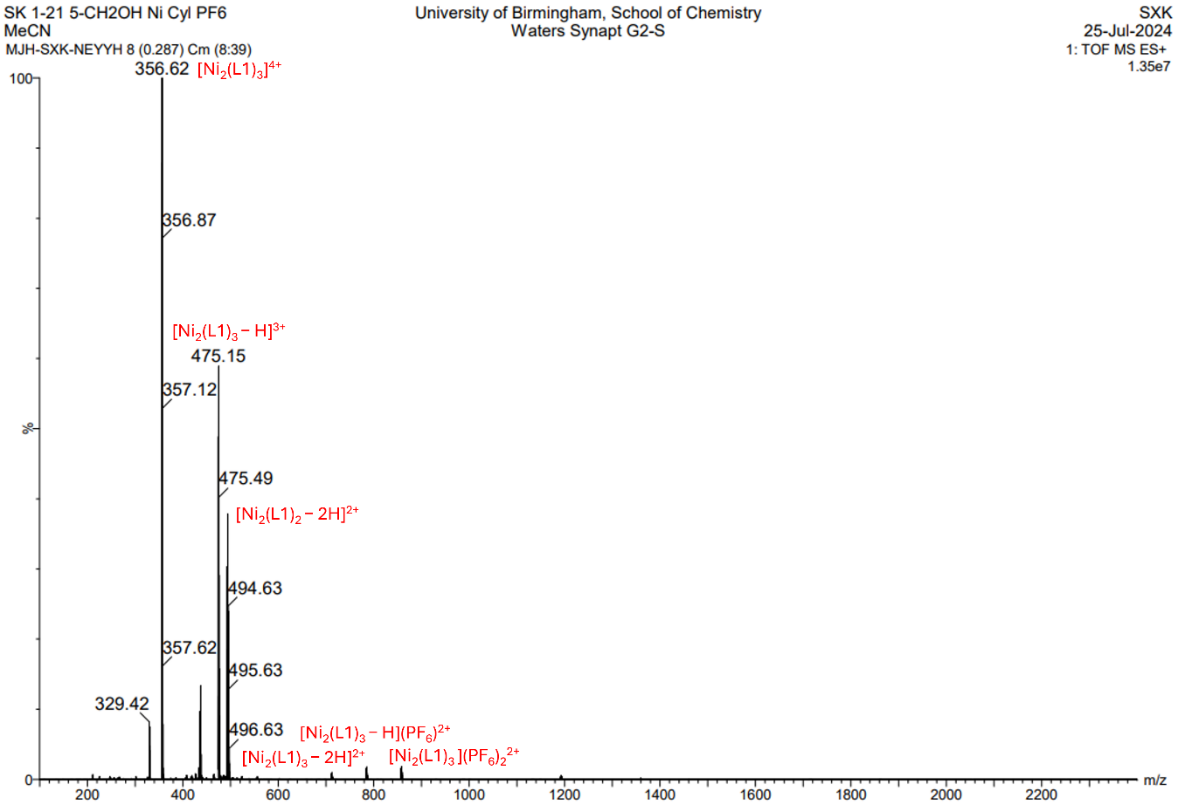

Figure S3: ESI-MS (+ve) of $[Ni_2(L1)_3](PF_6)_4$.

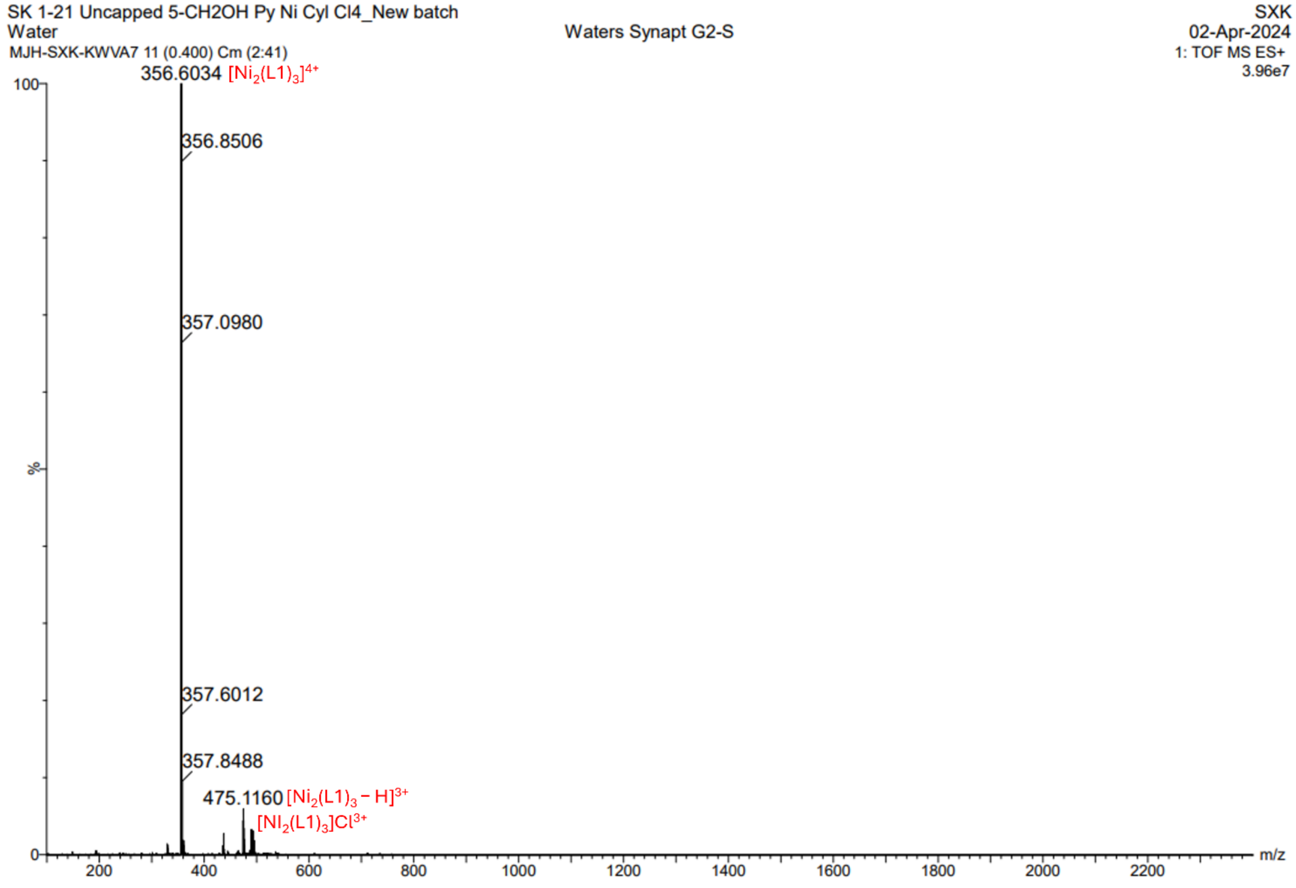

Figure S4: ESI-MS (+ve) of $[Ni_2(L1)_3]Cl_4$.

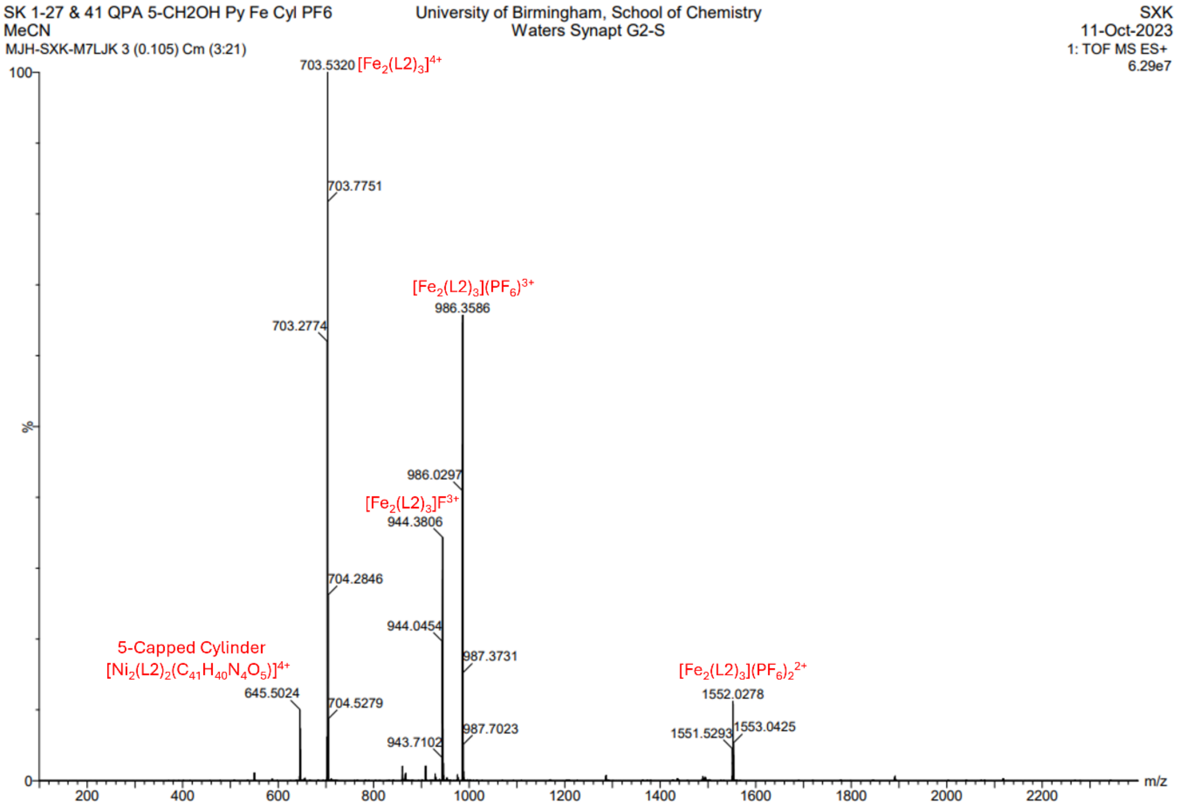

Figure S5: ESI-MS (+ve) of $[Fe_2(L2)_3](PF_6)_4$.

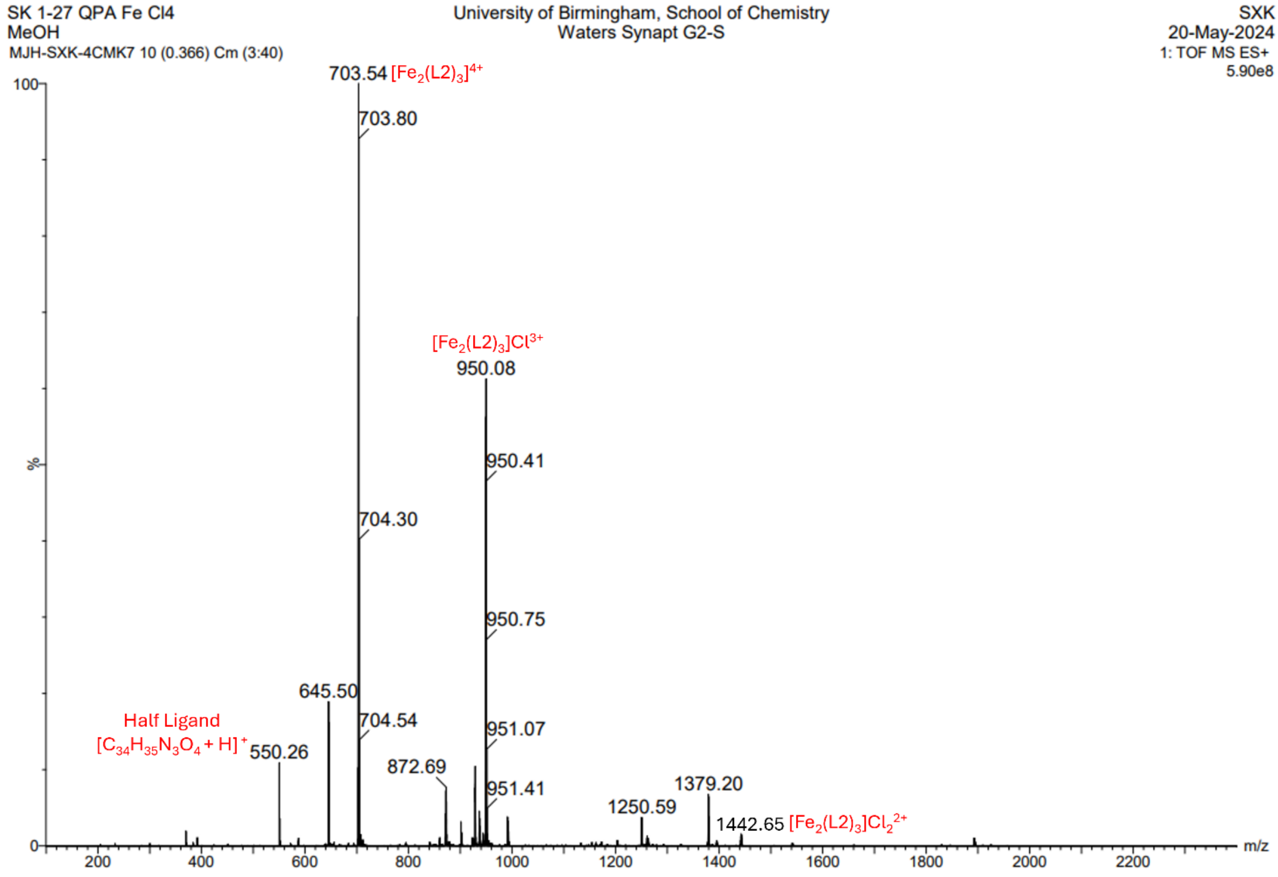

Figure S6: ESI-MS (+ve) of $[Fe_2(L2)_3]Cl_4$.

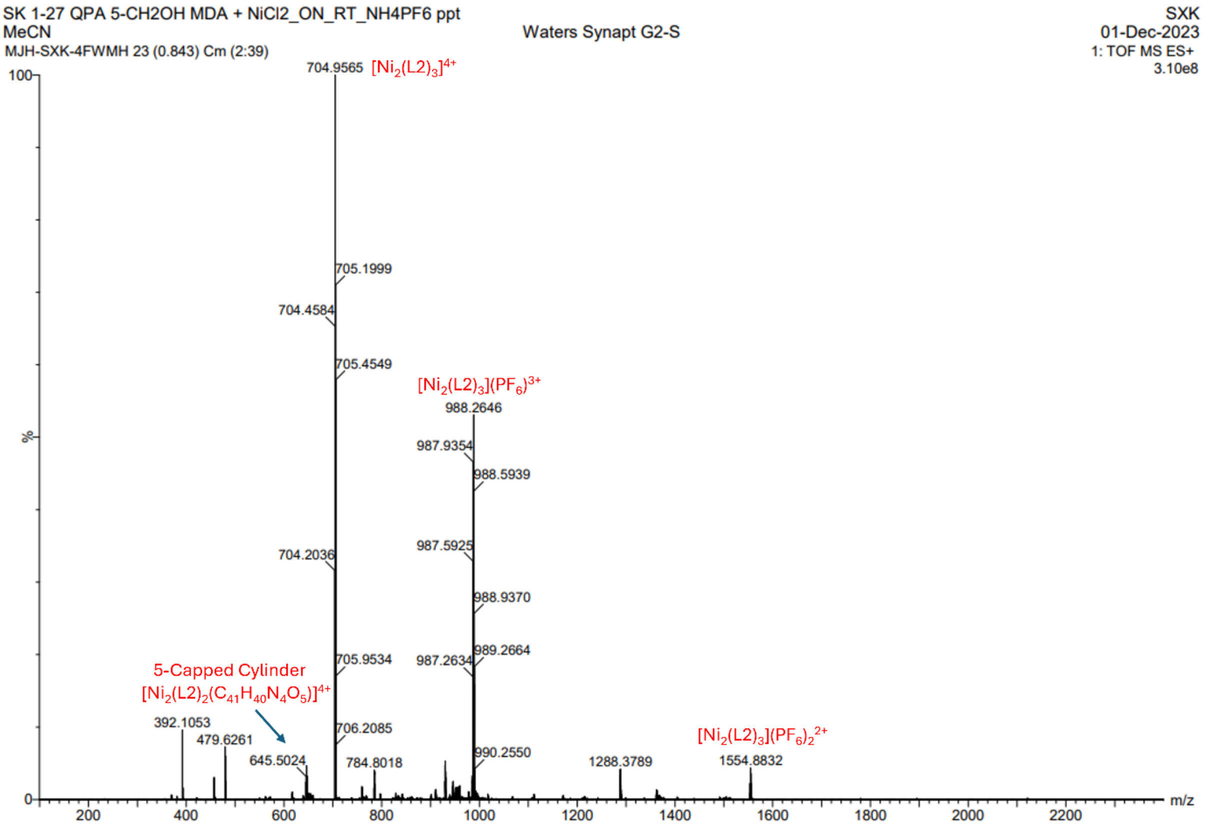

Figure S7: ESI-MS (+ve) of $[Ni_2(L2)_3](PF_6)_4$.

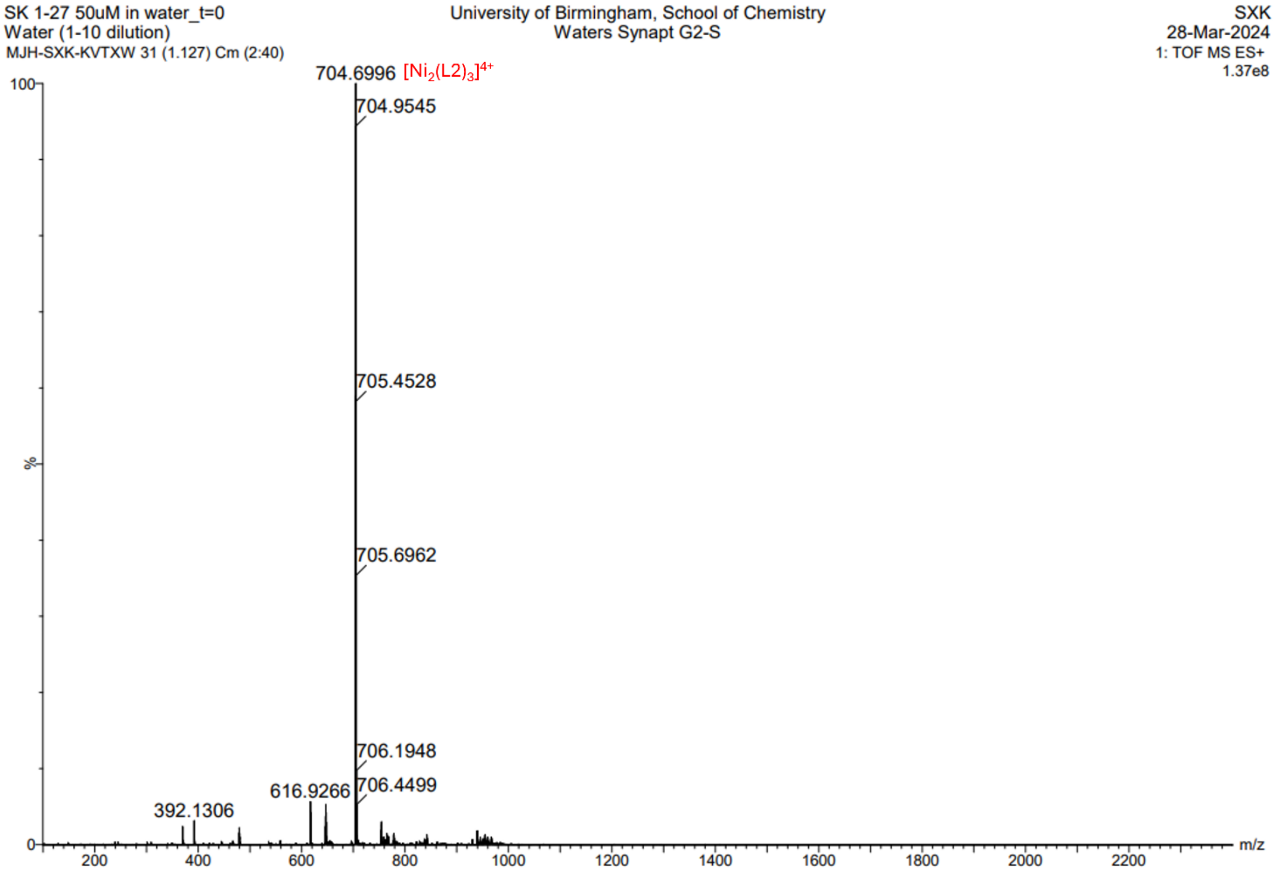

Figure S8: ESI-MS (+ve) of $[Ni_2(L2)_3]Cl_4$.

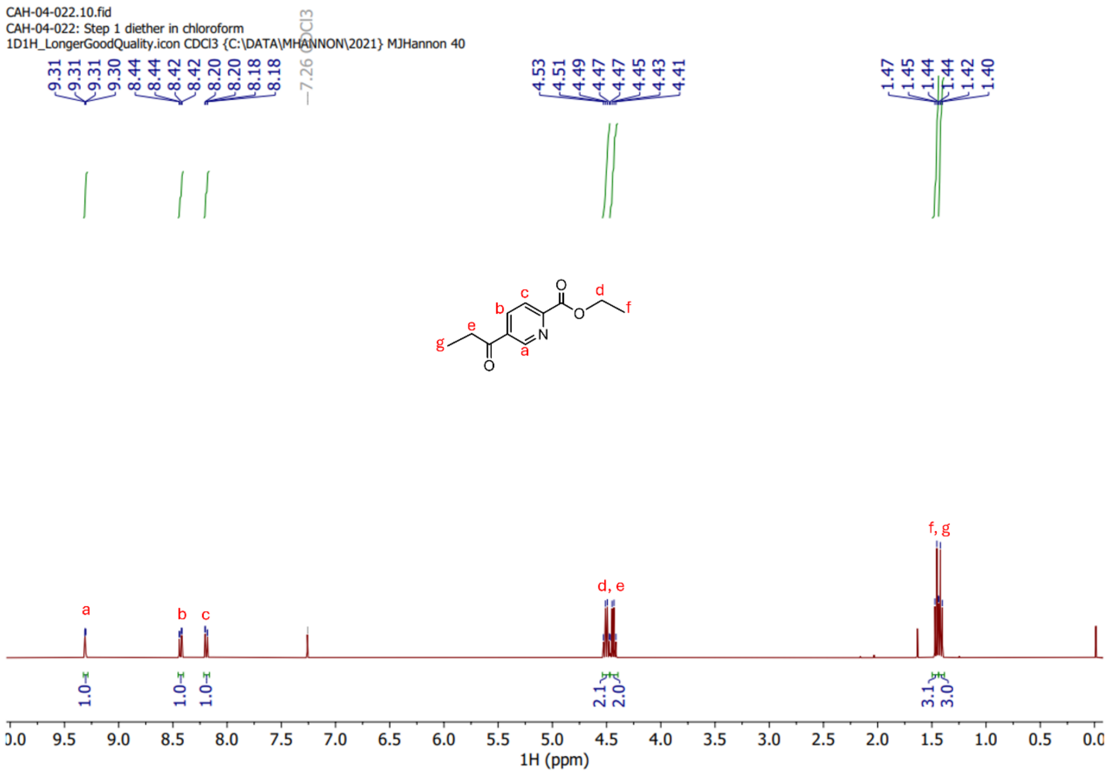

Figure S9: ^{1}H NMR (400 MHz) of diethyl pyridine-2,5-dicarboxylate in $CDCl_3$.

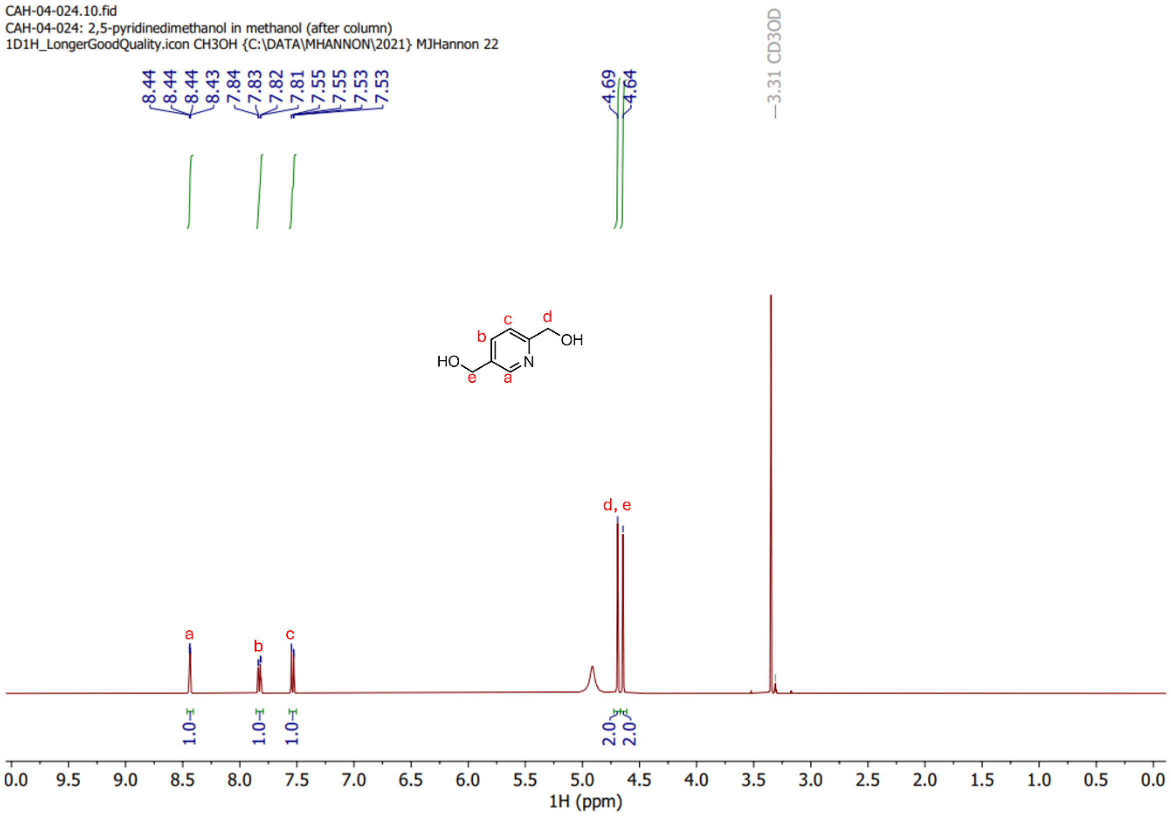

Figure S10: ^{1}H NMR (400 MHz) of 2,5-dihydroxymethylpyridine in Methanol-d_4.

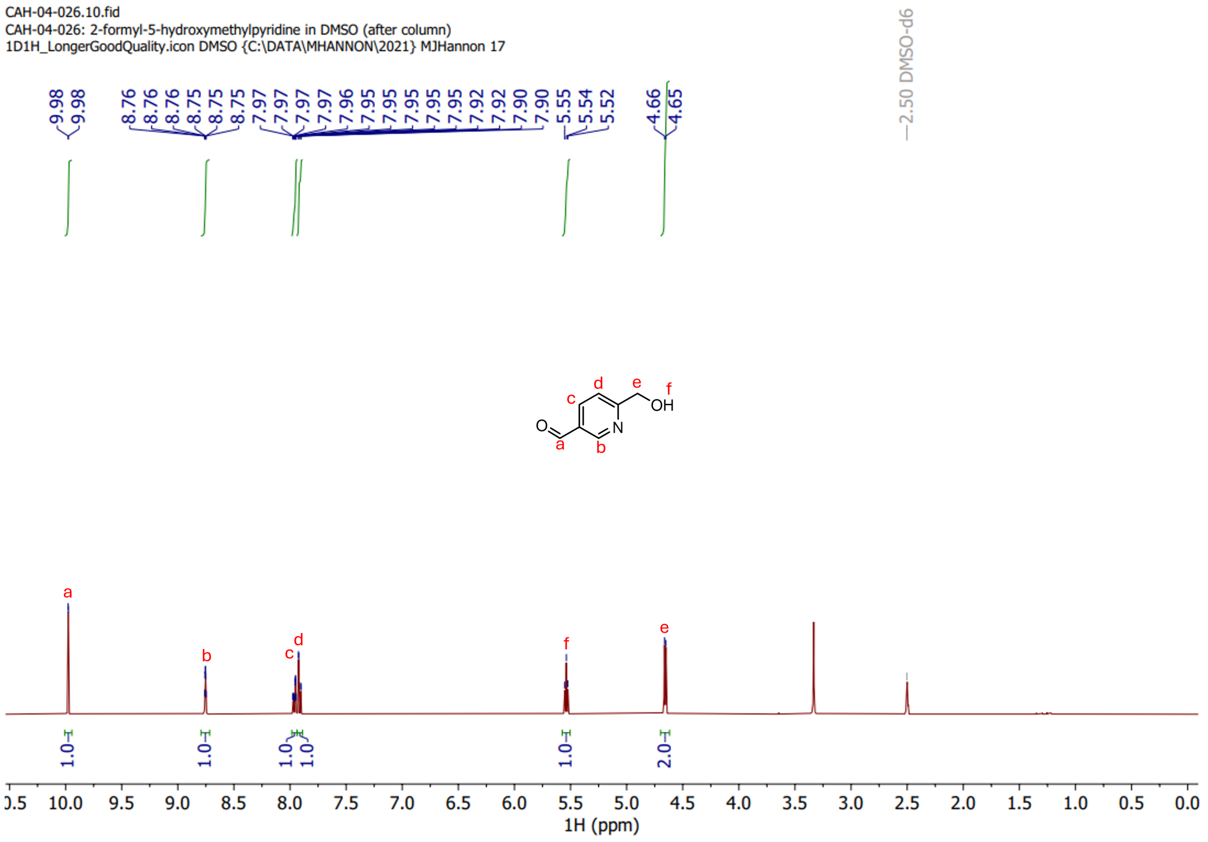

Figure S11: ^{1}H NMR (400 MHz) of 5-hydroxymethyl-2-pyridinecarboxaldehyde in DMSO-d_6.

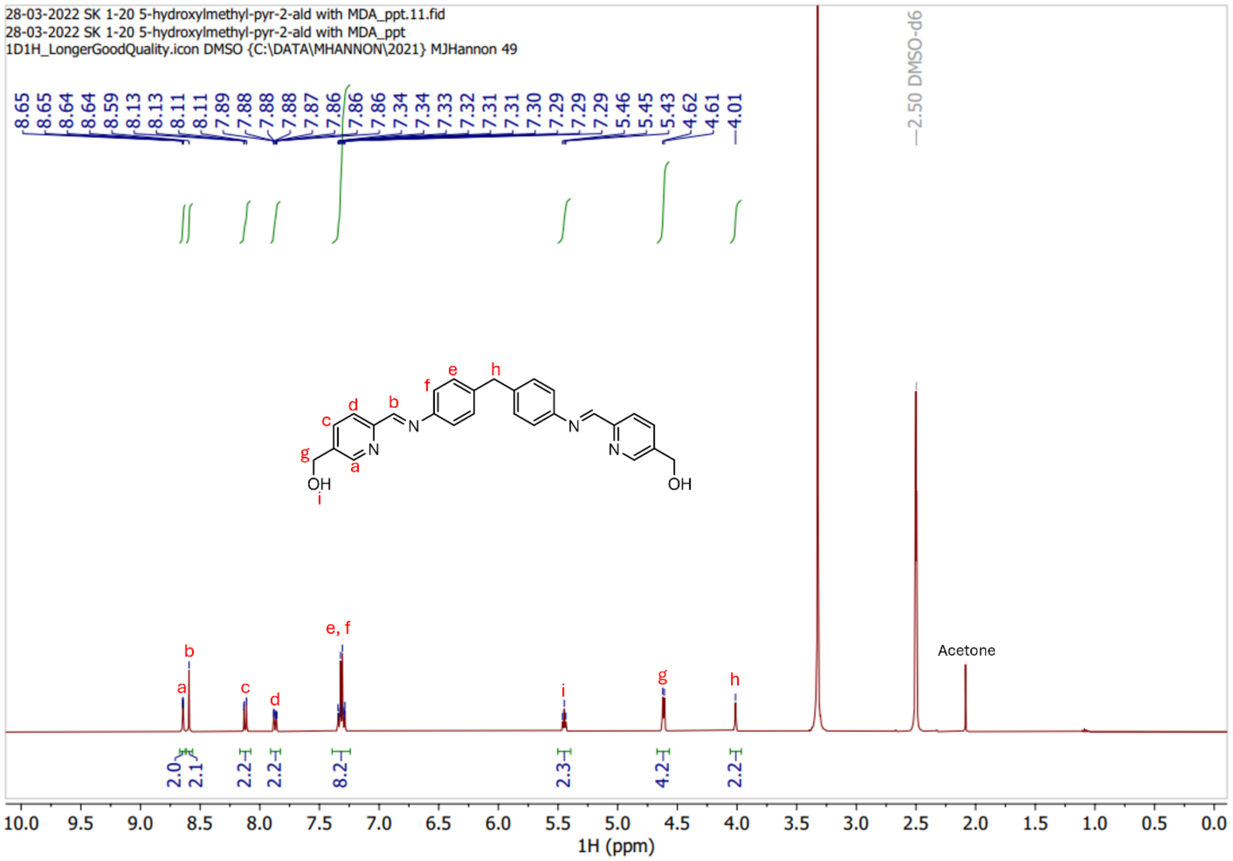

Figure S12: ^{1}H NMR (400 MHz) of L1 in DMSO-d_6.

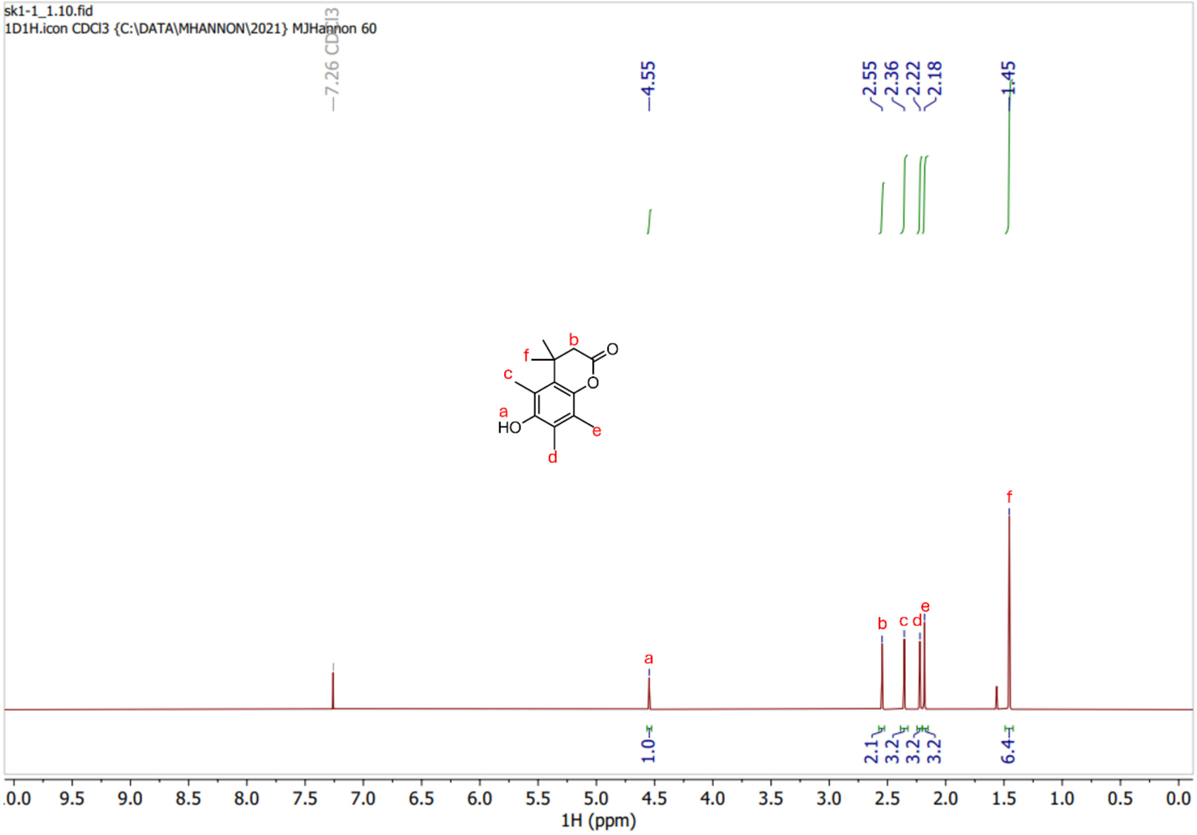

Figure S13: ^{1}H NMR (400 MHz) of 6-Hydroxy-4,4,5,7,8-pentamethylhydrocoumarin in $CDCl_3$.

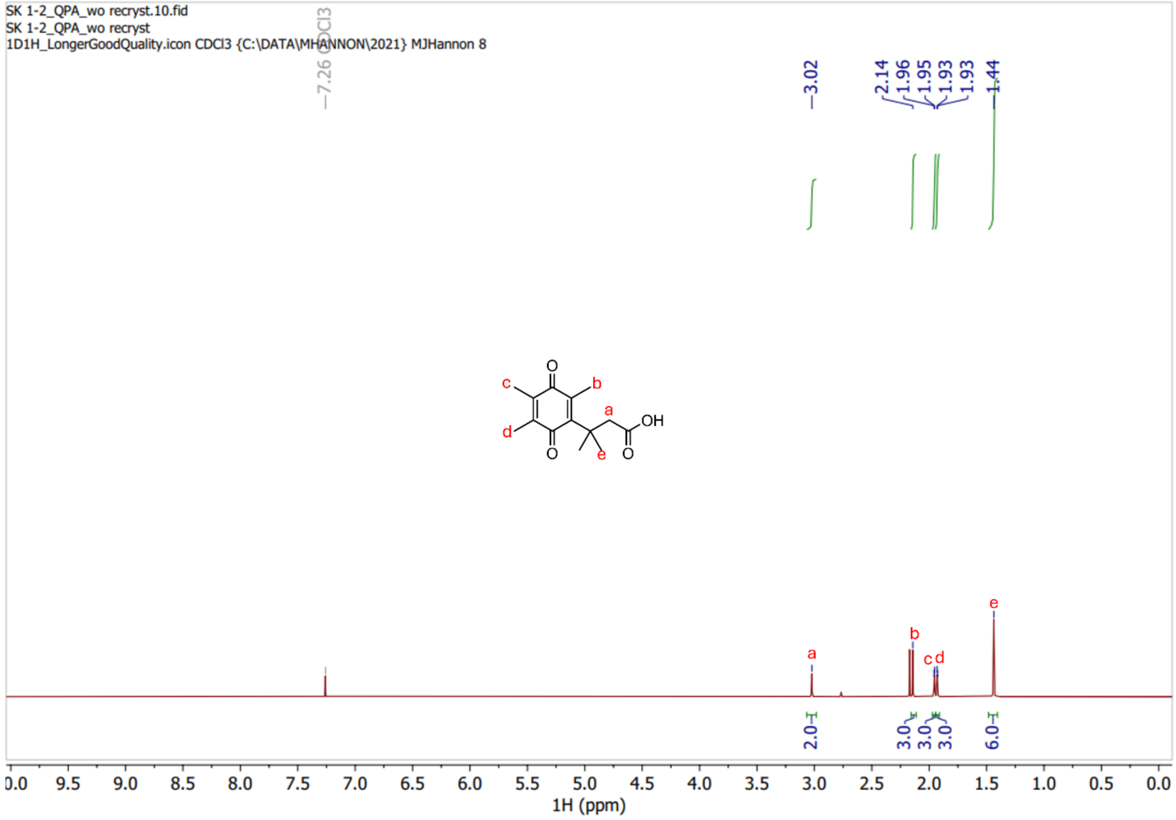

Figure S14: ^{1}H NMR (400 MHz) of QPA in $CDCl_3$.

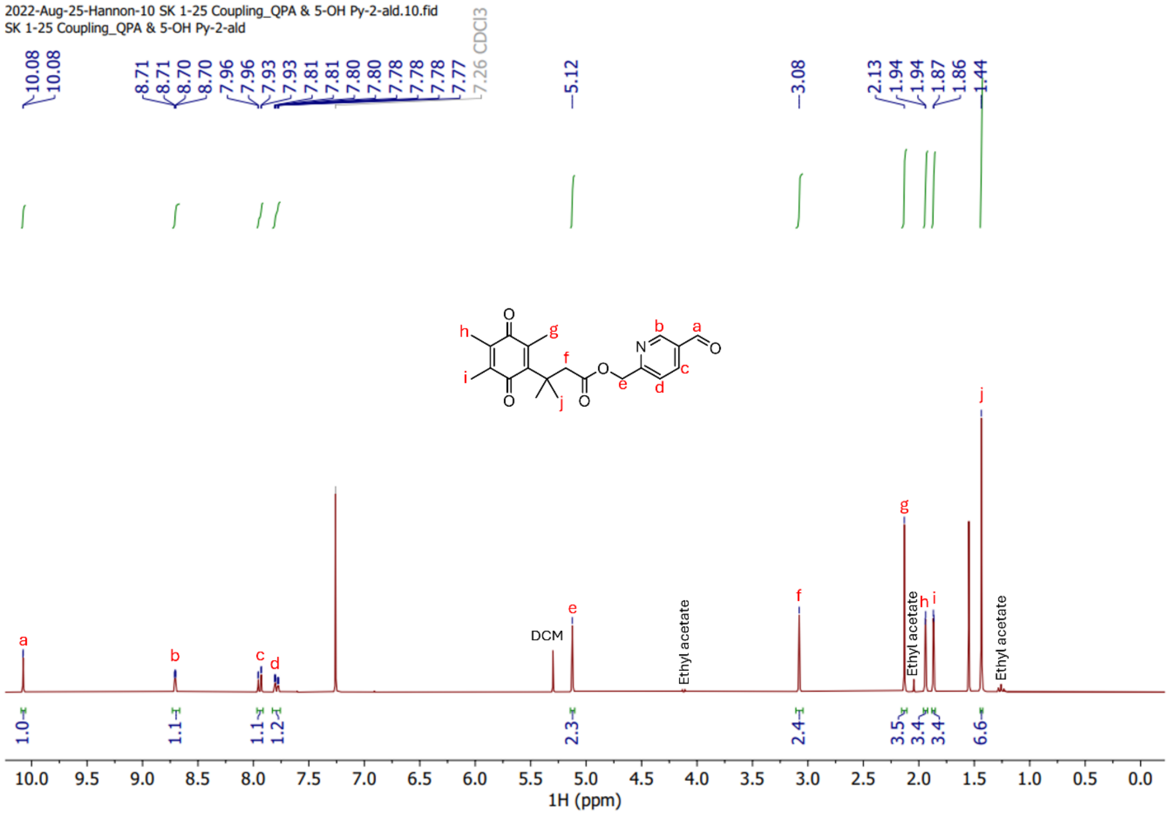

Figure S15: ^{1}H NMR (300 MHz) of 5-hydroxymethyl-2-pyridinecarboxaldehyde and QPA coupling product in $CDCl_3$.

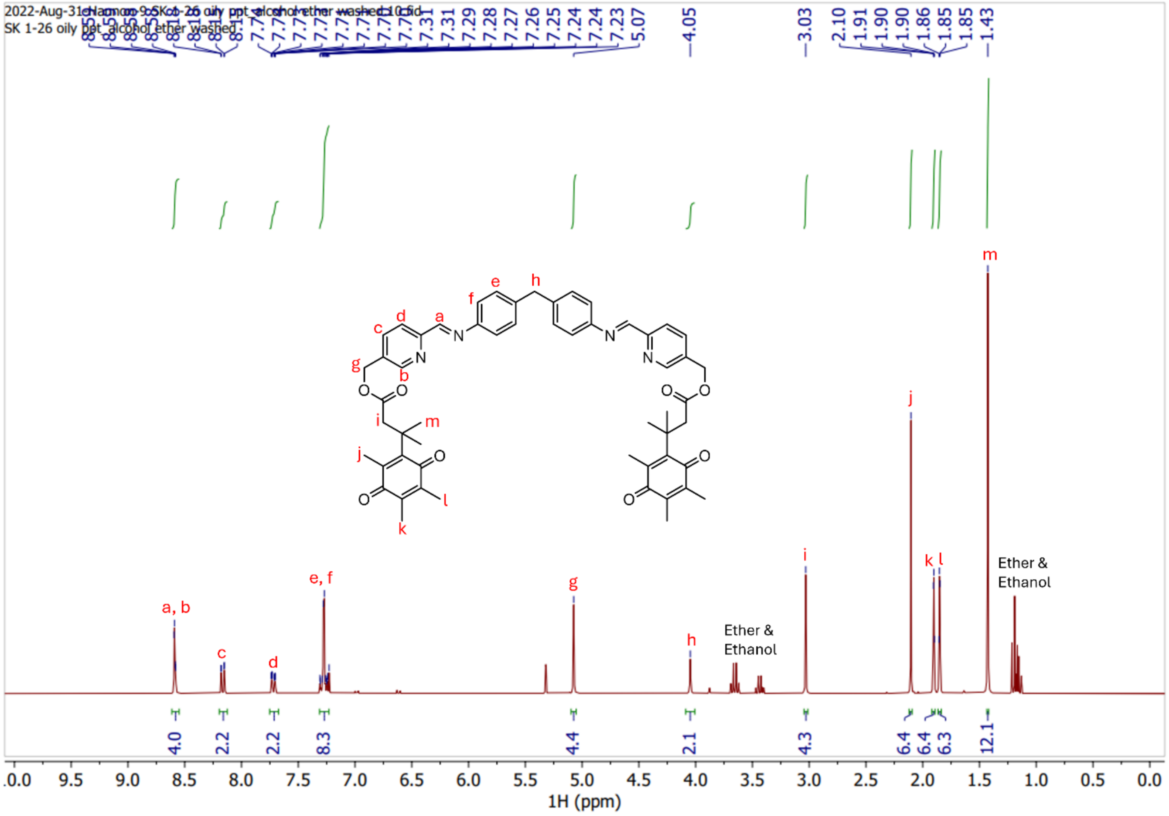

Figure S16: ^{1}H NMR (300 MHz) of L2 in CD_2Cl_2.

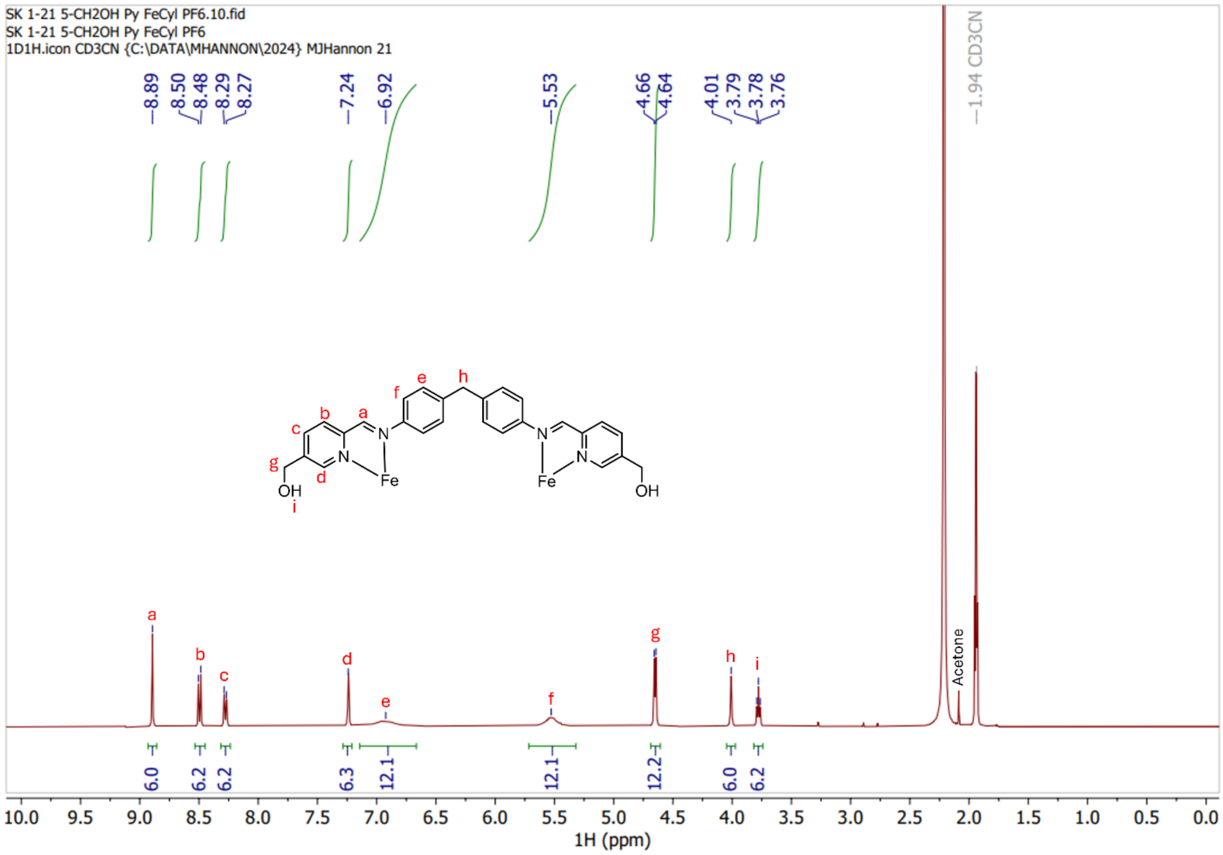

Figure S17: ^{1}H NMR (400 MHz) of $[Fe_2(L1)_3](PF_6)_4$ in CD_3CN.

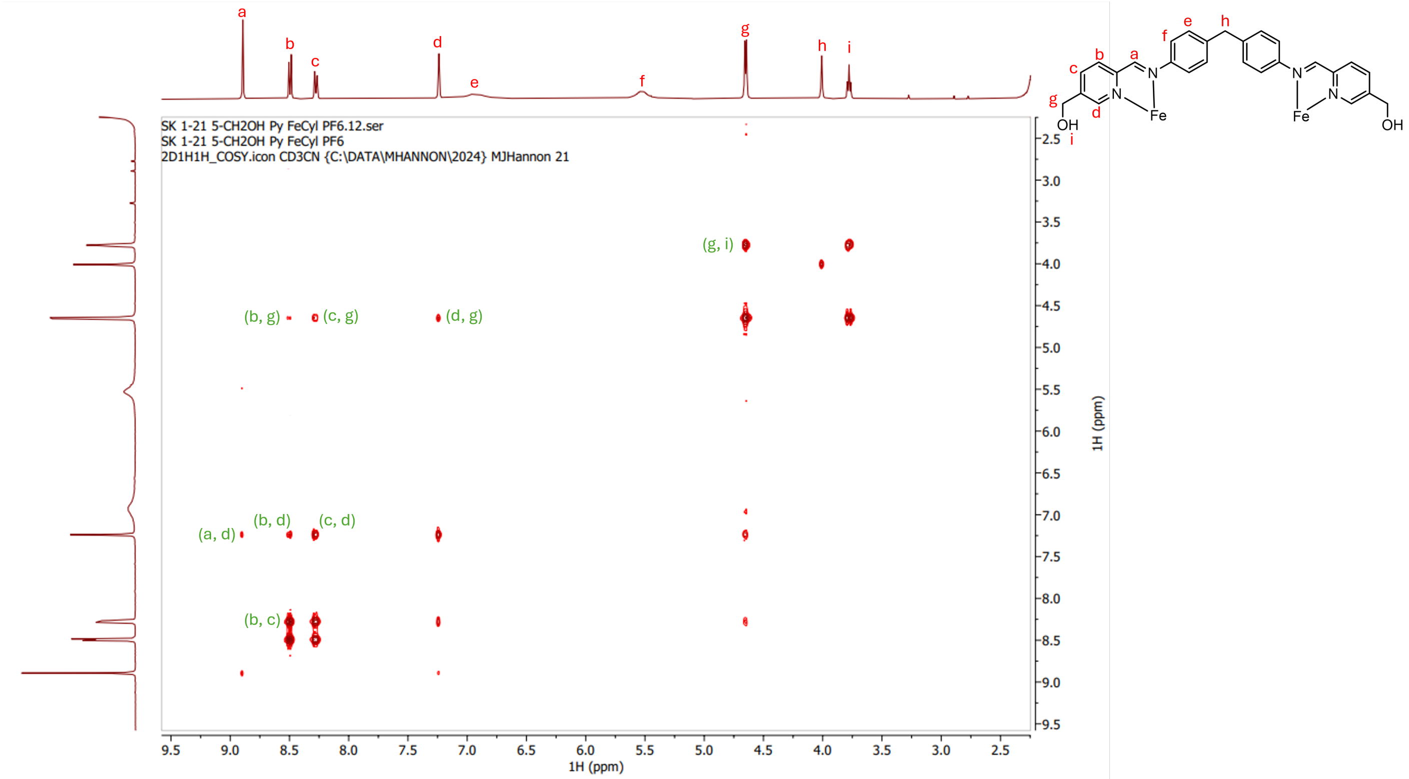

Figure S18: COSY (400 MHz) of $[Fe_2(L1)_3](PF_6)_4$ in CD_3CN.

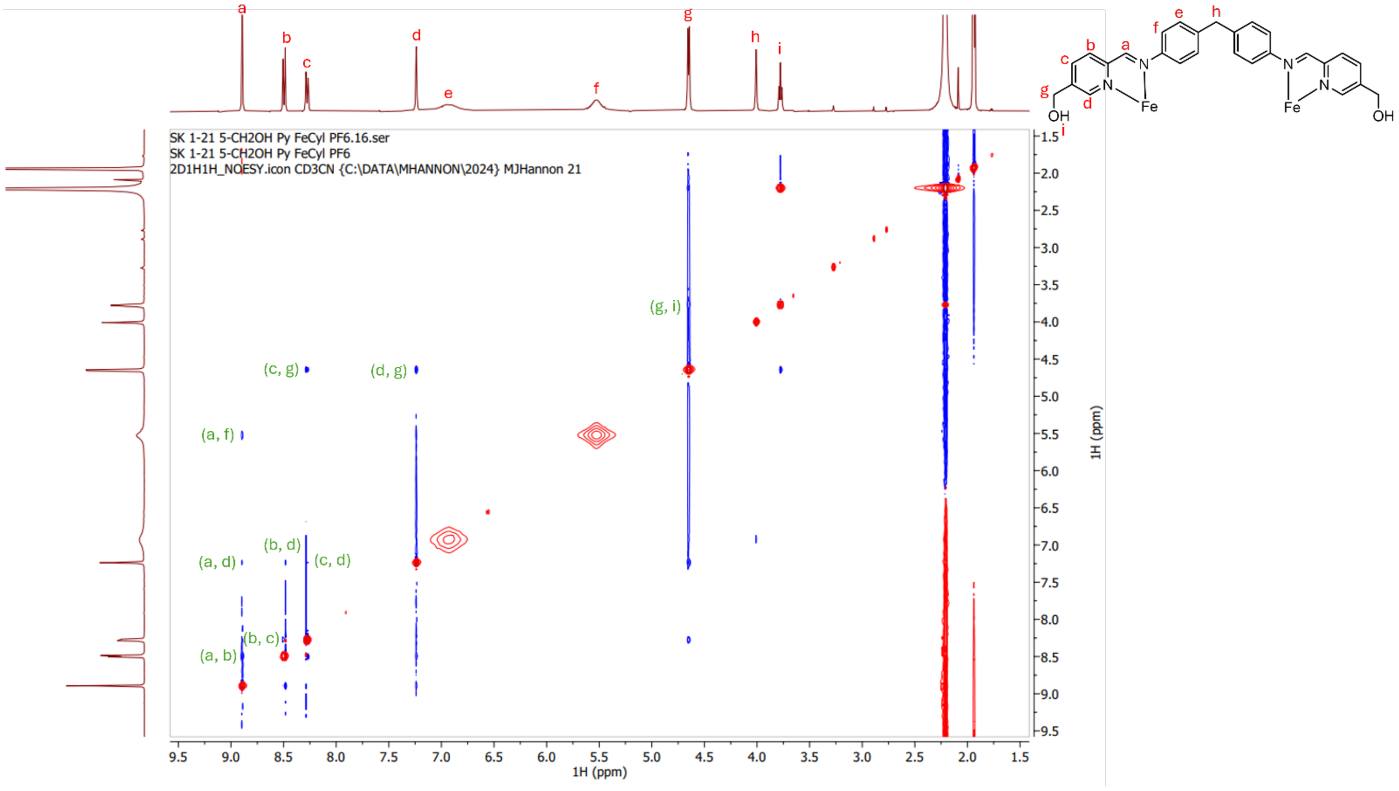

Figure S19: NOESY (400 MHz) of $[Fe_2(L1)_3](PF_6)_4$ in CD_3CN.

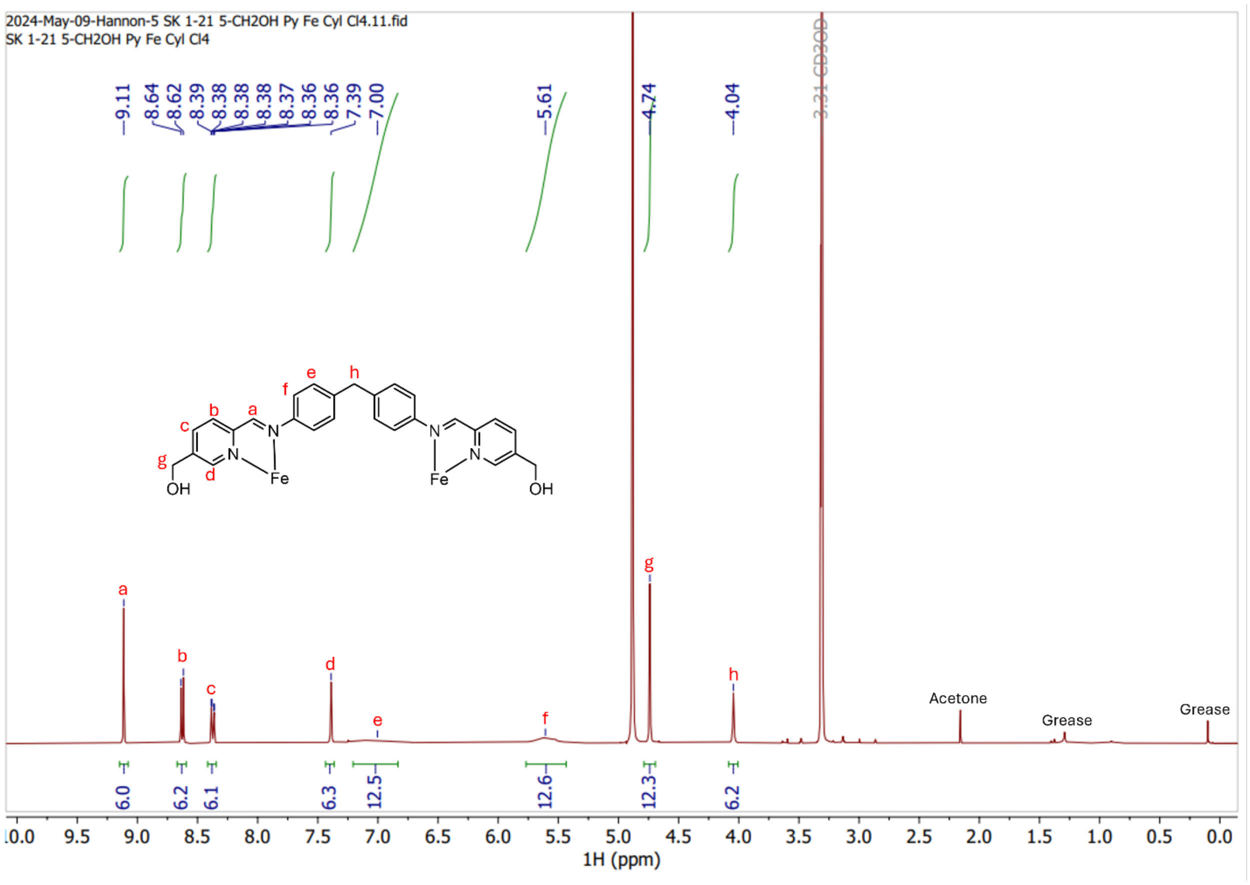

Figure S20: ^{1}H NMR (400 MHz) of $[Fe_2(L1)_3]Cl_4$ in Methanol-d_4.

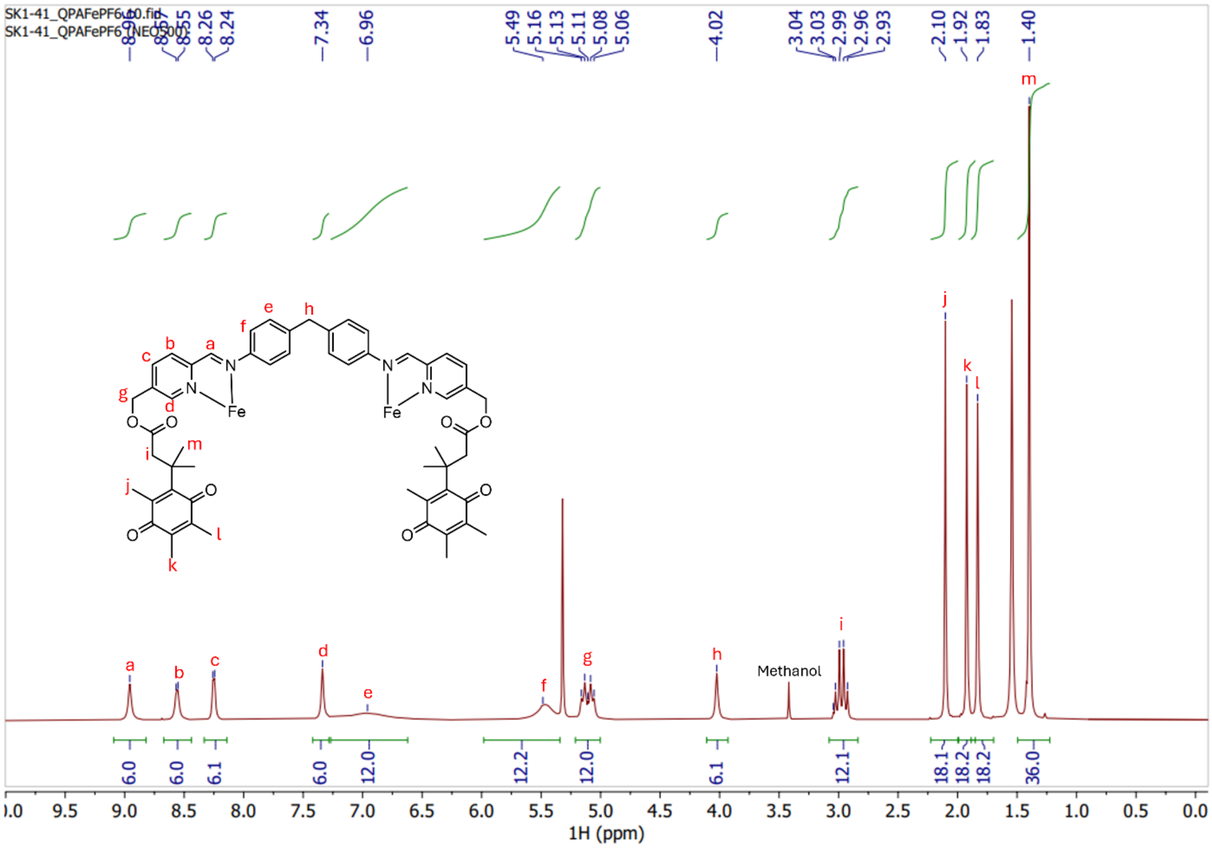

Figure S21: ^{1}H NMR (500 MHz) of $[Fe_2(L2)_3](PF_6)_4$ in CD_2Cl_2.

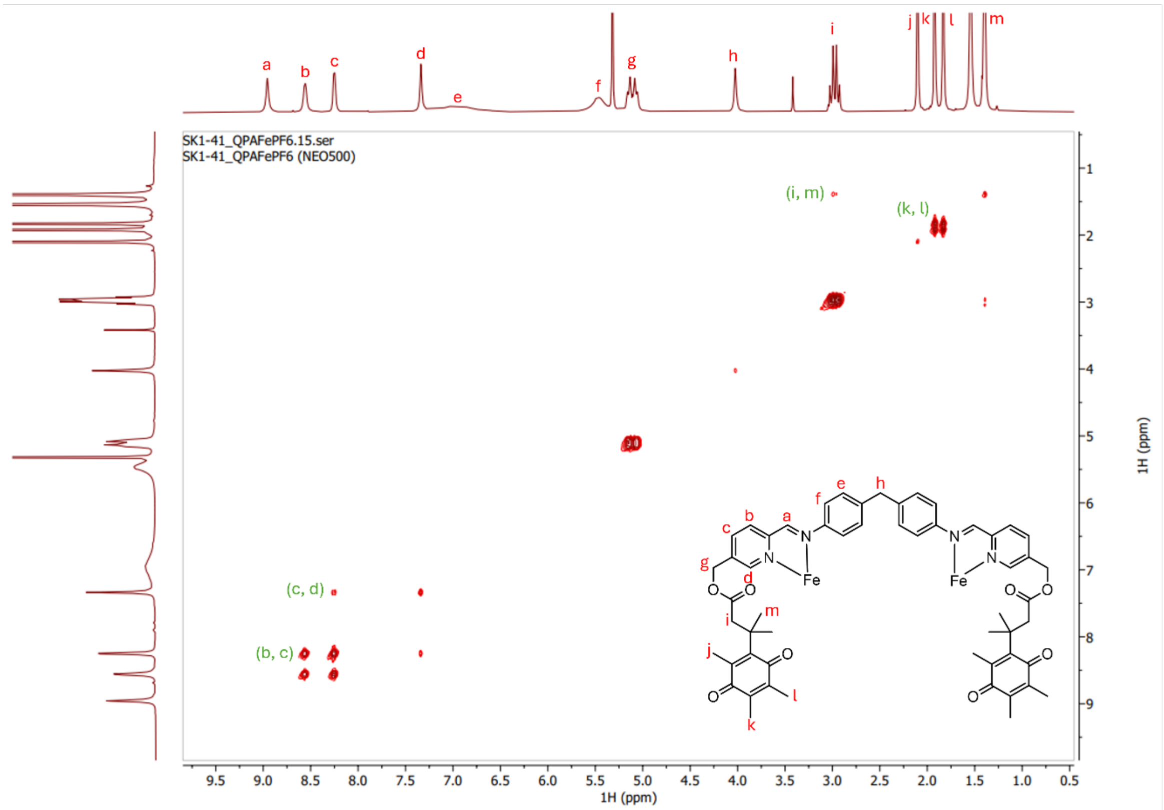

Figure S22: COSY (500 MHz) of $[Fe_2(L2)_3](PF_6)_4$ in CD_2Cl_2.

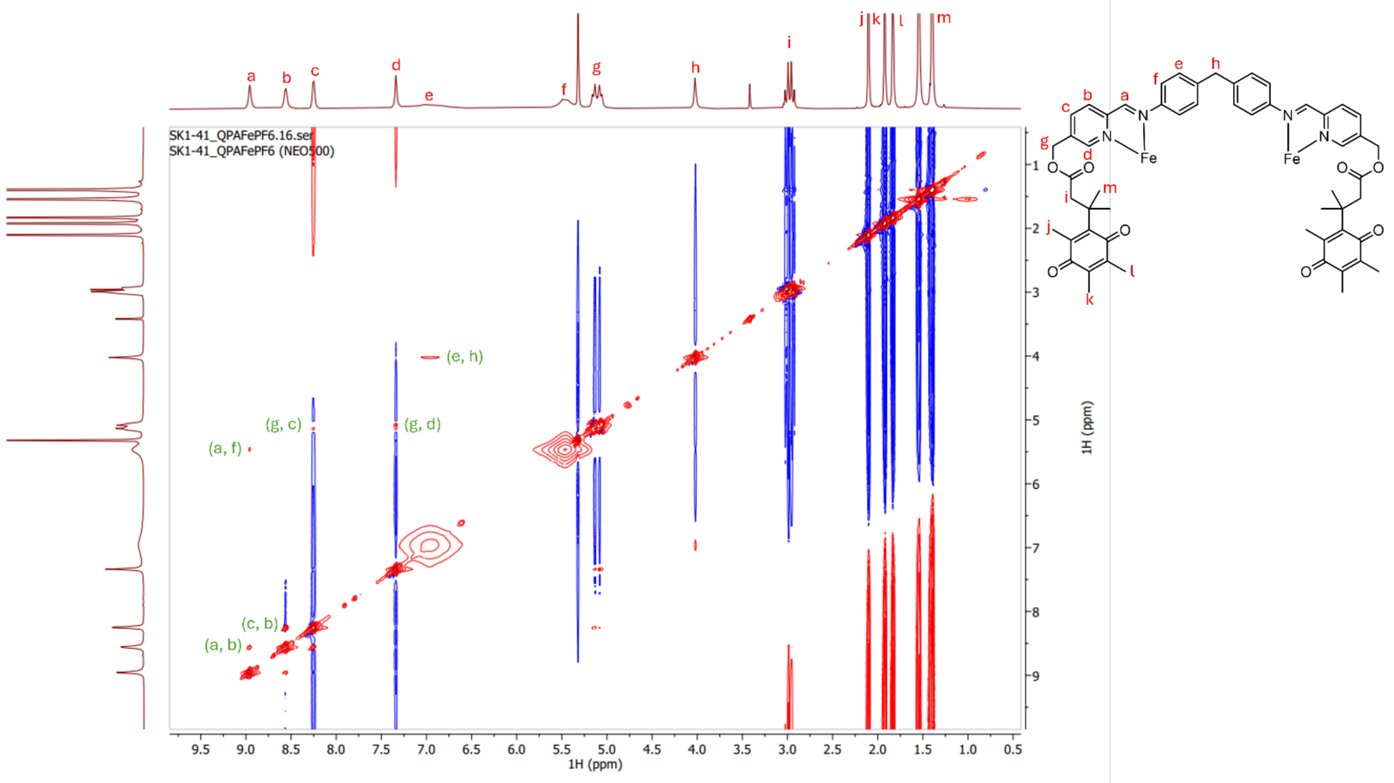

Figure S23: NOESY (500 MHz) of $[Fe_2(L2)_3](PF_6)_4$ in CD_2Cl_2.

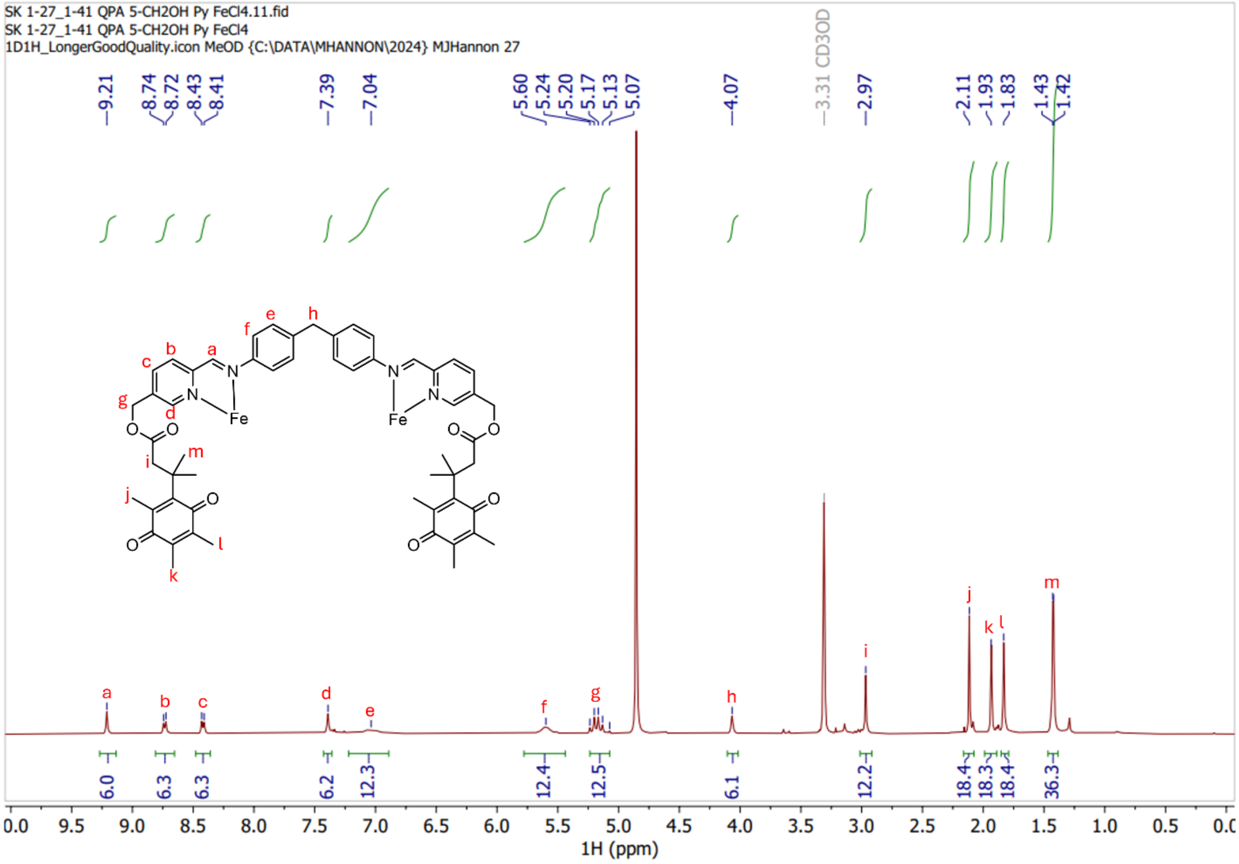

Figure S24: ^{1}H NMR (400 MHz) of $[Fe_2(L2)_3]Cl_4$ in Methanol-d_4.

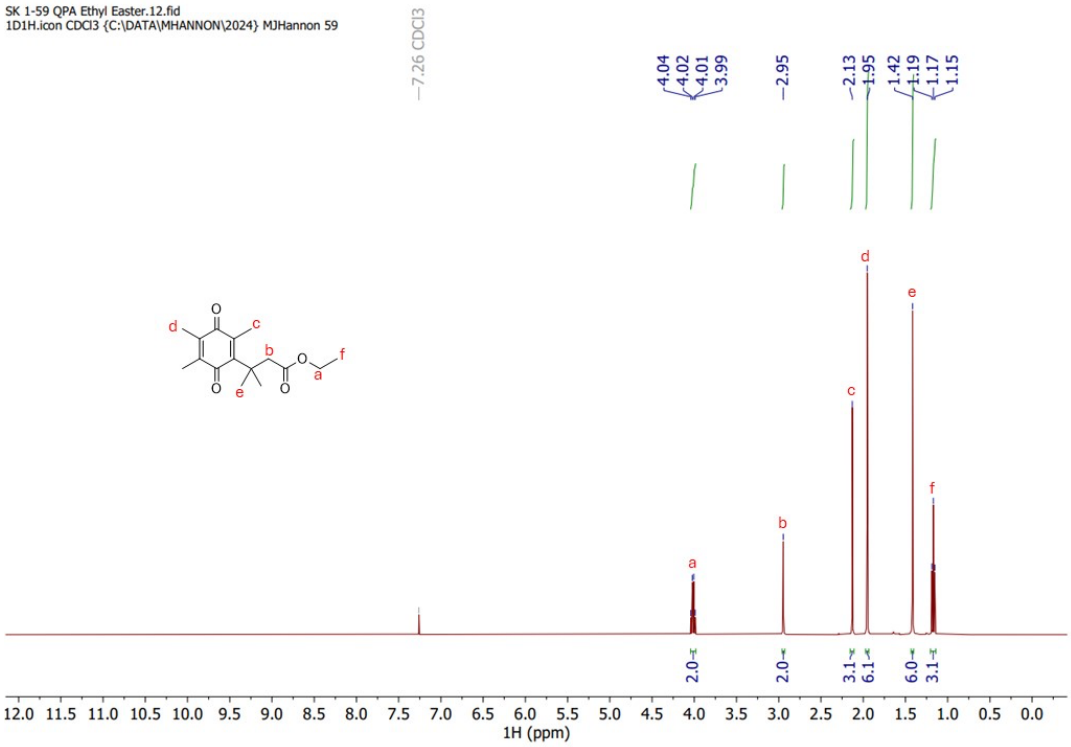

Figure S25: ^{1}H NMR (400 MHz) of QPA Et in $CDCl_3$.

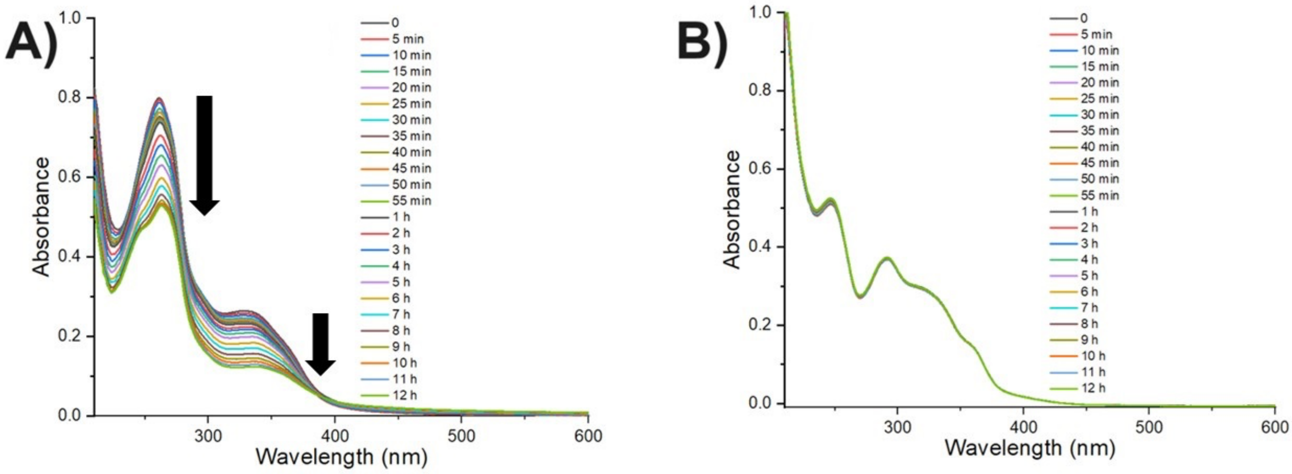

Figure S26: Stability study of $[Ni_2(L2)_3]Cl_4$ (A) and $[Ni_2(L1)_3]Cl_4$ (B) in 1X TA buffer pH 7.4 monitored by UV-Vis spectroscopy at 37 °C.

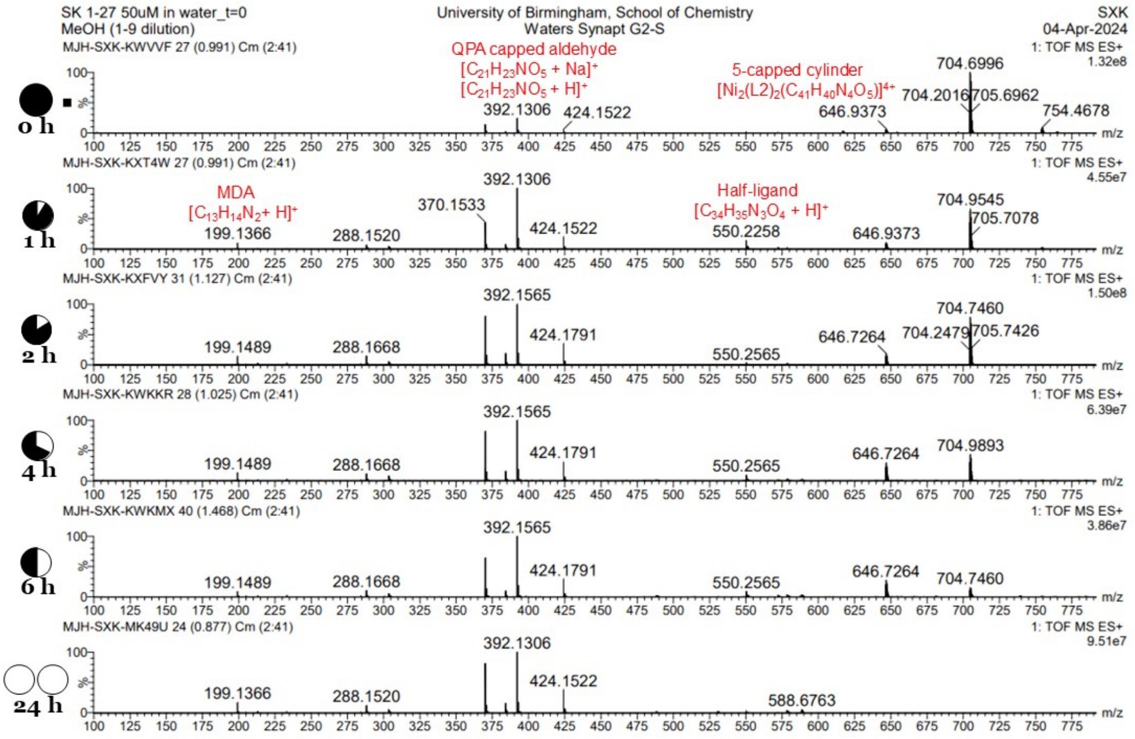

Figure S27: Stability study of $[Ni_2(L2)_3]Cl_4$ in water monitored by ESI-MS at 37 °C. m/z_{704} = $[Ni_2(L2)_3]^{4+}$; m/z_{646} = 5-capped cylinder, $[Ni_2(L2)_2(C_{41}H_{40}N_4O_5)]^{4+}$; m/z_{550} = half ligand, $[C_{34}H_{35}N_3O_4 + H]^+$; m/z_{392} = QPA capped aldehyde, $[C_{21}H_{23}NO_5 + Na]^+$; m/z_{370} = QPA capped aldehyde, $[C_{21}H_{23}NO_5 + H]^+$; m/z_{199} = MDA, $[C_{13}H_{14}N_2 + H]^+$.

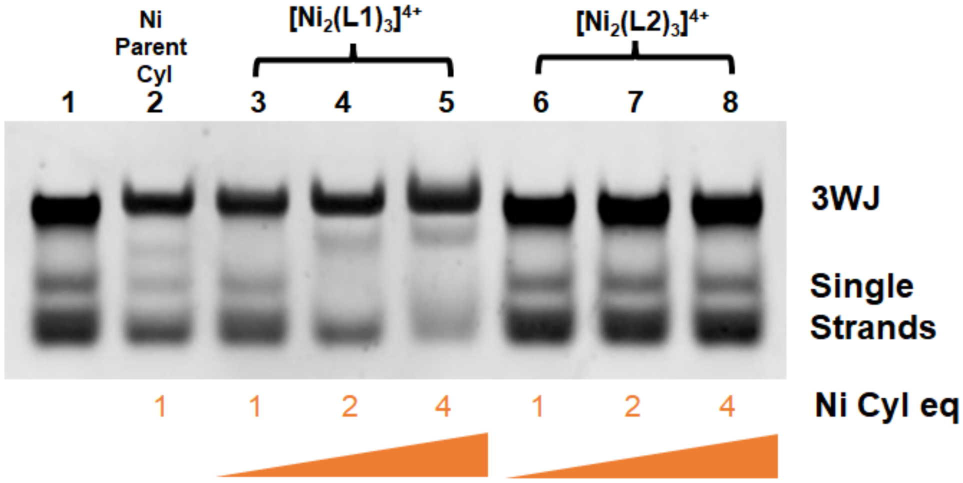

Figure S28: PAGE gel depicting the preformed room temperature stable DNA 3WJ binding behaviour of Ni-cylinders. 18-mer DNA oligonucleotide single strands were used here where S4: (5′-GTGGCGAGAGCGACGATC-3′), S5: (5′-GATCGTCGCAGAGTTGAC-3′) & S6: (5′-GTCAACTCTTCTCGCCAC-3′) and stained by SYBR™ Gold Nucleic Acid Gel Stain.

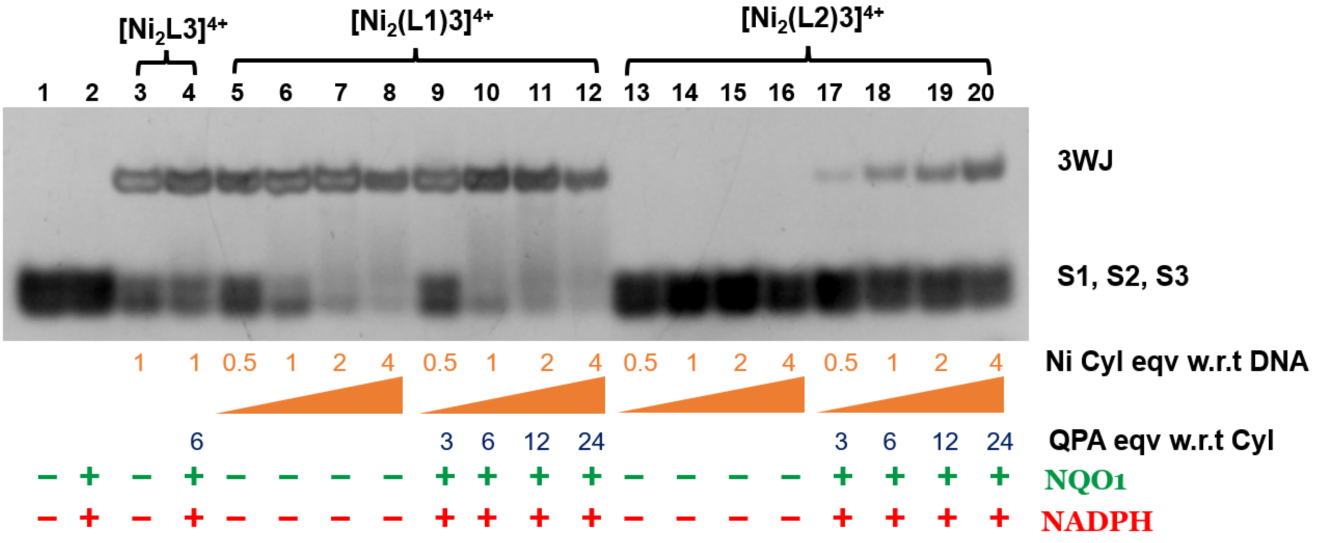

Figure S29: PAGE gel showing the DNA 3WJ binding of uncapped Ni-cylinder, $[Ni_2(L1)_3]^{4+}$ (lanes 5 – 8). Capped cylinder, $[Ni_2(L2)_3]^{4+}$, showing no 3WJ binding (lanes 13 – 16) in absence, and 3WJ binding in presence of NQO1/NADPH (lanes 17 – 20). Lanes 1 – 2 are negative control (untreated) and lane 3 is a positive control (Ni parent cylinder, $[Ni_2L_3]Cl_4$). Lanes 4, 9 – 12 & 17 – 20 are treated with 6 equivalents of QPA Et with respect to the individual cylinders. 14-mer DNA oligonucleotide single strands used were S1: 5′ -CGGAACGGCACTCG-3′ , S2: 5′ -CGAGTGCAGCGTGG-3′ & S3: 5′ -CCACGCTCGTTCCG-3′ and the gel was stained by SYBR™ Gold Nucleic Acid Gel Stain. NQO1 concentration in the sample mixture was 50 µg/mL and NADPH was used at a 100-fold molar equivalent with respect to the concentration of metallo-cylinder.

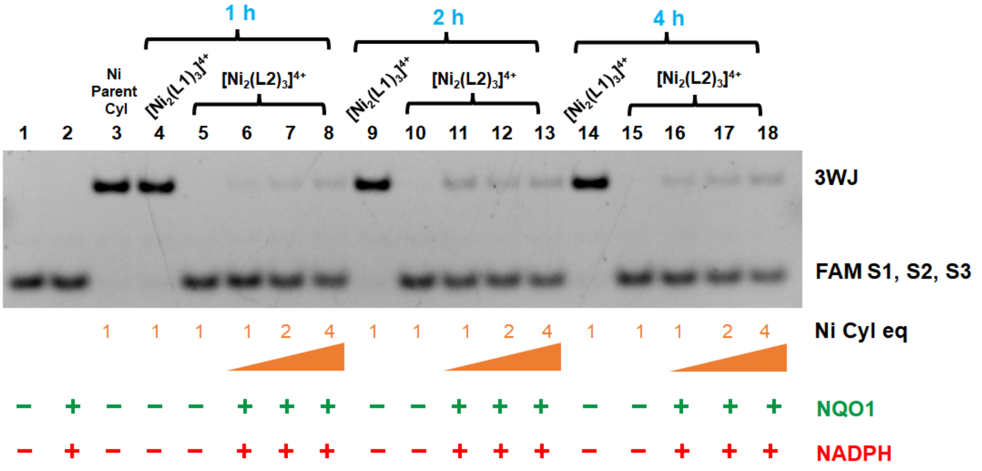

Figure S30: PAGE gel depicting the DNA 3WJ binding behaviour of capped cylinder, $[Ni_2(L2)_3]Cl_4$, at various incubation time points (1 h, lanes 6 – 8; 2 h, lanes 11 – 13 & 4 h, lanes 16 – 18). Lanes 1 – 2 negative control and lane 3 as the positive control (Ni parent cylinder, $[Ni_2L_3]Cl_4$). FAM labelled 14-mer DNA oligonucleotide single strand S1 (5′ 6-FAM-CGGAACGGCACTCG-3′), unlabeled 14-mer DNA oligonucleotide single strands S2 (5′- CGAGTGCAGCGTGG-3′) & S3 (5′-CCACGCTCGTTCCG-3′) were used and not stained with SYBR™ Gold Nucleic Acid Gel Stain. NQO1 concentration in the sample mixture was 50 µg/mL and NADPH was used at a 100-fold molar equivalent with respect to the concentration of metallo-cylinder.

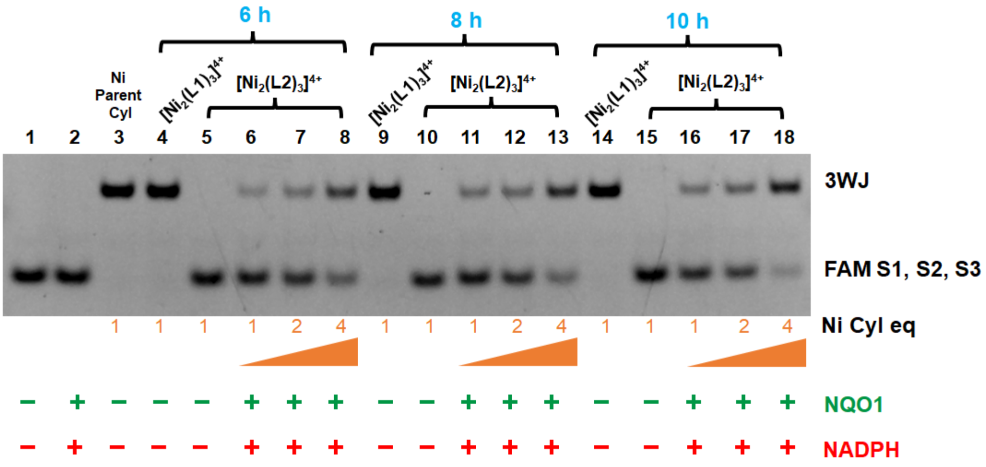

Figure S31: PAGE gel depicting the DNA 3WJ binding behaviour of capped cylinder, $[Ni_2(L2)_3]Cl_4$, at various incubation time points (6 h, lanes 6 – 8; 8 h, lanes 11 – 13 & 10 h, lanes 16 – 18). Lanes 1 – 2 negative control and lane 3 as the positive control (Ni parent cylinder, $[Ni_2L_3]Cl_4$). FAM labelled 14-mer DNA oligonucleotide single strand S1 (5′ 6-FAM-CGGAACGGCACTCG-3′), unlabeled 14-mer DNA oligonucleotide single strands S2 (5′- CGAGTGCAGCGTGG-3′) & S3 (5′-CCACGCTCGTTCCG-3′) were used and not stained with SYBR™ Gold Nucleic Acid Gel Stain. NQO1 concentration in the sample mixture was 50 µg/mL and NADPH was used at a 100-fold molar equivalent with respect to the concentration of metallo-cylinder.

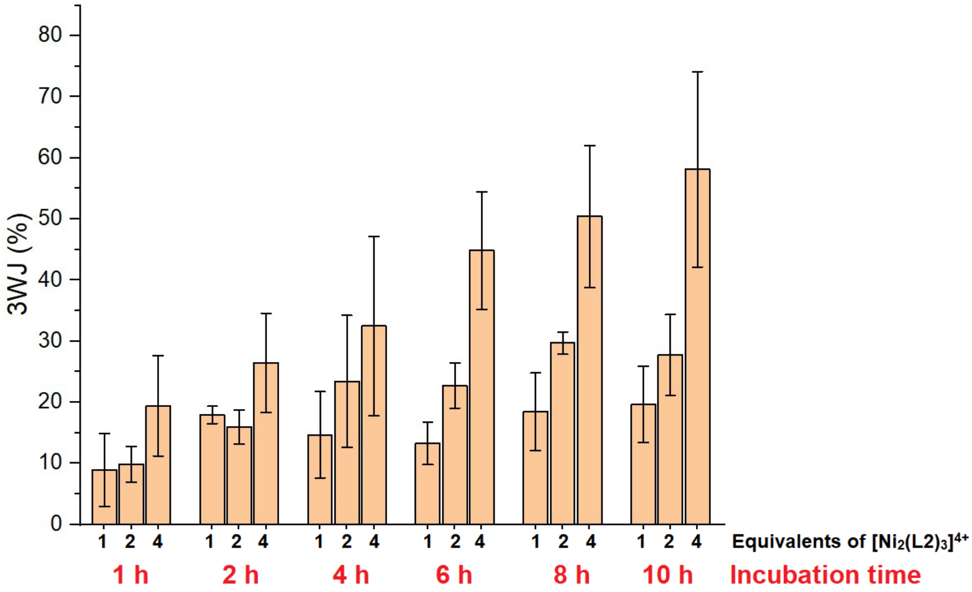

Figure S32: Variation in the intensity of DNA 3WJ bands with different incubation periods and increasing concentrations in capped cylinder, $[Ni_2(L2)_3]Cl_4$. 3WJ (%) presented here is the average of two independent experiments. Error bar represents standard error of the mean, n=2.

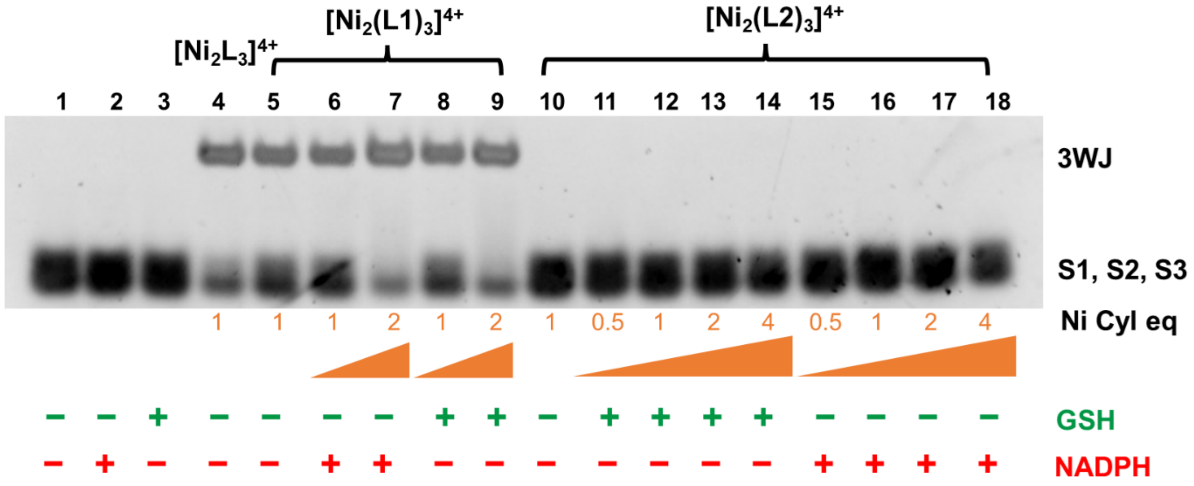

Figure S33: PAGE gel illustrating the effect of NADPH (lanes 15 – 18) and GSH (lanes 11 – 14), respectively, on the DNA 3WJ binding of capped cylinder, $[Ni_2(L2)_3]^{4+}$. Lanes 1 – 2 negative control and lane 3 as the positive control (Ni parent cylinder, $[Ni_2L_3]Cl_4$). 14-mer DNA oligonucleotide single strands were used here where S1: 5′-CGGAACGGCACTCG-3′, S2: 5′-CGAGTGCAGCGTGG-3′ & S3: 5′-CCACGCTCGTTCCG-3′ and stained by SYBR™ Gold Nucleic Acid Gel Stain. NADPH (or GSH) was used at a 100-fold molar equivalent with respect to the concentration of metallo-cylinder.

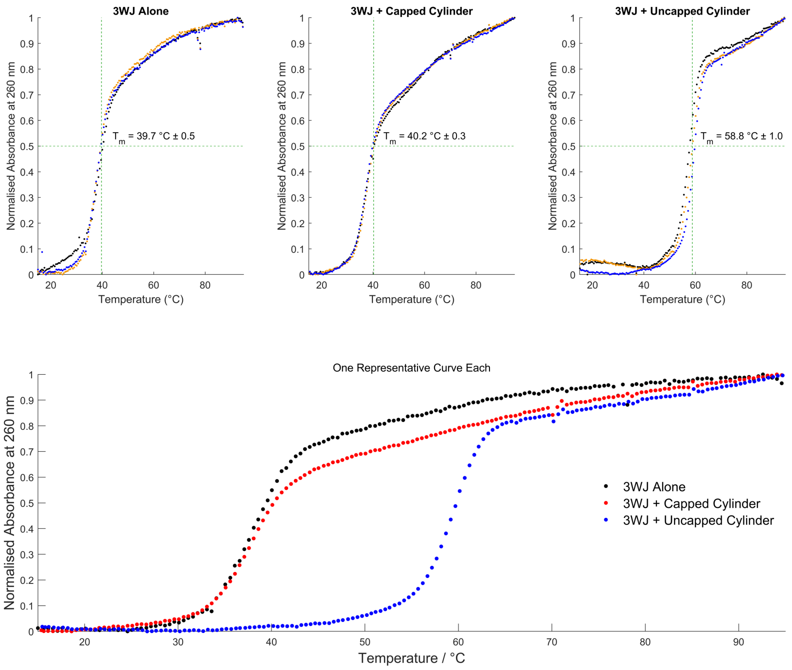

Figure S34: UV melting curves of DNA 3WJ in presence and absence of capped and uncapped cylinders respectively.

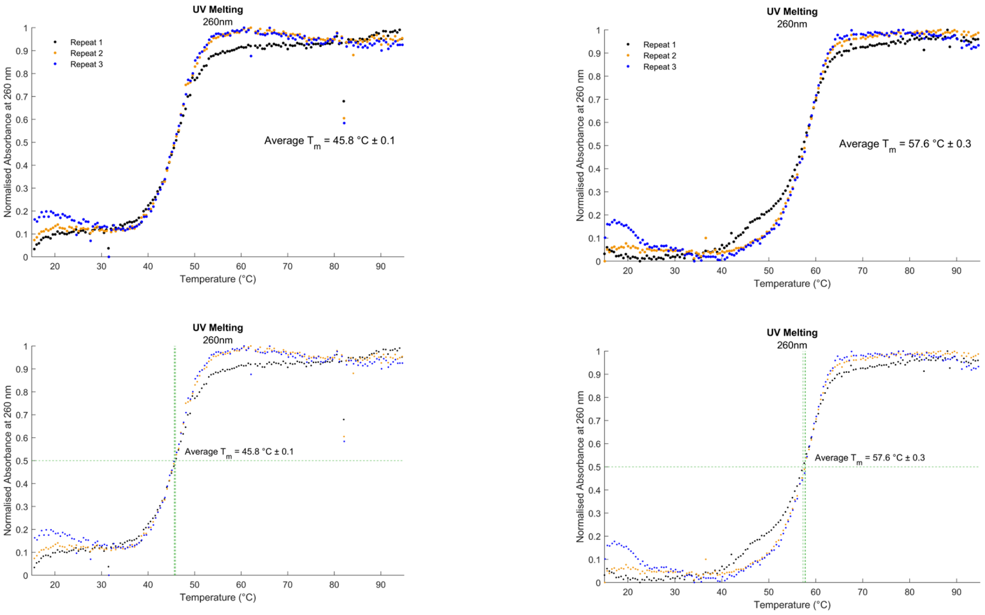

Figure S35: UV melting curves of RNA 3WJ in presence (right) and absence (left) of uncapped cylinder.

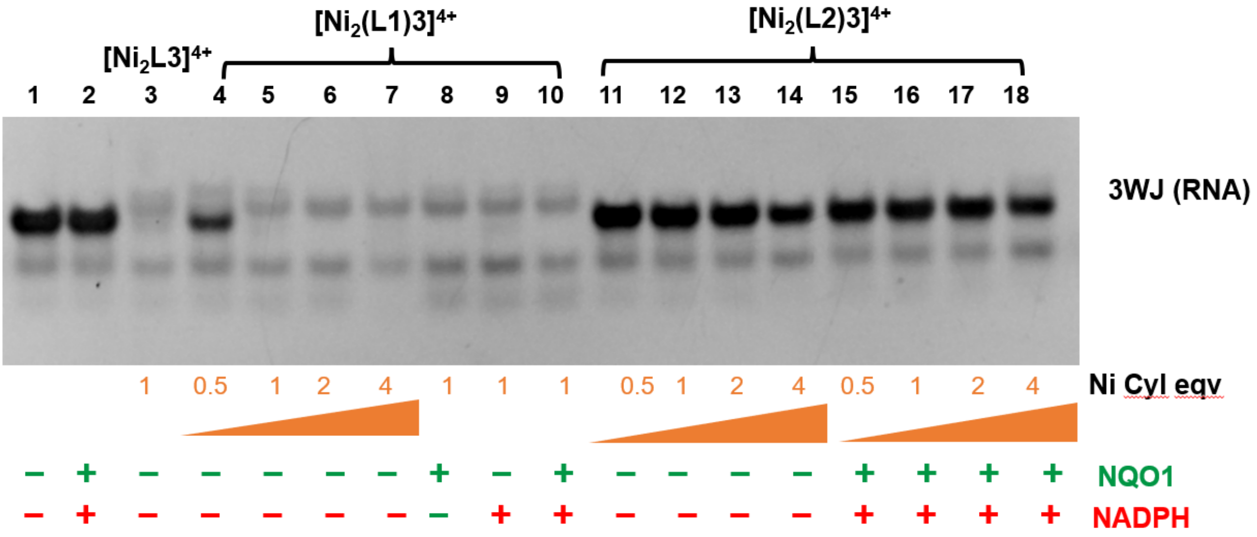

Figure S36: PAGE gel showing the RNA 3WJ binding of uncapped Ni-cylinder, $[Ni_2(L1)_3]^{4+}$ (lanes 4 – 7). Capped cylinder, $[Ni_2(L2)_3]^{4+}$, showing no RNA 3WJ binding (lanes 11 – 14) in absence, and 3WJ binding in presence of NQO1/NADPH (lanes 15 – 18). Lanes 1 – 2 are negative control (untreated) and lane 3 is a positive control (Ni parent cylinder, $[Ni_2L_3]Cl_4$). The gel was stained by SYBR™ Gold Nucleic Acid Gel Stain. NQO1 concentration in the sample mixture was 50 µg/mL and NADPH was used at a 100-fold molar equivalent with respect to the concentration of metallo-cylinder.

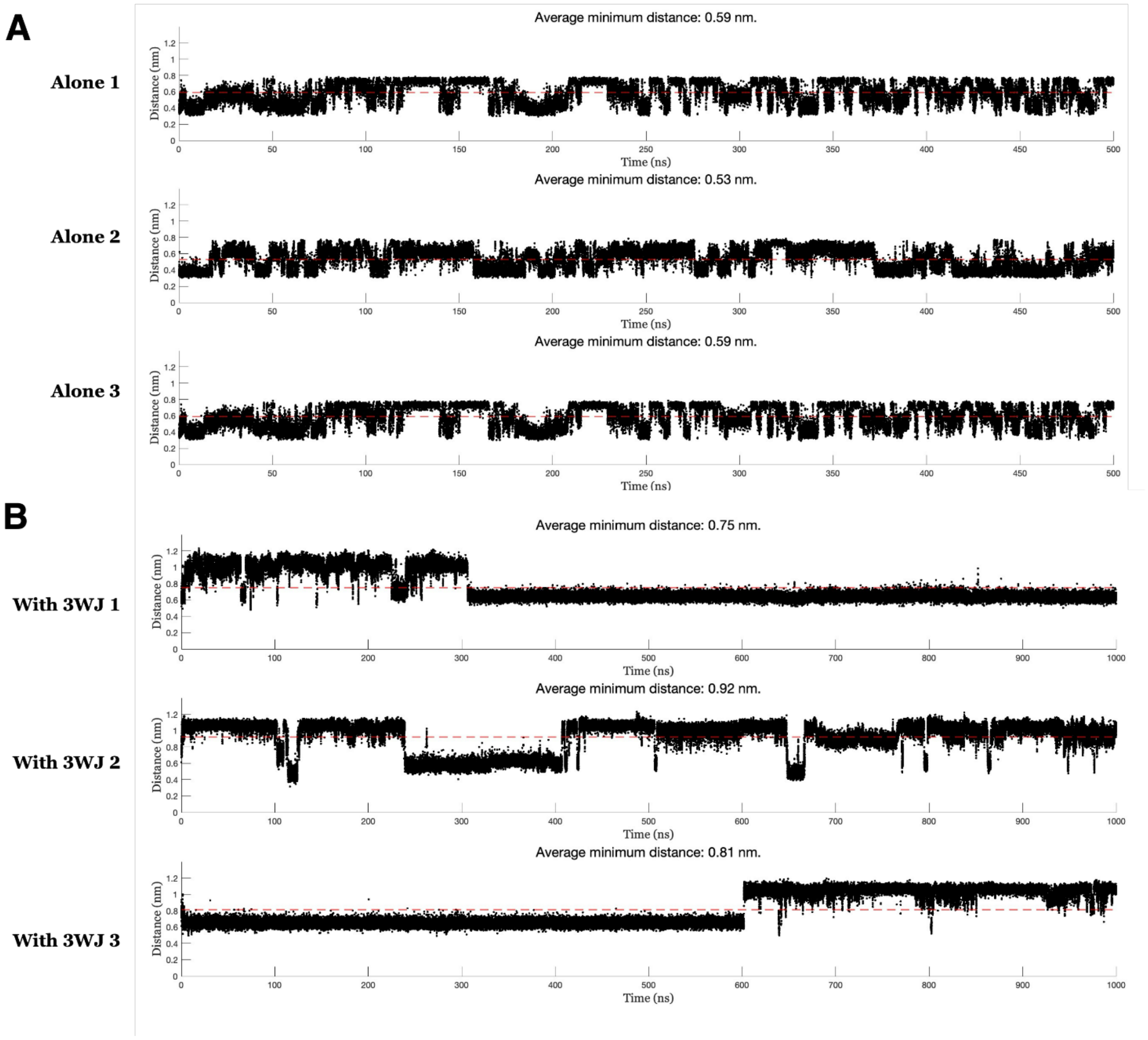

Figure S37: Distance plots showing the minimum distance between any central CH_2 (in the cylinder core) and any quinone (defined as any non-hydrogen atom in or attached directly to the quinone ring) in each simulation of the capped cylinder alone (A) and the capped cylinder with 3WJ DNA (B). The red dashed line shows the average minimum distance across each simulation. Minimum distances were calculated using the GROMACS mindist function and plotted in MATLAB.

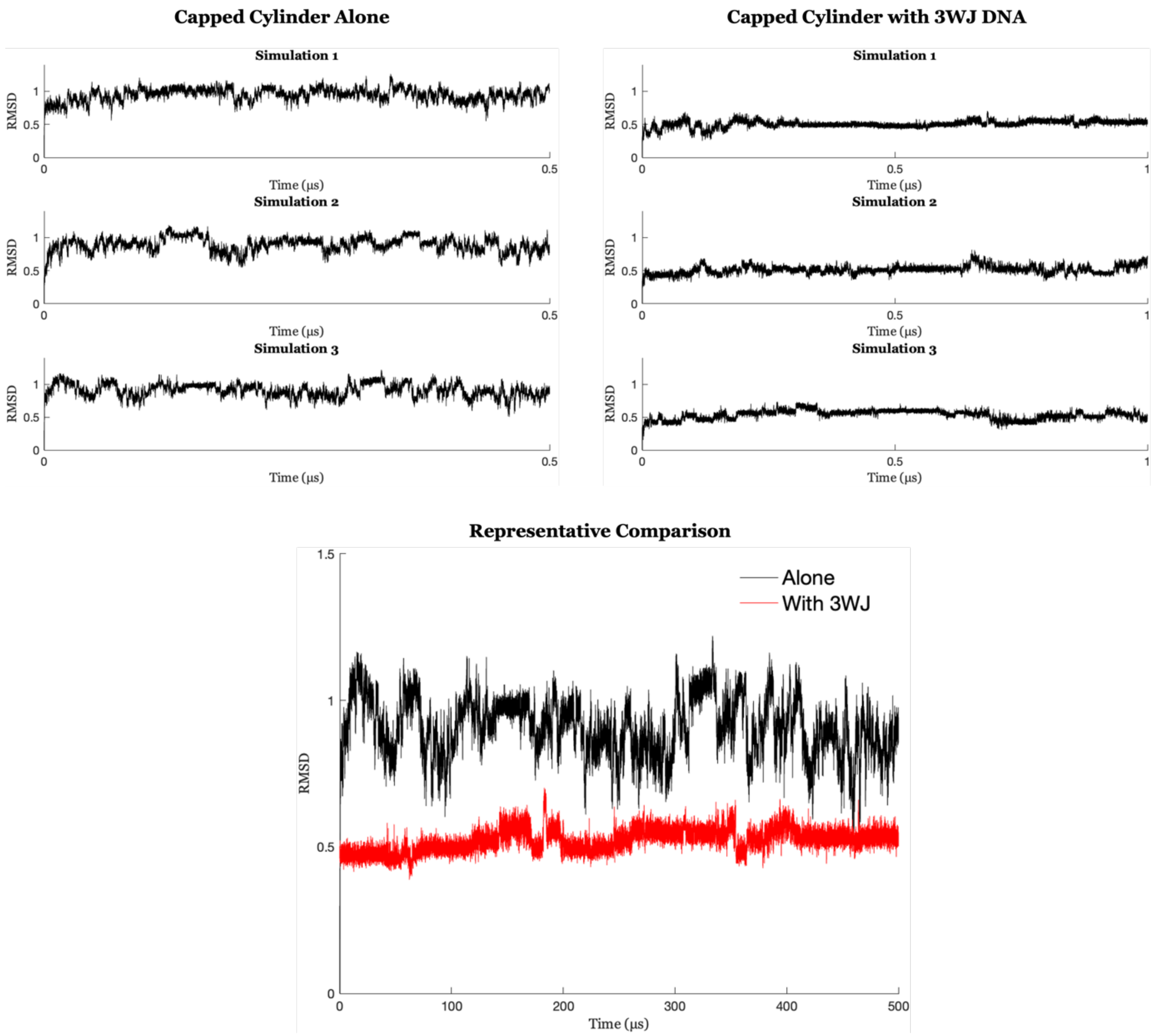

Figure S38: RMSD plots of the QPA caps (calculated by selecting all non-hydrogen atoms in or directly attached to the quinone rings) of the capped cylinder in MD simulations with no DNA (top left) and simulations with 3WJ DNA (top right). A representative comparison plot is also shown (bottom). RMSD plots show the change in coordinates of selected atoms compared to the first frame of the simulation. These RMSD plots show that the QPA groups exhibit much more freedom of movement when the cylinder is not constrained by the DNA. RMSD was calculated using the GROMACS rms function and plotted in MATLAB.

A

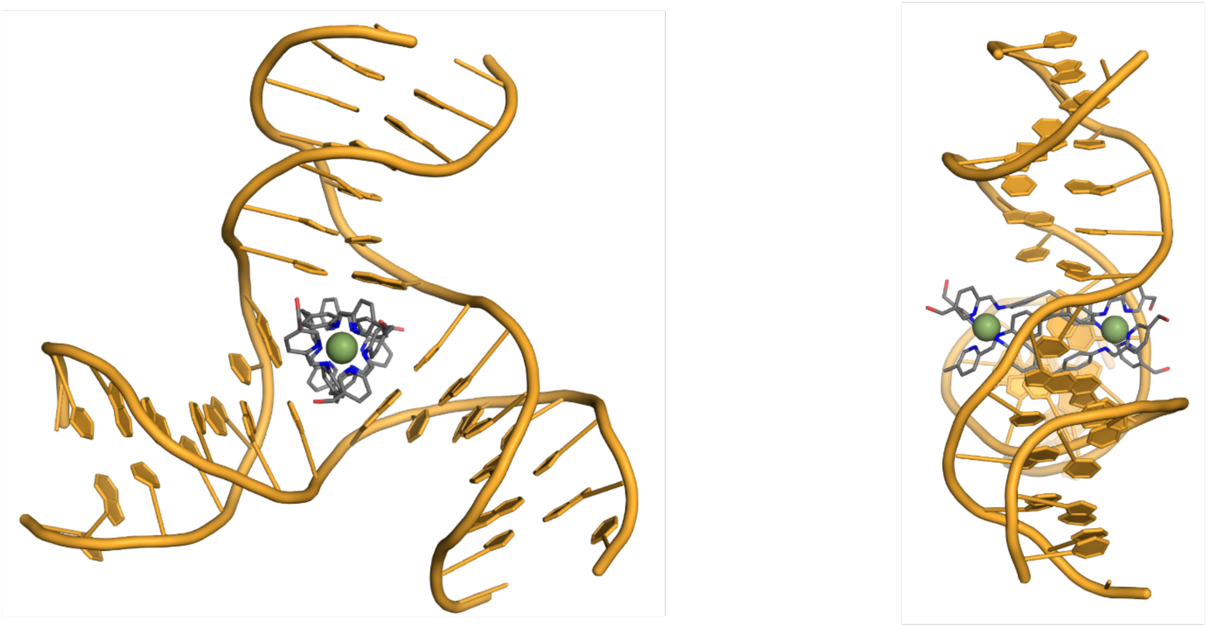

B

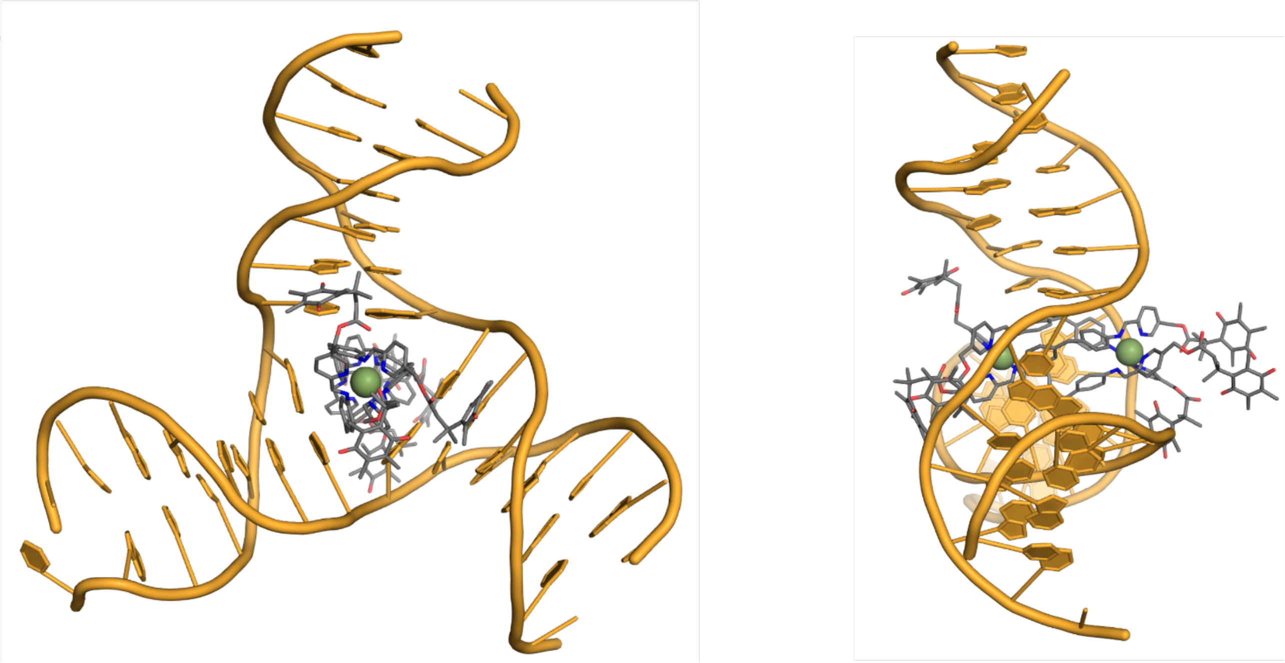

Figure S39: Molecular dynamics snapshots of A) the uncapped cylinder residing in the 3WJ and B) the capped cylinder residing in the 3WJ.

Table S1: Cytotoxicity data of capped & uncapped cylinders and cisplatin

Compounds	IC_{50} ± SD (μM)[a]		
	A549	A549/Dicoumarol$_{25μM}$	A549/Dicoumarol$_{50μM}$
$[Ni_2(L2)_3]Cl_4$	3.0 ± 0.6	14.9 ± 3.1[b]	26.1 ± 2.7[c]
$[Ni_2(L1)_3]Cl_4$	>100	>100[b]	>100[c]
Cisplatin	9.1 ± 1.5	21.3 ± 4.9[b]	29.2 ± 1.7[c]

[a]SD = Standard deviation, IC_{50} values were calculated by nonlinear curve fitting in dose response inhibition – variable slope model using Graph pad prism, the data presented have significance (p value less than 0.05 or better); [b]Cells were co-treated with target compound as well as 25 μM of dicoumarol; [c]Cells were co-treated with target compound as well as 50 μM of dicoumarol.
Note that the compound $[Ni_2(L1)_3]Cl_4$ is not well uptaken into cells as shown in Figure S40

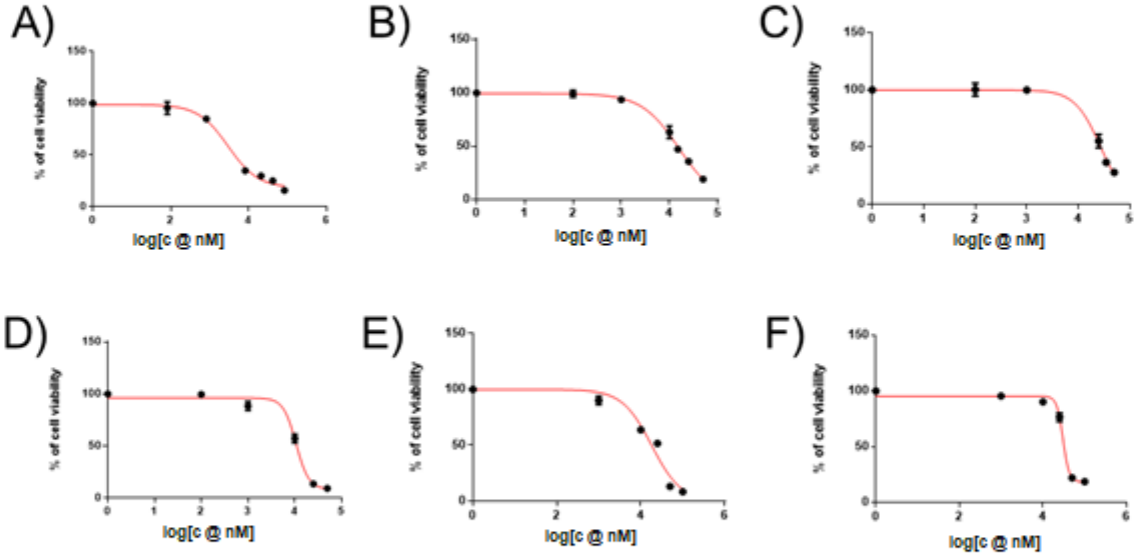

Figure S40: Plots of cell viability (%) vs. log of concentrations (in nM) of $[Ni_2(L2)_3]Cl_4$ (A), $[Ni_2(L2)_3]Cl_4$ + 25 μM dicoumarol (B), $[Ni_2(L2)_3]Cl_4$ + 50 μM dicoumarol (C), cisplatin (D), cisplatin + 25 μM dicoumarol (E) and cisplatin + 50 μM dicoumarol (F) against A549 cell line after incubation for 72 h determined from MTT assays. 100% cell viability represents cell growth in presence of the appropriate level of dicoumarol but no cylinder. The dicoumarol alone has no significant effect on cell growth as shown in Table S2.The plots provided are for one independent experiment out of the three independent experiments.

Table S2: Comparison of cell viability of dicoumarol (25 & 50 µM respectively) treated cells with respect to untreated cells

Mean Absorbance of Formazan @ 595 nm ± SD[a]		
Control	$Dicoumarol_{25\mu M}$	$Dicoumarol_{50\mu M}$
1.79 ± 0.56	1.45 ± 0.45	1.15 ± 0.49

[a]SD = Standard deviation, Statistical analysis was performed by a one-way ANOVA followed by a Dunnett's post hoc t-test (n =3 or higher). The data shows a minor reduction in cell viability in presence of dicoumarol which is not statistically significant.

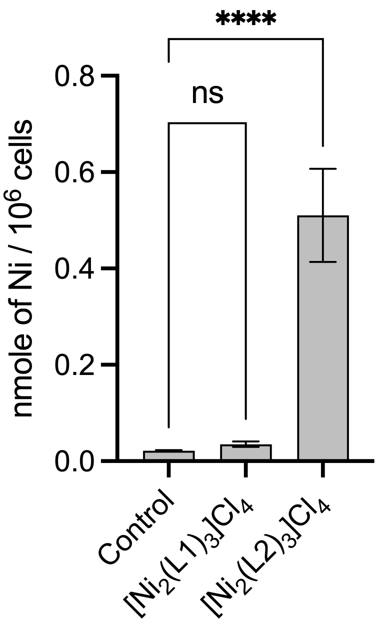

Figure S41: Graph shows the difference in cellular accumulation of capped ($[Ni_2(L2)_3]Cl_4$) and uncapped cylinder ($[Ni_2(L1)_3]Cl_4$) after treatment with same concentration of both the cylinders respectively for 24 h. Error bars are standard deviations. Symbols **** ($p<0.0001$) and ns = not significant. Test is Ordinary one-way ANOVA followed by a post-hoc Dunnett T-test (n=4 or higher).

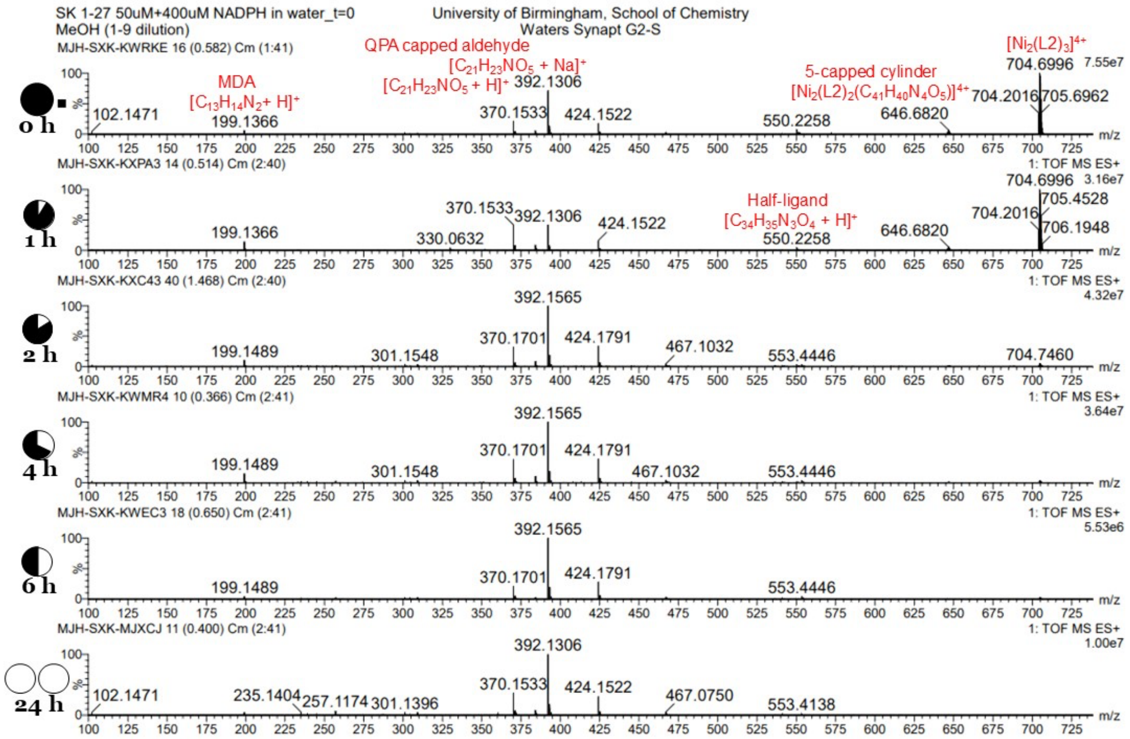

Figure S42: Stability study of a mixture of $[Ni_2(L2)_3]Cl_4$ and NADPH (8 eqv) in water monitored by ESI-MS at 37 °C. m/z_{704} = $[Ni_2(L2)_3]^{4+}$; m/z_{646} = 5-capped cylinder, $[Ni_2(L2)_2(C_{41}H_{40}N_4O_5)]^{4+}$; m/z_{550} = half ligand, $[C_{34}H_{35}N_3O_4 + H]^+$; m/z_{392} = QPA capped aldehyde, $[C_{21}H_{23}NO_5 + Na]^+$; m/z_{370} = QPA capped aldehyde, $[C_{21}H_{23}NO_5 + H]^+$; m/z_{199} = MDA, $[C_{13}H_{14}N_2 + H]^+$.

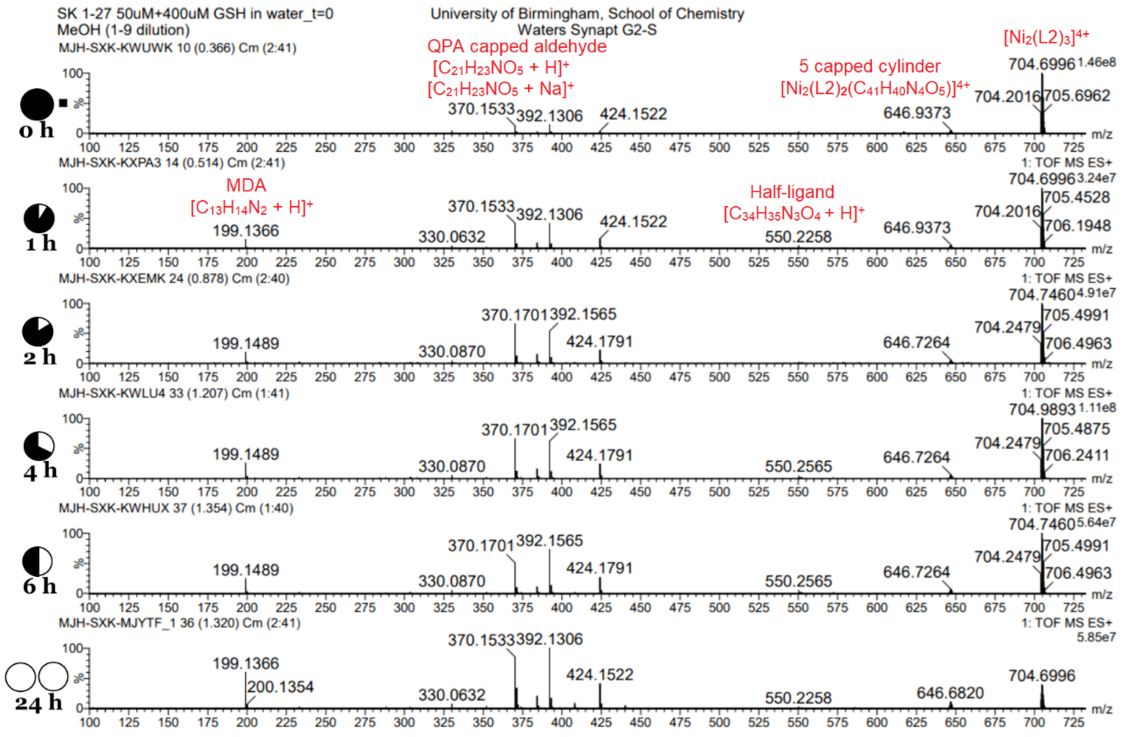

Figure S43: Stability study of a mixture of $[Ni_2(L2)_3]Cl_4$ and GSH (8 eqv) in water monitored by ESI-MS at 37 °C. m/z_{704} = $[Ni_2(L2)_3]^{4+}$; m/z_{646} = 5-capped cylinder, $[Ni_2(L2)_2(C_{41}H_{40}N_4O_5)]^{4+}$; m/z_{550} = half ligand, $[C_{34}H_{35}N_3O_4 + H]^+$; m/z_{392} = QPA capped aldehyde, $[C_{21}H_{23}NO_5 + Na]^+$; m/z_{370} = QPA capped aldehyde, $[C_{21}H_{23}NO_5 + H]^+$; m/z_{199} = MDA, $[C_{13}H_{14}N_2 + H]^+$.

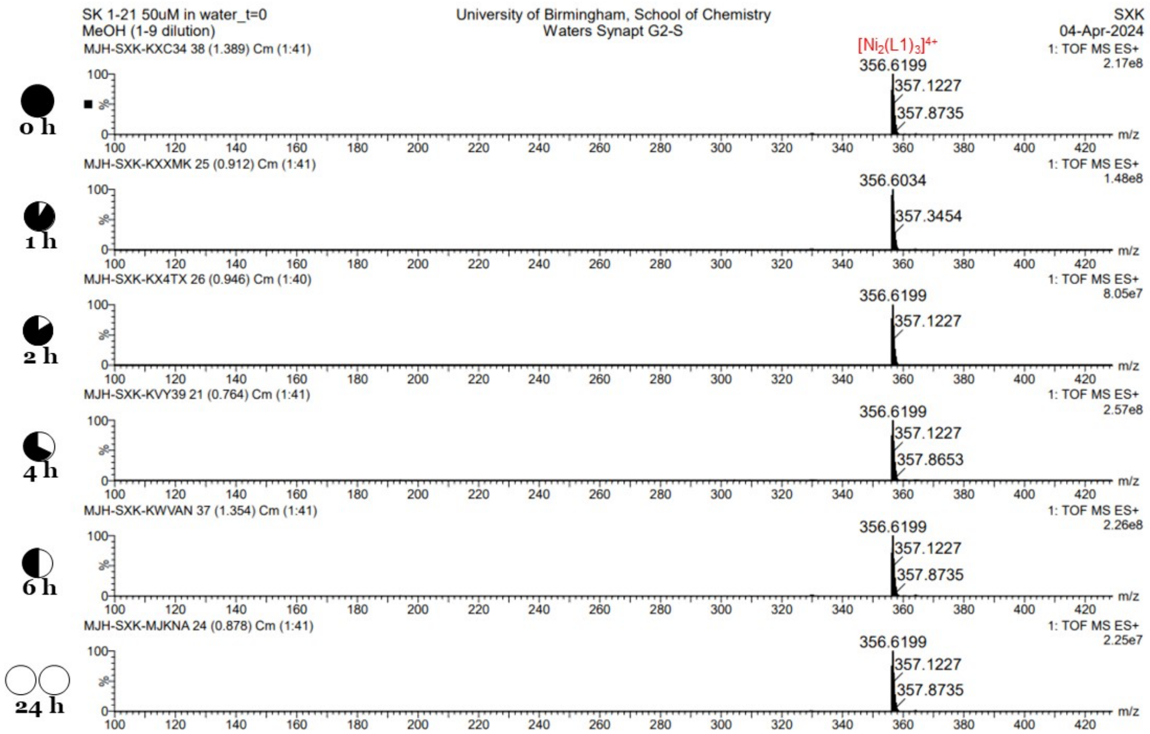

Figure S44: Stability study of $[Ni_2(L1)_3]Cl_4$ in water monitored by ESI-MS at 37 °C. m/z_{356} = $[Ni_2(L1)_3]^{4+}$.

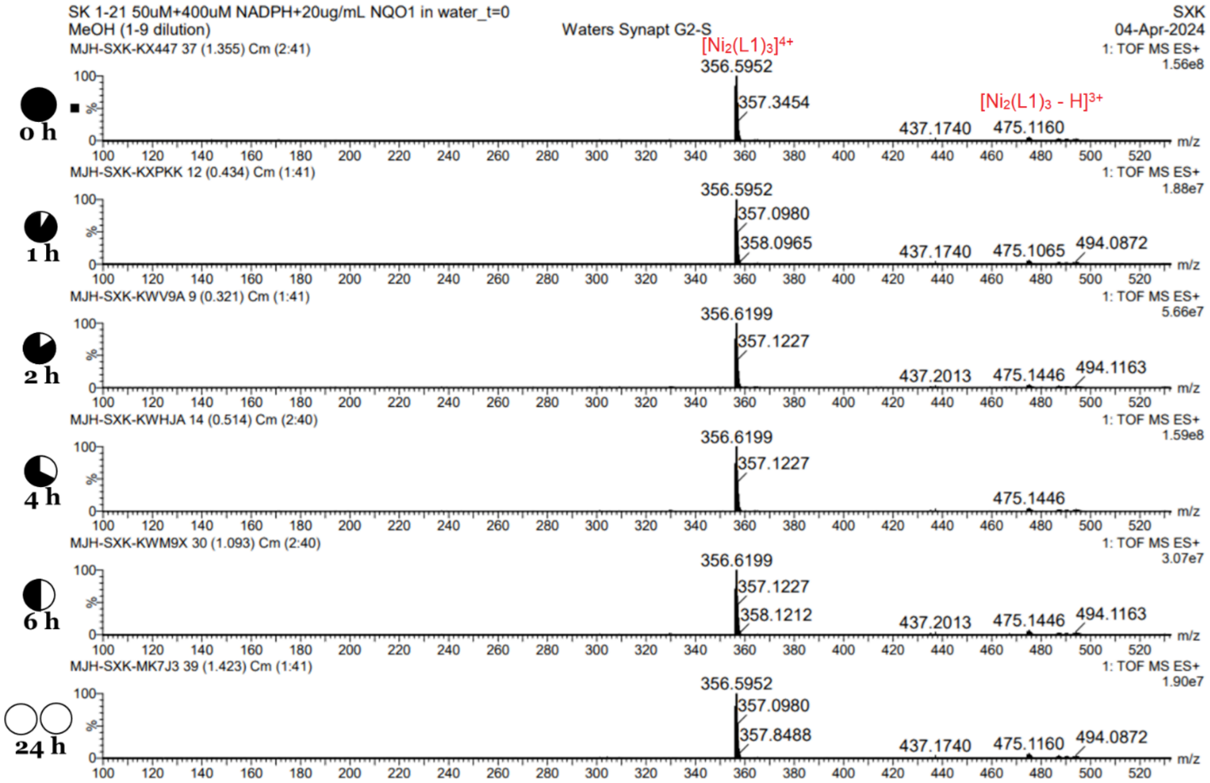

Figure S45: Stability study of a mixture of $[Ni_2(L1)_3]Cl_4$, NQO1 (20 µg/mL) and NADPH (8 eqv) in water monitored by ESI-MS at 37 °C. m/z_{356} = $[Ni_2(L1)_3]^{4+}$; m/z_{475} = $[Ni_2(L1)_3 - H]^{3+}$.

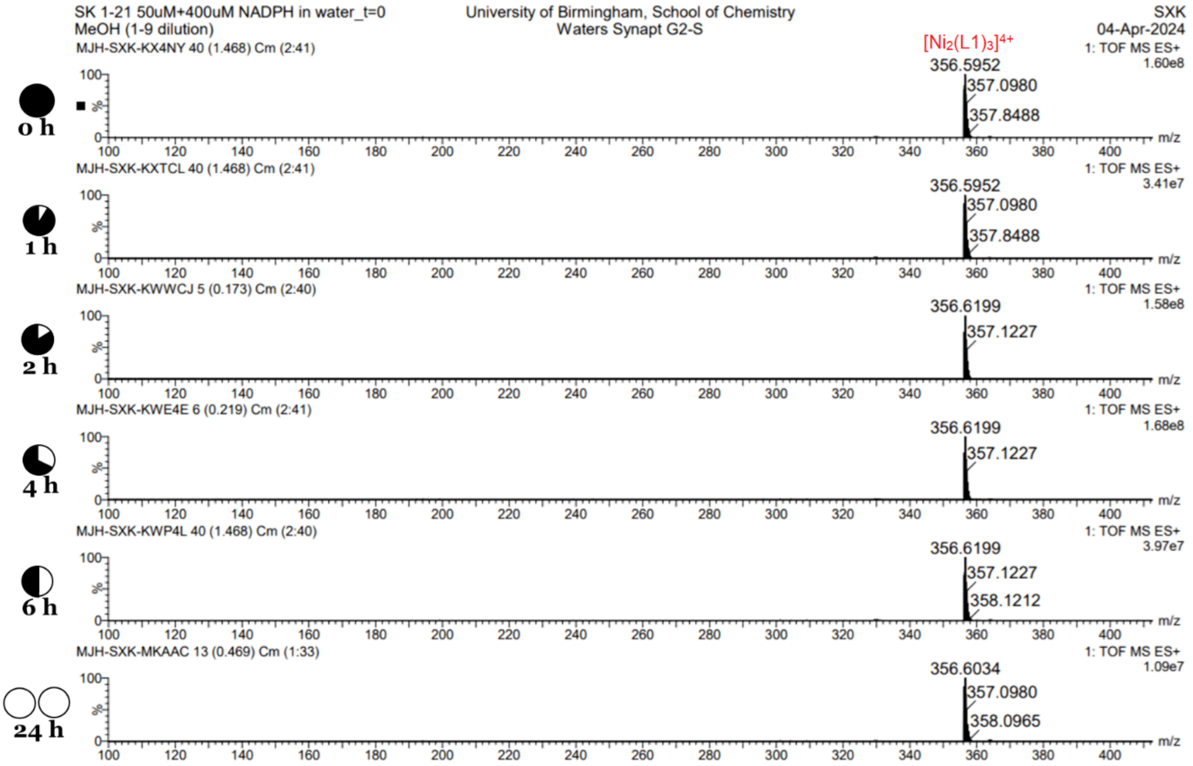

Figure S46: Stability study of a mixture of $[Ni_2(L1)_3]Cl_4$ and NADPH (8 eqv) in water monitored by ESI-MS at 37 °C. m/z_{356} = $[Ni_2(L1)_3]^{4+}$.

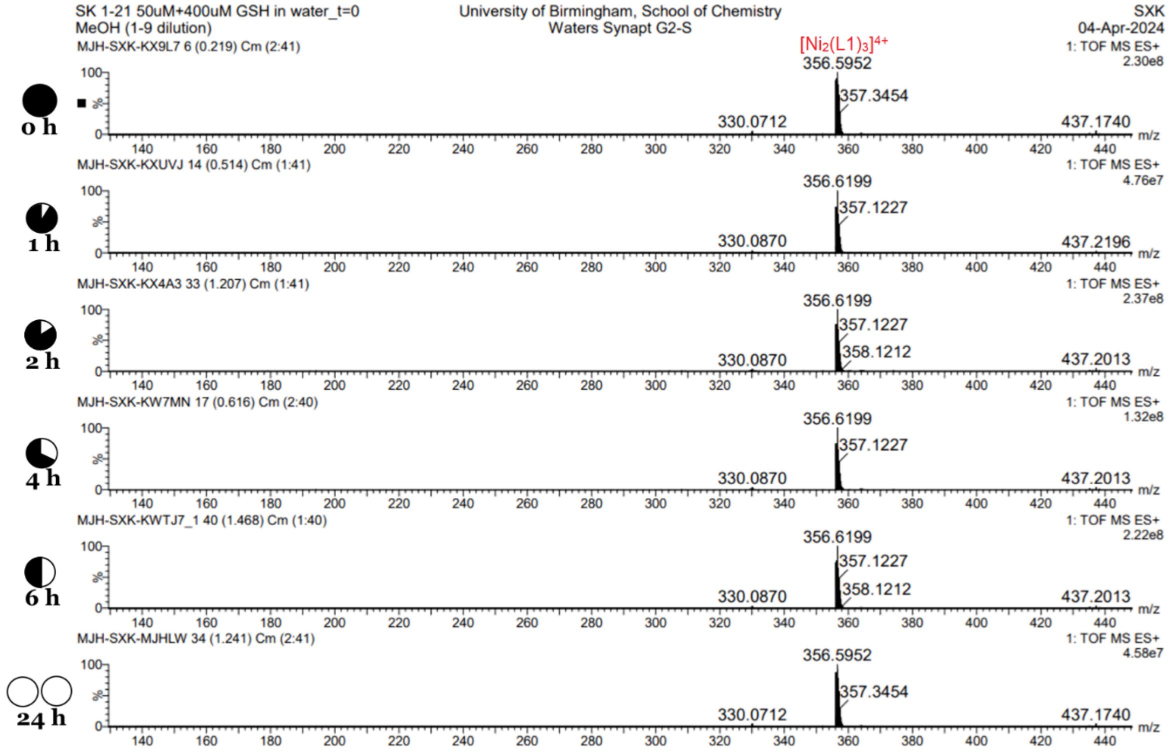

Figure S47: Stability study of a mixture of $[Ni_2(L1)_3]Cl_4$ and GSH (8 eqv) in water monitored by ESI-MS at 37 °C. m/z_{356} = $[Ni_2(L1)_3]^{4+}$.

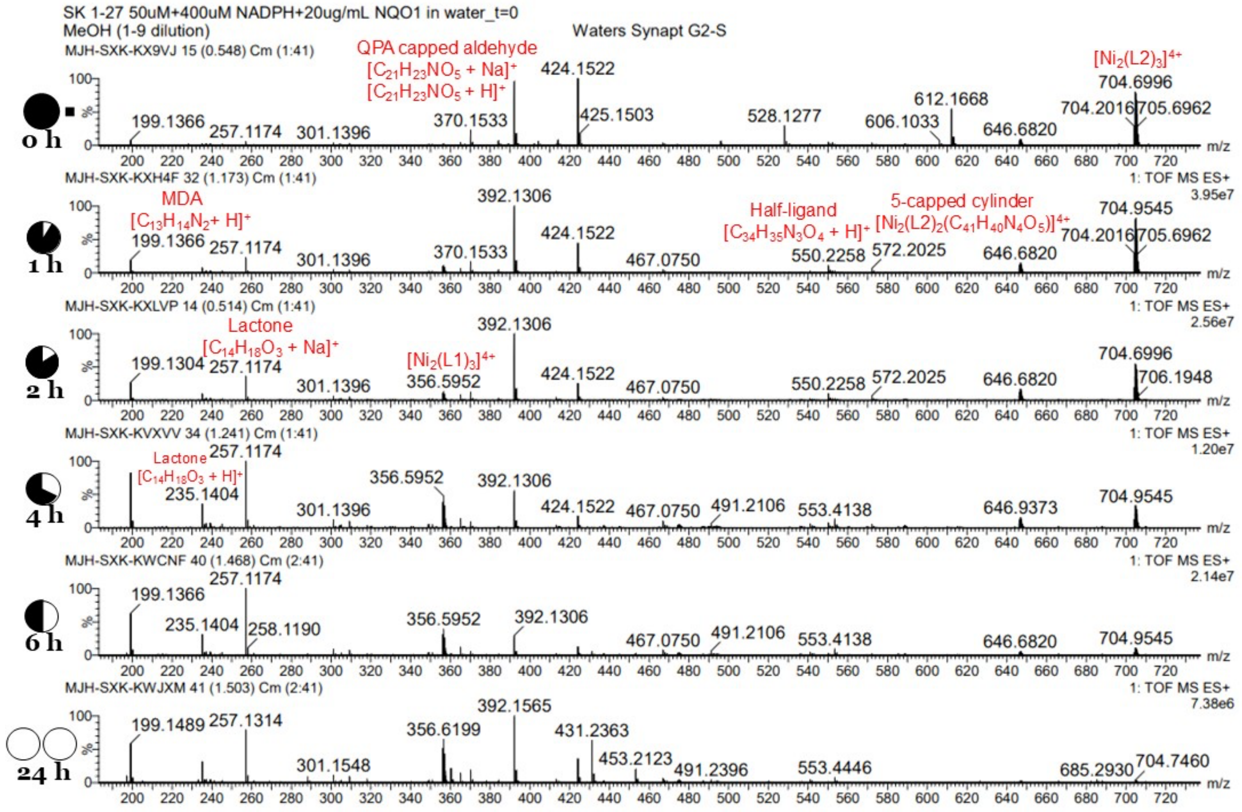

Figure S48: Stability study of a mixture of $[Ni_2(L2)_3]Cl_4$, NQO1 (20 µg/mL) and NADPH (8 eqv) in water monitored by ESI-MS at 37 °C. m/z_{704} = $[Ni_2(L2)_3]^{4+}$; m/z_{646} = 5-capped cylinder, $[Ni_2(L2)_2(C_{41}H_{40}N_4O_5)]^{4+}$; m/z_{550} = half ligand, $[C_{34}H_{35}N_3O_4 + H]^+$; m/z_{392} = QPA capped aldehyde, $[C_{21}H_{23}NO_5 + Na]^+$; m/z_{370} = QPA capped aldehyde, $[C_{21}H_{23}NO_5 + H]^+$; m/z_{356} = $[Ni_2(L1)_3]^{4+}$; m/z_{257} = $[C_{14}H_{18}O_3 + Na]^+$, lactone; m/z_{235} = $[C_{14}H_{18}O_3 + H]^+$, lactone; m/z_{199} = MDA, $[C_{13}H_{14}N_2 + H]^+$.

CD & LD Study with DNA duplex

Alongside DNA junction binding, cylinders can also potentially interact with genomic duplex DNA. Circular dichroism (CD)[1] titrations of uncapped $[Ni_2(L1)_3]^{4+}$ against calf thymus DNA (ctDNA) show that the B-DNA confirmation is retained (Figure 6A), while weak induced CD bands (300 – 400 nm) confirm cylinder binding. Flow linear dichroism (LD) titrations were also conducted, in which DNA is oriented through flow, and the orientation followed by measuring the absorption anisotropy between parallel and perpendicular light. They reveal a dramatic loss of DNA orientation on $[Ni_2(L1)_3]^{4+}$ binding, indicating that the complex causes coiling of DNA (Figure S48C). These behaviours are analogous to those of the unsubstituted $[M_2(L)_3]^{4+}$ complexes.[2] In contrast, LD spectra of $[Ni_2(L2)_3]^{4+}$ with ctDNA revealed only small changes in the DNA orientation and the absence of dramatic DNA coiling (Figures S48B, S48D). This serves once again to reinforce the link between junction-binding ability and DNA-coiling activity.

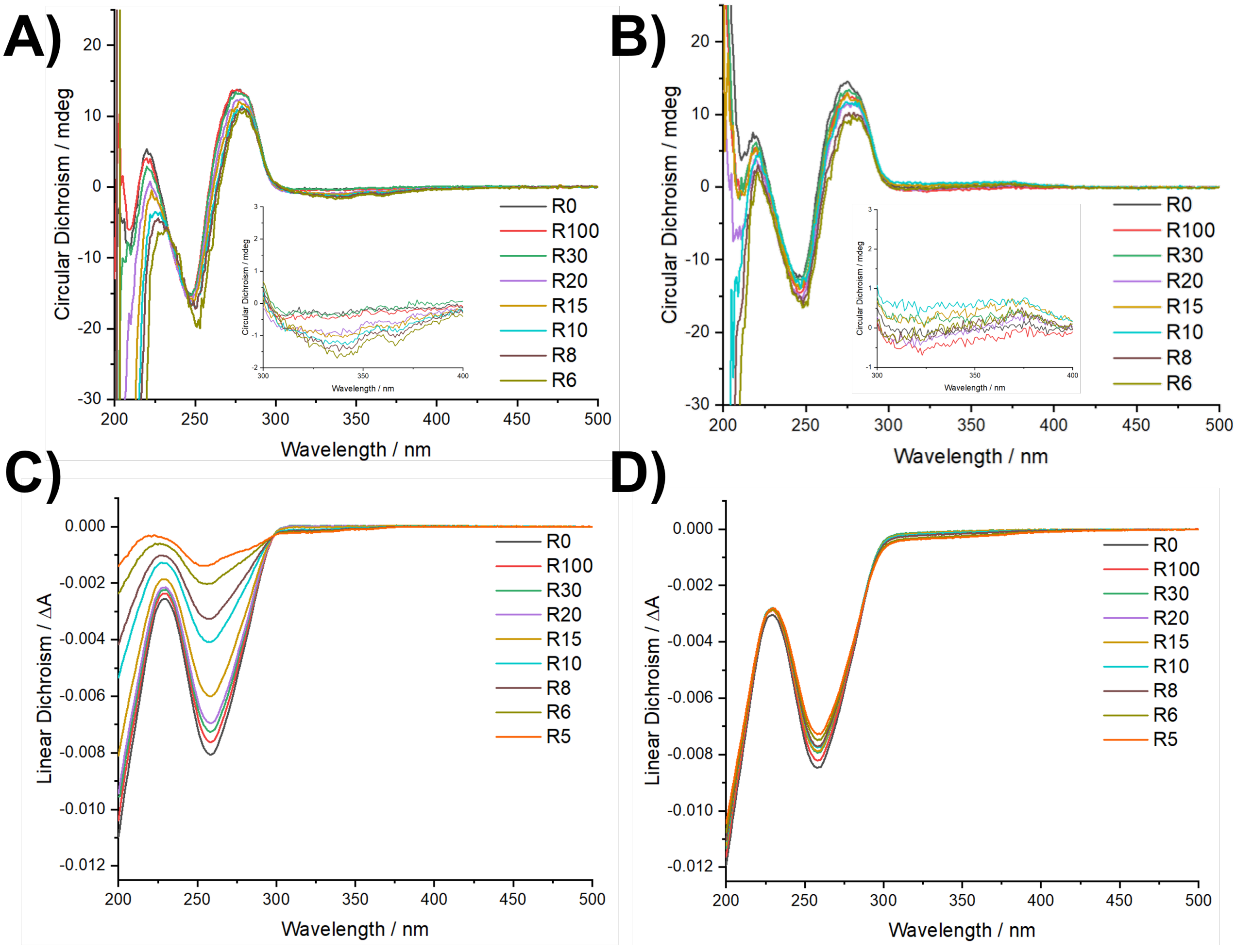

Figure S49: CD spectra (top) of ctDNA displaying the preservation of B-DNA conformation upon interaction with both the uncapped $[Ni_2(L1)_3]^{4+}$ (A) and capped $[Ni_2(L2)_3]^{4+}$ (B) cylinders. LD spectra (bottom) illustrate the large interaction of ctDNA with the uncapped cylinder (C) in contrast to the capped cylinder (D).

Experimental Procedures

Materials and Methods

All the chemicals and solvents were procured from verified commercial sources (Sigma-Aldrich and Fisher Scientific) and used without further purification. All the NMR data were recorded using either a Bruker AVIII 300 MHz, Bruker AVANCE NEO 400 MHz or Bruker AVANCE NEO 500 MHz NMR Spectrometer. All the data were processed on MestReNova (x64-14.0.0-23239) software, and the chemical shifts were described in parts per million (ppm). ^{1}H chemical shifts were referenced to the individual solvent residual peaks of respective NMR solvents used. All the ESI-MS data were recorded on a Waters SYNAPT-G2-S Mass Spectrometer. UV-vis absorbance data between 200-800 nm (1 nm bandwidth, 600 nm/min) using an Agilent Cary 60 UV-Vis Spectrophotometer. Stability kinetics of the complexes were measured on an Agilent Cary 60 UV-Vis Spectrophotometer with a peltier. Raw data was processed through OriginPro 2021 software. Elemental analyses were carried through using a Thermo Fisher Scientific FlashSmart™ Elemental Analyzer. PAGE gel running kits from Bio-Rad (PROTEAN II xi Cell and Mini-PROTEAN Cell) were used. Gels were imaged in AlphaImager™ UV transilluminator (Alpha Innotech) and processed with ImageJ (Fiji) software. ICP-MS data were recorded on an Agilent 7500ce. CD and LD data were recorded on a Chirascan+ spectrometer (Applied Photophysics limited).

Syntheses

Ligand L1 and its Ni and Fe complexes were first described in G. I. Pascu's PhD thesis[3] which also contains views of the crystal structures of these cations (G. I. Pascu, B. M. Kariuki, M. J. Hannon, Unpublished results). The synthesis of 5-Hydroxymethyl-2-pyridinecarboxaldehyde was reported previously by our group,[4] but we followed a different approach to synthesise the aldehyde which is given below.

Diethyl pyridine-2,5-dicarboxylate: Pyridine-2,5-dicarboxylic acid (1.0 g, 6 mmol) was suspended in absolute ethanol (8 mL) and sulphuric acid (1 mL) was added dropwise at ice-cold condition. The suspension dissolved immediately to give a light-yellow solution which was heated to reflux at 80 °C for 20 h. The reaction mixture was then cooled at room temperature and poured into 200 mL of ice-water mixture. The mixture was neutralised with saturate aqueous $NaHCO_3$ solution and extracted with ethyl acetate (3 × 30 mL). The combined organic layer was washed with brine, dried with anhydrous Na_2SO_4 and evaporated to dryness to give an oil which solidified (white) upon keeping overnight. Yield: 1.06 g (80%). ^{1}H NMR (400 MHz, $CDCl_3$) δ: 9.31 (dd, J_1 = 2 Hz, J_2 = 0.9 Hz, 1H, PyH6), 8.44 (dd, J_1 = 8.1 Hz, J_2 = 2.1 Hz, 1H, PyH4), 8.44 (dd, J_1 = 8.1 Hz, J_2 = 0.8 Hz, 1H, PyH3), 4.53 (q, J = 7.2 Hz, 4H, CH_2), 4.47 (q, J = 7.2 Hz, 4H, CH_2), 1.47 (t, J = 7.2 Hz, 3H, CH_3), 1.44 (t, J = 7.2 Hz, 3H, CH_3). ESI-MS (+ve) *m*/*z* found for $[C_{11}H_{13}NO_4 + H]^+$ 224.09, calcd. 224.09.

2,5-Dihydroxymethylpyridine: Diethyl pyridine-2,5-dicarboxylate (7.31 g, 32.7 mmol) was dissolved in degassed EtOH (100 mL) and cooled to 5 °C. $NaBH_4$ (5.00 g, 132 mmol) was added portion-wise over 45 min to form a yellow solution which was stirred for 90 min before stirring for 3 h at room temperature. The solution was then heated to reflux at 80 °C for 15 h before cooling to room temperature and the solvent removed under vacuo to leave a yellow-white residue. The residue was suspended in acetone (110 mL) and saturated aqueous K_2CO_3 (100 mL) before heating to reflux at 70 °C for 1 h. After cooling to room temperature, a clear, yellow organic layer was collected, and the solvent removed under vacuo to leave a yellow oil (11.12 g). The crude product was purified by silica gel column chromatography (7: 1 mixture of DCM and MeOH) to yield yellow oil. Yield: 4.3 g (93%). ^{1}H NMR (400 MHz, Methanol-d_4) δ: 8.44 (dd, J_1 = 2.2 Hz, J_2 = 0.9 Hz, 1H, PyH6), 7.84 (dd, J_1 = 8.0 Hz, J_2 = 2.2 Hz, 1H, PyH4), 7.53 (d, J = 8.0 Hz, ^{1}H, PyH3), 4.69 (s, 2H, CH_2), 4.64 (s, 2H, CH_2). ESI-MS (+ve) *m*/*z* found for $[C_7H_9NO_2 + H]^+$ 140.2, calcd.140.1.

5-Hydroxymethyl-2-pyridinecarboxaldehyde: 2,5-Dihydroxymethylpyridine (2.78 g, 20 mmol) and SeO_2 (1.05 g, 10 mmol) were dissolved in degassed dioxane (20 mL) and water (0.5 mL) under an argon atmosphere. The solution was heated to reflux at 100 °C for 3 hours. The solution was allowed to cool to room temperature, filtered, and washed with minimal dioxane until the filtrate ran clear. The solution

was concentrated (to ≈10 mL) and purified by silica gel column chromatography (50-75% ethyl acetate in hexane). The solvent was removed from the product fractions under vacuo to yield a white powder product. Yield: 1.89 g (69%). ^{1}H NMR (400 MHz, DMSO-d_6) δ: 9.98 (d, J = 0.8 Hz, 1H, CHO), 8.76 (m, 1H, PyH6), 7.97 (m, 1H, PyH4), 7.92 (m, 1H, PyH3), 7.44 (dd, J_1 = 8.0 Hz, J_2 = 0.9 Hz, 1H, PyH3), 5.55 (t, J = 5.6 Hz, 3H, OH), 4.66 (d, J = 5.6 Hz, 2H, CH_2). ESI-MS (+ve) m/z found for $[C_7H_7NO_2 + H]^+$ 138.06, calcd. 138.06.

L1: Addition of 5-Hydroxymethyl-2-pyridinecarboxaldehyde (112 mg, 0.82 mmol) to a solution of 4,4-methylenedianiline (MDA, 81 mg, 0.41 mmol) in ethanol (10 mL) resulted an instant off-white precipitate. The precipitate was collected by filtration after stirring the mixture for another 2.5 h at room temperature followed by washing with ethanol and diethyl ether. Yield: 116 mg (65%). ^{1}H NMR (400 MHz, DMSO-d_6) δ: 8.65 (m, 2H, PyH6), 8.59 (s, 2H, CH^{Imine}), 8.13 (m, 2H, PyH4), 7.89 (m, 2H, PyH3), 7.34 (m, 8H, ArH^{MDA}), 5.46 (t, 2H, J = 6 Hz, OH), 4.62 (d, 2H, J = 6 Hz, $PyCH_2$), 4.01 (s, 2H, CH_2^{MDA}). ESI-MS (+ve) m/z found for $[C_{27}H_{24}N_4O_2 + H]^+$ 437.20, calcd. 437.20.

6-Hydroxy-4,4,5,7,8-pentamethylhydrocoumarin: The synthesis was carried out according to the reported literature procedure.[5] In brief, mixture of trimethylhydroquinone (1.02 g, 6.7 mmol) and methyl 3,3-dimethylacrylate (1 mL, 7.6 mmol) were heated in methanesulfonic acid (15 mL) at 80 °C. After 2 h, the deep reddish-brown solution was cooled to room temperature and poured into 200 mL cold water resulting in a light brown suspension. It was then extracted with ethyl acetate (4 × 50 mL). The combined organic layers were washed with water, saturated aqueous NaHCO3, water respectively followed by a final wash with brine. The organic layer was dried with anhydrous Na_2SO_4 and evaporated to dryness in a rotary evaporator. The residue was recrystallised with chloroform/hexane (1:1) to afford off-white crystalline solid, washed with hexane and dried in vacuum. Yield: 1.3 g (82.8%). ^{1}H NMR (400 MHz, $CDCl_3$) δ: 4.55 (s, 1H, OH), 2.55 (s, 2H, CH_2), 2.36 (s, 3H, CH_3), 2.22 (s, 3H, CH_3), 2.18 (s, 3H, CH_3), 1.45 (s, 6H, CH_3). ESI-MS (−ve) m/z found for $[C_{14}H_{18}O_3 - H]^-$ 233.12, calcd. 233.12.

3-Methyl-3-(2,4,5-trimethyl-3,6-dioxocyclohexa-1,4-dien-1-yl)butanoic acid (QPA): QPA was synthesised from 6-hydroxy-4,4,5,7,8-pentamethylhydrocoumarin following a procedure reported in literature.[6] To a solution of lactone (1.85 g, 7.9 mmol) in 10% aqueous acetonitrile (100 mL), solution of N-bromo succinimide (1.48 g, 8.3 mmol) in acetonitrile (15 mL) was added. The solution turned quickly to yellow and was stirred at room temperature for 2 h. After that the solution was added to water (400 mL) giving a yellow suspension which is then extracted with ethyl acetate (4 × 80 mL). The organic layer was dried with anhydrous Na_2SO_4 and evaporated to dryness to give yellow solid of QPA as quantitative yield and used for the next step without further purification. ^{1}H NMR (400 MHz, $CDCl_3$) δ: 3.02 (s, 2H, CH_2), 2.14 (s, 3H, CH_3), 1.96 (m, 3H, CH_3), 1.93 (m, 3H, CH_3), 1.44 (s, 6H, CH_3). ESI-MS (−ve) m/z found for $[C_{14}H_{18}O_4 - H]^-$ 249.11, calcd. 249.11.

Esterification of QPA with 5-hydroxymethyl-2-pyridinecarboxaldehyde: 5-hydroxymethyl-2-pyridinecarboxaldehyde (69 mg, 0.5 mmol) was suspended in a solution of QPA (125 mg, 0.5 mmol) in DCM (15 mL) to which EDC.HCl (125 mg, 0.65 mmol) and DMAP (14 mg, 0.11 mmol) were added at ice-cold condition. The reaction mixture was slowly warmed to room temperature and the suspension of aldehyde dissolved. After stirring overnight, the reaction mixture was concentrated in vacuum and loaded on a silica gel column and eluted with 5% ethyl acetate in DCM to afford the desired QPA substituted aldehyde (R_f = 0.4) as yellow oil which solidified upon keeping overnight in refrigerator. Yield: 130 mg (70%). ^{1}H NMR (300 MHz, $CDCl_3$) δ: 10.08 (d, 1H, J = 1 Hz, CHO), 8.71 (m, 1H, PyH6), 7.96 (m, 1H, PyH4), 7.81 (m, 2H, PyH3), 5.12 (s, 2H, $PyCH_2$), 3.08 (s, 2H, CH_2^{QPA}), 2.13 (s, 3H, CH_3^{QPA}), 1.94 (q, J = 1.1 Hz, 3H, CH_3^{QPA}), 1.87 (q, J = 1.1 Hz, 3H, CH_3^{QPA}), 1.44 (s, 6H, CH_3^{QPA}). ESI-MS (+ve) m/z found for $[C_{21}H_{23}NO_5 + Na]^+$ 392.1486, calcd. 392.1474.

L2: To a solution of QPA derivatised aldehyde (38 mg, 0.1 mmol) in ethanol (5 mL), MDA (10.2 mg, 0.05 mmol) was added and stirred overnight. Evaporation of the solvent gave L1 as oily residue which was washed once with cold ethanol followed by diethyl ether and used for metal complexation without further purification. ^{1}H NMR (300 MHz, CD_2Cl_2) δ: 8.58 (m, 4H, CH^{Imine} & PyH6), 8.18 (m, 2H, PyH4), 7.74 (m, 2H, PyH3), 7.31 (m, 8H, ArH^{MDA}), 5.07 (s, 4H, $PyCH_2$), 4.05 (s, 2H, CH_2^{MDA}), 3.03 (s, 4H, CH_2^{QPA}), 2.10 (s, 6H, CH_3^{QPA}), 1.91 (q, J = 1.1 Hz, 6H, CH_3^{QPA}), 1.86 (q, J = 1.1 Hz, 6H, CH_3^{QPA}), 1.43 (s, 12H, CH_3^{QPA}). ESI-MS (+ve) m/z found for $[C_{55}H_{56}N_4O_8 + H]^+$ 901.4416, calcd. 901.4176.

$[Fe_2(L2)_3](PF_6)_4$: $FeCl_2.4H_2O$ (6.7 mg, 0.034 mmol) and L2 (47 mg, 0.052 mmol) were dissolved in methanol (10 mL). The deep purple solution was then refluxed for 2.5 h at 70 °C. After cooling the reaction mixture at room temperature, the volume of the mixture was reduced to half and treated with

NH_4PF_6 (150 mg, 0.92 mmol). After keeping overnight in a refrigerator, the purple precipitate of $[Fe_2(L2)_3](PF_6)_4$ was vacuum filtered over 0.45 µm nylon filter paper, washed with cold methanol and finally with diethyl ether. Yield: 50 mg (88%). ^1H NMR (500 MHz, CD_2Cl_2) *δ*: 8.96 (s, 6H, CH^{Imine}), 8.57 (bs, 6H, PyH3), 8.26 (bs, 6H, PyH4), 7.34 (s, 6H, PyH6), 6.96 (br, 12H, ArH^{MDA}), 5.49 (br, 12H, ArH^{MDA}), 5.16 (m, 12H, $PyCH_2$), 4.02 (s, 6H, CH_2^{MDA}), 3.04 (m, 12H, CH_2^{QPA}), 2.10 (s, 18H, CH_3^{QPA}), 1.92 (s, 18H, CH_3^{QPA}), 1.83 (s, 18H, CH_3^{QPA}), 1.40 (s, 36H, CH_3^{QPA}). ESI-MS (+ve) *m/z* found for $[C_{165}H_{168}Fe_2N_{12}O_{24}]^{4+}$ 703.5320, calcd. 703.5254. UV-Vis (CH_3CN) [λ_{max}, nm (ε, M^{-1} cm^{-1})]: 576 (17700), 327 (44900), 259 (166400).

$[Fe_2(L2)_3]Cl_4$: $[Fe_2(L2)_3](PF_6)_4$ (20 mg, 0.007 mmol) was suspended in methanol (10 mL) and stirred at room temperature in presence of Dowex® 1X8 chloride resin (200-400 mesh) until it gets dissolved (~2 h). After that the mixture was filtered to remove the ion exchange resin and filtrate was evaporated to dryness to afford $[Fe_2(L2)_3]Cl_4$. ^1H NMR (400 MHz, CD_3OD) *δ*: 9.21 (s, 6H, CH^{Imine}), 8.74 (d, 6H, *J* = 8 Hz, PyH3), 8.43 (d, 6H, *J* = 8 Hz, PyH4), 7.39 (s, 6H, PyH6), 7.04 (br, 12H, ArH^{MDA}), 5.60 (br, 12H, ArH^{MDA}), 5.24 (m, 12H, $PyCH_2$), 4.07 (s, 6H, CH_2^{MDA}), 2.97 (s, 12H, CH_2^{QPA}), 2.11 (s, 18H, CH_3^{QPA}), 1.93 (s, 18H, CH_3^{QPA}), 1.83 (s, 18H, CH_3^{QPA}), 1.43 (m, 36H, CH_3^{QPA}). ESI-MS (+ve) *m/z* found for $[C_{165}H_{168}Fe_2N_{12}O_{24}]^{4+}$ 703.54, calcd. 703.53. UV-Vis (CH_3OH) [λ_{max}, nm (ε, M^{-1} cm^{-1})]: 575 (13600), 327 (44000), 259 (163900).

$[Ni_2(L2)_3]Cl_4$: $[Ni_2(L2)_3](PF_6)_4$ was synthesised following the same procedure (refluxing a 2:3 mixture of $NiCl_2.6H_2O$ & L2 for 2.5 h in methanol followed by treatment with NH_4PF_6) as for iron cylinder to give the $[Ni_2(L2)_3](PF_6)_4$ as light orange solid. Yield: 55 mg (94%). ESI-MS (+ve) *m/z* found for $[C_{165}H_{168}N_{12}Ni_2O_{24}]^{4+}$ 704.9565, calcd. 704.5259. UV-Vis (CH_3CN) [λ_{max}, nm (ε, M^{-1} cm^{-1})]: 327 (64400), 258 (179600).
It was then exchanged with anion exchange resin. ESI-MS (+ve) *m/z* found for $[C_{165}H_{168}N_{12}Ni_2O_{24}]^{4+}$ 704.6996, calcd. 704.5259. UV-Vis (CH_3OH) [λ_{max}, nm (ε, M^{-1} cm^{-1})]: 327 (75100), 258 (223800). Elemental analysis (%) calculated for $C_{165}H_{168}Cl_4N_{12}Ni_2O_{24}$ $.6H_2O$: C 64.54, H 5.91, N 5.47, found C 64.65, H 5.74, N 5.60.

$[Fe_2(L1)_3]Cl_4$: $FeCl_2.4H_2O$ (14 mg, 0.07 mmol) and L1 (50 mg, 0.11 mmol) were dissolved in methanol (5 mL). The deep purple solution was then refluxed overnight at 70 °C. After cooling the reaction mixture at room temperature, the volume of the mixture was reduced to half and treated with NH_4PF_6 (114 mg, 0.70 mmol). After keeping overnight in a refrigerator, the purple precipitate of $[Fe_2(L1)_3](PF_6)_4$ was vacuum filtered over 0.45 µm nylon filter paper, washed with cold methanol and finally with diethyl ether. Yield 60 mg (86%). ^1H NMR (400 MHz, CD_3CN) *δ*: 8.89 (s, 6H, CH^{Imine}), 8.50 (d, 6H, *J* = 8 Hz, PyH3), 8.29 (d, 6H, *J* = 8 Hz, PyH4), 7.24 (s, 6H, PyH6), 6.92 (br, 12H, ArH^{MDA}), 5.53 (br, 12H, ArH^{MDA}), 4.66 (d, *J* = 8 Hz, 12H, $PyCH_2$), 4.01 (s, 6H, CH_2^{MDA}), 3.79 (t, 6H, *J* = 6 Hz, OH). ESI-MS (+ve) *m/z* found for $[C_{81}H_{72}Fe_2N_{12}O_6]^{4+}$ 355.3654, calcd. 355.1096. UV-Vis (CH_3CN) [λ_{max}, nm (ε, M^{-1} cm^{-1})]: 572 (17700), 324 (43600), 286 (87500).
It was then exchanged with anion exchange resin. ^1H NMR (400 MHz, CD_3OD) *δ*: 9.11 (s, 6H, CH^{Imine}), 8.64 (d, 6H, *J* = 8 Hz, PyH3), 8.39 (m, 6H, PyH4), 7.39 (s, 6H, PyH6), 7.00 (br, 12H, ArH^{MDA}), 5.61 (br, 12H, ArH^{MDA}), 4.74 (s, 12H, $PyCH_2$), 4.04 (s, 6H, CH_2^{MDA}). ESI-MS (+ve) *m/z* found for $[C_{81}H_{72}Fe_2N_{12}O_6]_{4+}$ 355.11, calcd. 355.11. UV-Vis (CH_3OH) [λ_{max}, nm (ε, M^{-1} cm^{-1})]: 572 (16000), 324 (37200), 287 (79800).

$[Ni_2(L1)_3]Cl_4$: $[Ni_2(L1)_3](PF_6)_4$ was synthesised as same as previously stated (refluxing a 2:3 mixture of $NiCl_2.6H_2O$ & L1 for overnight in methanol followed by treatment with NH_4PF_6). Yield 91%. ESI-MS (+ve) *m/z* found for $[C_{81}H_{72}N_{12}Ni_2O_6]^{4+}$ 356.62, calcd. 356.11. UV-Vis (CH_3CN) [λ_{max}, nm (ε, M^{-1} cm^{-1})]: 293 (63050), 249 (76600).
It was then exchanged with anion exchange resin. ESI-MS (+ve) *m/z* found for $[C_{81}H_{72}N_{12}Ni_2O_6]^{4+}$ 356.6034, calcd. 356.1101. UV-Vis (CH_3OH) [λ_{max}, nm (ε, M^{-1} cm^{-1})]: 293 (63000), 248 (77300). Elemental analysis (%) calculated for $C_{81}H_{72}Cl_4N_{12}O_6Ni_2.9H_2O$: C 56.21, H 5.24, N 9.71, found C 56.26, H 5.05, N 9.48.

Ethyl 3-methyl-3-(2,4,5-trimethyl-3,6-dioxocyclohexa-1,4-dien-1-yl)butanoate (QPA Et): QPA (60 mg, 0.24 mmol) was mixed with EtOH (1 mL) in DCM (20 mL) in the presence of EDC.HCl (60 mg, 0.31 mmol) and DMAP (3 mg, 0.02 mmol) at ice-cold condition. The reaction mixture was slowly warmed to room temperature. After stirring overnight, the reaction mixture was concentrated in vacuum and loaded on a silica gel flash column and eluted by hexane with increasing gradient of ethyl acetate to afford the desired esterified QPA (R_f = 0.6, ethyl acetate : hexane = 1 : 1) as yellow gummy liquid. Yield: 60 mg (88%). ^1H NMR (400 MHz, $CDCl_3$) *δ*: 4.04 (q, *J* = 8 Hz, 2H, CH_2^{Et}), 2.95 (s, 3H, CH_2^{QPA}), 2.13 (s, 3H,

CH_3^{QPA}), 1.95 (s, 6H, CH_3^{QPA}), 1.42 (s, 6H, CH_3^{QPA}), 1.19 (t, *J* = 8 Hz, 3H, CH_3^{Et}). ESI-MS (+ve) *m/z* found for $[C_{21}H_{23}NO_5 + Na]^+$ 301.1470, calcd. 301.1416.

Biophysical Experiments

Polyacrylamide Gel Electrophoresis (PAGE)

Gels (12% PAGE, dimension 20 cm × 20 cm) were prepared freshly before every experiment by mixing 20 mL of 37.5:1 acrylamide/bis-acrylamide mixture, 30 wt% in water (Sigma Aldrich), 25 mL of water, 5 mL of 10X Tris-Acetate (TA) buffer (400 mM of tris base, Sigma Aldrich) of pH 7.4 (adjusted with acetic acid, Fisher Scientific) followed by the addition of 400 µL of 10% w/v Ammonium Persulfate solution in water and 40 µL of Tetramethylethylenediamine (TEMED, Fisher Scientific). The whole mixture was poured quickly between two vertically standing glass plates and then a 20-well comb was inserted from the top. The gel was allowed to set for ~1.5 h, after which the comb was removed and the gel placed in the running chamber. Metallo-cylinder stocks were made in 5% methanol in water (100 µM) and stored at −20 °C. NQO1 (Sigma Aldrich) stocks (1 mg/mL, 33.33 µM) were made in a mixture containing Tris-HCl (20 mM, pH 8.0), dithiothreitol (DTT, 1 mM), Bovine Serum Albumin (BSA , 0.1%) & Glycerol (10%) and stored at −20 °C. Reduced Nicotinamide Adenine Dinucleotide Phosphate (NADPH, Sigma Aldrich) and Glutathione (GSH, Sigma Aldrich) stock were freshly prepared in water before each experiment. Dicoumarol (Chembridge BioScience) stock (1 mM) was also freshly prepared in 15% DMSO-water mixture just before the experiment. QPA Et stock was dissolved in 20% methanol in water and diluted with water to make a final stock for gel loading (1.8% methanol in water).14-mer oligonucleotide sequences (200 µM, Sigma Aldrich) S1 (5′-CGGAACGGCACTCG-3′), S2 (5′- CGAGTGCAGCGTGG-3′) & S3 (5′-CCACGCTCGTTCCG-3′) were mixed with water to make a stock of 10 µM and stored at −20 °C. Samples were made in 1X TA (pH 7.4) buffer with required components (metal complex, DNA oligonucleotide mix, enzyme, enzyme inhibitor and reducing agent). 1X loading dye in 1X TA (pH 7.4) buffer was made from the stock (6X, Thermo Fisher Scientific). The sample mixtures contained the desired stoichiometry (0.5 – 4 eqv) of metallo-cylinder with respect to DNA (1 µM). Unless stated otherwise, the final concentration of NQO1 in each sample mixture was 50 µg/mL and NADPH was used at a 100-fold molar equivalent with respect to the concentration of metallo-cylinder. Dicoumarol was used in the sample mixture at a fixed concentration of 50 µM. After incubating in dark conditions at 37 °C, the sample mixture (30 µL) was mixed with 7.5 µL of 50% glycerol-water before loading into the gel (10 µL) and run for 2.5 h at 140 V. The same 1X TA (pH 7.4) buffer was used as running buffer. An ice-cold water jacket was used throughout the run to prevent the gel from overheating. The gel was then taken out from the glass plates and stained by SYBR™ Gold Nucleic Acid Gel Stain (Thermo Fisher Scientific) in 1X TA (pH 7.4) buffer for 40 minutes before imaging on a gel imaging system with 302 nm excitation.

For 14-mer RNA S1: (5′-CGGAACGGCACUCG-3′), S2: (′-CGAGUGCAGCGUGG-3′) & (5′-CCACGCUCGUUCCG-3′), 1X TA (10 mM NaCl, pH 7.4), smaple incubation time (2 h, 37 ℃) and gel (12% PAGE, dimension 20 cm × 20 cm) running time followed same as above.

For 18-mer DNA S4: (5′-GTGGCGAGAGCGACGATC-3′), S5: (5′-GATCGTCGCAGAGTTGAC-3′) & S6: (5′-GTCAACTCTTCTCGCCAC-3′), mini gels (12% PAGE, dimension 8.3 cm × 7.3 cm) were prepared by mixing 6 mL of 37.5:1 acrylamide/bis-acrylamide with 1.5 mL of 10X TA buffer (400 mM of tris base, Sigma Aldrich) of pH 7.4 (adjusted with acetic acid, Fisher Scientific) and 7.5 mL of Milli-Q water. To this 150 µL of a 10% w/v ammonium persulfate solution in water and 15 µL of TEMED were added to initialise polymerisation. This was then immediately poured between 2 glass plates and a 10-well comb inserted at the top; this was then allowed to set for 1 h before proceeding. Same conditions were used to make the sample and pipetted (10 µL) into the wells on the gel. The gels were run at 140 V for 35 mins at room temperature (20 °C) followed by staining SYBR™ Gold Nucleic Acid Gel Stain (Thermo Fisher Scientific) in 1X TA (pH 7.4) buffer for 40 minutes before imaging on a gel imaging system with 302 nm excitation.

Fluorescent PAGE experiments

For the variable time PAGE study, we used a fluorescently labelled 3WJ with FAM (6- Fluorescein Phosphoramidite) on S1 (5′ 6-FAM-CGGAACGGCACTCG-3′), while the other strands consist of S2 (5′-CGAGTGCAGCGTGG-3′) and S3 (5′-CCACGCTCGTTCCG-3′). The samples were removed from incubation at various time points, mixed with 50% glycerol-water, and then frozen at −20 °C. Once the incubation of all samples was complete, they are loaded onto a PAGE gel (12%, dimension 20 cm × 20 cm) as previously described. The intensity of the bands for the FAM-labelled 3WJ and frontrunner S1 was then calculated using ImageJ Fiji software.

UV melting experiments
The stability of the DNA and RNA three-way junctions (3WJ) with the capped and uncapped cylinders was monitored by measuring the absorbance at 260 nm with increasing temperature. The DNA 3WJ structure was composed of 3 separate 18-mer oligonucleotides and the RNA 3WJ was composed of 3 separate 14-mer oligonucleotides. Both junctions form spontaneously in the absence of binding agents. Each sample contained 1 µM of each oligo, 1 µM of either capped or uncapped cylinder, 0.05% MeOH, 10 mM sodium cacodylate (pH 7.4) and 100 mM NaCl. Control samples were also prepared containing all components except the cylinder. Samples were made up to 1500 µL volume and pipetted into 1500 µL masked quartz cuvettes with 1 cm path length and the cuvette then stoppered. The measurements were carried out on a Cary 5000 UV-Vis-NIR spectrophotometer (bandwidth, 1 nm; average time 1 s; heating rate, 0.5˚ C min^{-1}; measurement interval, 0.5 ˚C) equipped with a multi-cell holder and peltier temperature controller. Data was collected in triplicate for each condition and a blank sample was ran concurrently allowing for immediate baseline correction. The data was normalised and plotted in MATLAB, and the melting temperature (T_m) determined as the temperature at which 50% of the DNA/RNA 3WJ is unfolded. The final melting temperature was then reported as the average of the three runs.

DNA 3WJ S1: 5′-GTGGCGAGAGCGACGATC-3′
DNA 3WJ S2: 5′-GATCGTCGCAGAGTTGAC-3′
DNA 3WJ S3: 5′-GTCAACTCTTCTCGCCAC-3′
RNA 3WJ S1: 5′-CGGAACGGCACUCG-3′
RNA 3WJ S2: 5′-CGAGUGCAGCGUGG-3′
RNA 3WJ S3: 5′-CCACGCUCGUUCCG-3′

Circular Dichroism (CD)
CD titrations were carried out at room temperature by adding a solution of complex (100 µM in 5% methanol in water) into a solution of calf thymus, ctDNA (100 µM in 1 mM sodium cacodylate buffer of pH 7.4 containing 10 mM NaCl). ctDNA solution (900 µL) was recorded in a cuvette (1 cm path length) and scanned from 500 nm to 200 nm with 3 repeats at 1 nm step size and 0.6 s dwell time per point. After that the concentration of complex in cuvette was increased stepwise with respect to the ctDNA. An equal volume of 2X ctDNA solution (200 µM in 2 mM sodium cacodylate buffer of pH 7.4 containing 20 mM NaCl) was added simultaneously into the cuvettewith addition to maintain constant concentration of ctDNA. CD spectra were background corrected by subtracting CD spectrum of titration buffer solution (1 mM sodium cacodylate buffer of pH 7.4 containing 10 mM NaCl). CD spectra containing different R values were plotted using OriginPro® 2021 where R value refers to the ratio of DNA base pairs to complex, i.e. R100 means 100 bp of ctDNA for every 1 complex, R30 means 30 bp of ctDNA per complex and so on.

Flow Linear Dichroism (LD)
LD was carried out at the same concentration of complex, ctDNA and buffer as in CD experiments. The LD cell has an angular gap of 0.25 mm giving an overall path length of 0.5 mm. 150 µl of ctDNA (100 µM in 1 mM sodium cacodylate buffer of pH 7.4 containing 10 mM NaCl) was dispensed into the couette cell and rotated at 40 revolutions per second to optimise the DNA signal and the LD was recorded scanning from 500 nm to 200 nm with 3 repeats at 1 nm step size and 0.6 s dwell time per point. The titration series was carried out in the same way as in the CD studies by increasing the ratio of metallo-cylinder with respect to ctDNA (with addition of doubly concentrated ctDNA taken in doubly concentrated working buffer) with a 4-minute incubation (room temperature) time at a lower revolution speed of 4 revolutions per second. The rotation speed was escalated to 40 revolutions per second when recording LD. Background subtraction from each LD spectra used the LD of ctDNA (100 µM in 1 mM sodium cacodylate buffer of pH 7.4 containing 10 mM NaCl) recorded without rotation of the couette.

Stability Study
The aqueous stability of the metallo-cylinder (5% methanol-water stock) was carried out in 1X TA buffer (pH 7.4) at 37 °C and UV-vis spectra were recorded at different time periods scanning from 800 nm to 200 nm (1 nm bandwidth, 600 nm/min).

ESI-MS Speciation Study
A mixture containing Ni-cylinder (50 μM diluted from a 5% methanol-water 100 μM stock), NQO1 (20 μg/mL) and NADPH (400 μM) was incubated at 37 °C in water and ESI-MS spectra were recorded at various time points. Identical conditions were followed in the studies without NQO1.

Molecular Dynamics Simulations

Parameterisation of Metal Compounds

The coordinates for the iron uncapped cylinder were generated using molecular editing software Avogadro[7] from the crystal structure of the iron parent cylinder reported previously,[8] by attaching a hydroxyl group in the 5 position on each of the coordinating pyridyl groups. Parameters were then calculated using the MCPB.py pipeline with Gaussian16 at the ωB97XD/DEF2 SVP level of theory to include dispersion as previously described.[9]

Parameterisation of the capped cylinder posed a challenge as the lack of an experimental structure, and the conformational freedom in the QPA moiety made DFT convergence difficult. The initial coordinates for the iron capped cylinder were generated in Avogadro from the geometry-optimised uncapped cylinder, by attaching the QPA groups at the 5-pyridyl oxygen. In order to first reduce the computational expense, a half cylinder (see figure SX) was geometry optimised in ORCA[10] at the ωB97XD3/DEF2-SVP level of theory.[11] This allowed the 3 caps on one side to reposition into more favourable geometries. The half cylinder was then combined with itself at the central CH_2 linkers to generate a full cylinder using Avogadro, which then underwent parameterisation in the MCPB.py pipeline at the ωB97XD/DEF2-SVP level of theory, as described previously. The output coordinate and topology files were converted to GROMACS format using ParmEd (https://github.com/ParmEd/ParmEd).

Parameterisation of DNA
The PDB file for the 25-mer dsDNA consisting of 2 strands (A25 and T25) was generated using NAB (nucleic acid builder) in AmberTools.[11] The 3WJ structure was adapted from PDB 1F44, as described previously.[12] All DNA was parameterised using the AMBER forcefield parmbsc1.[13]

Simulations
In all simulations of 3WJ, the compounds were placed directly inside the 3WJ cavity. In all simulations with dsDNA, multiple compounds were placed within 1 nm distance of the DNA. In all simulations, cylinders were placed with (or without) DNA in a dodecahedral box with periodic boundary conditions. MD preparation steps were carried out as described previously[14] using GROMACS software (2023).[15] All systems were solvated in water using the TIP3P model and neutralised with Na+ ions. Additional Na+ and Cl- ions were added to reach a NaCl concentration of 50 mM. Initial minimisation was carried to at least 500 kJ/mol/nm or 50000 steps followed by heating and NVT equilibration for 1000 ps using V-rescale modified Berendsen thermostat, coupling the cylinder with the DNA at 310 K. All simulations use a time step of 2 fs and Parrinello-Rahman pressure coupling and PME electrostatics at 1.0 nm cutoff. All simulations were run on the BlueBEAR HPC cluster, which provides a high-performance computing service at the University of Birmingham. After the simulations had finished, the trajectories were processed in GROMACS to remove periodic boundary conditions, translations and rotations, and visualised in PyMOL.[16]

Cell Line and Culture

Human lung adenocarcinoma epithelial cells (A549) were procured from UKHSA (Catalogue No. 86012804) and grown as a monolayer in a humidified incubator (95% air, 5% CO_2) at 37 °C. Cells were cultured in a T_{75} flask and subcultured twice a week in Dulbecco's Modified Eagle Medium (DMEM) supplemented with 10% (v/v) fetal bovine serum (FBS), 1% L-glutamine (2 mM), 100 U/mL of penicillin and 100 μg/mL of streptomycin.

Antiproliferative Activity

The growth inhibitory effect against cancer cell lines was assessed by MTT assay. 5000 cells were seeded in a 96-well plate taken in 100 µL volume of DMEM and incubated at the aforesaid incubating condition. After 24 h, media was removed and renewed with 100 µL of fresh media follwed by the treament of the required concentration of compounds. Fresh stock of the cylinders were made in methanol/DMEM mixture and serially diluted with DMEM before adding in the wells. Each concentration was added in triplicate. The maximum methanol concencentration in well (0.9%, v/v) was used as negative control to determine IC_{50} value, which represents the concentration of the compound needed for 50% cells growth inhibition with respect to the negative control. Cisplatin (positive control) stock and dilution were made in Dulbecco's phosphate-buffered saline (DPBS, pH 7.0 to 7.3 without calcium, magnesium and phenol red). After completion of 72 h incubation with the compounds, the media was removed and replaced with 100 µL fresh media containing 0.5 mg/mL 3-(4,5-Dimethylthiazol-2-yl)-2,5-Diphenyltetrazolium Bromide (MTT) (diluted from a stock of 5 mg/mL in DPBS) and incubated for 2.5 h in the CO_2 incubator (95% air, 5% CO_2) at 37 °C. Following removal of the media, the resulting formazan crystals were dissolved in DMSO (185 µL) and absorbances were recorded at 595 nm. The IC_{50} value was calculated by fitting nonlinear curves with a variable slope model in GraphPad Prism®, constructed by plotting cell viability (%) *vs* log of drug concentration in nM. Each independent experiment was carried out in triplicate and the values were provided in mean ± standard deivation (SD). For determining the IC_{50} in precence of NQO1 inhibitor, cells were treated with 25 µM (or 50 µM) of dicoumarol (dissolved in DMSO/DMEM mixture) for 2 h following 24 h of cell seeding. Test compounds were co-treated after that accordingly. Dicoumarol treated control wells which contain DMSO (1%, v/v) as well as methanol (0.9%, v/v) were considered as negative control (loss of viability of dicoumarol treated control cells was small with respect to untreated control cells – see Table S2) during IC_{50} calculation as above. Statistical significance was carried out in Graph pad prism® software using one-way ANOVA followed by a Dunnett's *post hoc* t-test.

Cellular internalisation

1 × 10^6 A549 cells were seaded in a 60 mm dia petridish. After 24 h, the media was replaced with 5 mL of fresh media followed by the addition of stock solution of individual cylinders prepared freshly in MeOH/DMEM mixture to achieve final concentration of 1.5 µM in petridish. Control cells were treated with identical MeOH/DMEM mixture to achieve final MeOH concentration of ≈0.2% in petridish. Afrer 24 h, drug containing media was discarded and cells were washed thrice with 1 mL of DPBS followd by trypsinization. 1 × 10^6 live cells were counted and pelleted down using centrufugation (1500 g for 10 min). Cell pellets were washed again with DPBS (1 mL) and dried overnight under a fume cupboard. In the next day cells were digested with ICP-MS grade concentrated HNO_3 (200 µL) for overnight at room temperature and then diluted with 12 mL of MiliQ water. A blank sample solution was prepared of the same dilution of HNO_3 and Ni content in the sample were analysed in ICP-MS (Agilent 7500ce) based on the calibration curve made with standard solution of Ni (0.1 ppb, 0.25 ppb, 0.5 ppb, 1 ppb, 5 ppb, 10 ppb, 25 ppb, 50 ppb and 100 ppb).

Author Contributions

MJH and SK conceived the project and designed experiments. MJH supervised and directed the studies, and NJH supervised the cell studies. SK undertook synthesis, gel, spectroscopic experiments and cell studies. SJD undertook the MD simulations. CAJH undertook some of the synthesis. SK and MJH drafted the manuscript which all authors commented on.

References

[1] a) B. Nordén, T. Kurucsev, *J. Mol. Recognit.* **1994**, *7*, 141-155; b) A. Rodger, B. Norden, *Circular Dichroism and Linear Dichroism*, Oxford Univ Press, **1997**; c) M. Vorlíčková, I. Kejnovská, K. Bednářová, D. Renčiuk, J. Kypr, *Chirality* **2012**, *24*, 691-698.

[2] a) M. J. Hannon, V. Moreno, M. J. Prieto, E. Moldrheim, E. Sletten, I. Meistermann, C. J. Isaac, K. J. Sanders, A. Rodger, *Angew. Chem. Int. Ed.* **2001**, *40*, 879-884; b) I. Meistermann, V. Moreno, M. J. Prieto, E. Moldrheim, E. Sletten, S. Khalid, P. M. Rodger, J. C. Peberdy, C. J. Isaac, A. Rodger, M. J. Hannon, *Proc. Natl. Acad. Sci. U. S. A.* **2002**, *99*, 5069-5074; c) G. I. Pascu, A. C. G. Hotze, C. Sanchez-Cano, B. M. Kariuki, M. J. Hannon, *Angew. Chem. Int. Ed.* **2007**, *46*, 4374-4378.

[3] G. I. Pascu, PhD thesis, University of Birmingham (UK), **2008**.

[4] A. Lavalette, J. Hamblin, A. Marsh, D. M. Haddleton, M. J. Hannon, *Chem. Commun.* **2002**, 3040-3041.

[5] L. A. Carpino, S. A. Triolo, R. A. Berglund, *J. Org. Chem.* **1989**, *54*, 3303-3310.

[6] R. T. Borchardt, L. A. Cohen, *J. Am. Chem. Soc.* **1972**, *94*, 9175-9182.

[7] M. D. Hanwell, D. E. Curtis, D. C. Lonie, T. Vandermeersch, E. Zurek, G. R. Hutchison, *J. Cheminf.* **2012**, *4*, 17.

[8] J. M. C. A. Kerckhoffs, J. C. Peberdy, I. Meistermann, L. J. Childs, C. J. Isaac, C. R. Pearmund, V. Reudegger, S. Khalid, N. W. Alcock, M. J. Hannon, A. Rodger, *Dalton Trans.* **2007**, 734-742.

[9] a) P. Li, K. M. Merz, Jr., *J. Chem. Inf. Model.* **2016**, *56*, 599-604; b) G. W. T. M. J. Frisch, H. B. Schlegel, G. E. Scuseria, M. A. Robb, J. R. Cheeseman, G. Scalmani, V. Barone, G. A. Petersson, H. Nakatsuji, X. Li, M. Caricato, A. V. Marenich, J. Bloino, B. G. Janesko, R. Gomperts, B. Mennucci, H. P. Hratchian, J. V. Ortiz, A. F. Izmaylov, J. L. Sonnenberg, Williams, F. Ding, F. Lipparini, F. Egidi, J. Goings, B. Peng, A. Petrone, T. Henderson, D. Ranasinghe, V. G. Zakrzewski, J. Gao, N. Rega, G. Zheng, W. Liang, M. Hada, M. Ehara, K. Toyota, R. Fukuda, J. Hasegawa, M. Ishida, T. Nakajima, Y. Honda, O. Kitao, H. Nakai, T. Vreven, K. Throssell, J. A. Montgomery Jr., J. E. Peralta, F. Ogliaro, M. J. Bearpark, J. J. Heyd, E. N. Brothers, K. N. Kudin, V. N. Staroverov, T. A. Keith, R. Kobayashi, J. Normand, K. Raghavachari, A. P. Rendell, J. C. Burant, S. S. Iyengar, J. Tomasi, M. Cossi, J. M. Millam, M. Klene, C. Adamo, R. Cammi, J. W. Ochterski, R. L. Martin, K. Morokuma, O. Farkas, J. B. Foresman, D. J. Fox, Gaussian, Inc, **2016**.

[10] F. Neese, F. Wennmohs, U. Becker, C. Riplinger, *J. Chem. Phys.* **2020**, *152*, 224108.

[11] D. A. Case, H. M. Aktulga, K. Belfon, D. S. Cerutti, G. A. Cisneros, V. W. D. Cruzeiro, N. Forouzesh, T. J. Giese, A. W. Götz, H. Gohlke, S. Izadi, K. Kasavajhala, M. C. Kaymak, E. King, T. Kurtzman, T.-S. Lee, P. Li, J. Liu, T. Luchko, R. Luo, M. Manathunga, M. R. Machado, H. M. Nguyen, K. A. O'Hearn, A. V. Onufriev, F. Pan, S. Pantano, R. Qi, A. Rahnamoun, A. Risheh, S. Schott-Verdugo, A. Shajan, J. Swails, J. Wang, H. Wei, X. Wu, Y. Wu, S. Zhang, S. Zhao, Q. Zhu, T. E. Cheatham, III, D. R. Roe, A. Roitberg, C. Simmerling, D. M. York, M. C. Nagan, K. M. Merz, Jr., *J. Chem. Inf. Model.* **2023**, *63*, 6183-6191.

[12] a) K. C. Woods, S. S. Martin, V. C. Chu, E. P. Baldwin, *J. Mol. Biol.* **2001**, *313*, 49-69; b) J. S. Craig, L. Melidis, H. D. Williams, S. J. Dettmer, A. A. Heidecker, P. J. Altmann, S. Guan, C. Campbell, D. F. Browning, R. K. O. Sigel, S. Johannsen, R. T. Egan, B. Aikman, A. Casini, A. Pöthig, M. J. Hannon, *J. Am. Chem. Soc.* **2023**, *145*, 13570-13580.

[13] I. Ivani, P. D. Dans, A. Noy, A. Pérez, I. Faustino, A. Hospital, J. Walther, P. Andrio, R. Goñi, A. Balaceanu, G. Portella, F. Battistini, J. L. Gelpí, C. González, M. Vendruscolo, C. A. Laughton, S. A. Harris, D. A. Case, M. Orozco, *Nat. Methods* **2016**, *13*, 55-58.

[14] L. Melidis, I. B. Styles, M. J. Hannon, *Chem. Sci.* **2021**, *12*, 7174-7184.

[15] M. J. Abraham, T. Murtola, R. Schulz, S. Páll, J. C. Smith, B. Hess, E. Lindahl, *SoftwareX* **2015**, *1-2*, 19-25.

[16] The PyMOL Molecular Graphics System, Version 2.8, Schrödinger, LLC.
